# Evolution of pathogen-specific improved survivorship post-infection in populations of *Drosophila melanogaster* adapted to larval crowding

**DOI:** 10.1371/journal.pone.0250055

**Published:** 2021-04-14

**Authors:** Rohit Kapila, Mayank Kashyap, Soumyadip Poddar, Shreya Gangwal, N. G. G. Prasad

**Affiliations:** Department of Biological Sciences, Indian Institute of Science Education and Research Mohali, Mohali, India; Inha University, REPUBLIC OF KOREA

## Abstract

The environment experienced by individuals during their juvenile stages has an impact on their adult stages. In holometabolous insects like *Drosophila melanogaster*, most of the resource acquisition for adult stages happens during the larval stages. Larval-crowding is a stressful environment, which exposes the larvae to scarcity of food and accumulation of toxic waste. Since adult traits are contingent upon larval stages, in larval-crowding like conditions, adult traits are prone to get affected. While the effect of resource limited, poor-developmental environment on adult immune response has been widely studied, the effect of adaptation to resource-limited developmental environment has not been studied, therefore in this study we assayed the evolution of ability to survive infection in adult stages as a correlated response to adaptation to larval crowding environments. Using four populations *of Drosophila melanogaster* adapted to larval crowding for 240 generations and their respective control populations, we show that populations adapted to larval crowding show an improved and evolved post-infection survivorship against a gram-negative bacteria *Pseudomonas entomophila*. Whereas, against a gram-positive bacteria *Enterococcus faecalis*, no difference in post-infection survivorship was observed across control and selected populations. In this study, we report the co-related evolution of pathogen-specific increased survivorship post-infection in populations of *Drosophila melanogaster* as a result of adaptation to larval crowding environment.

## Introduction

According to the "Developmental origin of health and disease" theory, nutrition and environment available to an individual during critical developmental stages can have a potentially permanent effect on its ability to defend itself from disease [1]. Moreover, it is now well accepted that early-life environmental conditions and nutritional availability are important factors affecting the level of susceptibility of an adult to disease [2]. Studies investigating immune traits in adult humans suggest that components of the immune system can get permanently programmed by events in early life [2–4]. In both vertebrates [5–7] and invertebrates [8–10], there are many studies that suggest that poor nutritional conditions during development lead to poor adult immune responses.

Invertebrate models are popular for studying the immune response of organisms because they are highly tractable. Among invertebrates, *Drosophila* has been the subject of numerous studies aimed at understanding the immune system and its functioning. In *Drosophila*, being holometabolous, resource acquisition for the adult stage happens during the larval stages. The larval environment is largely confined to the egg-laying site [11], which can lead to resource-limited larval crowding conditions, resulting in decreased nutritional uptake and exposure to a high concentrations of toxic nitrogenous wastes in the environment during larval stages [12–15]. Larval crowding conditions are known to negatively affect different adult traits like adult body size, fecundity, etc. [16–19].

Survivorship post-infection is a resource-intensive trait [20, 21] and it trades-off with competitive ability [22] and life-history traits such as female fecundity, egg viability, male reproductive output [20]. Therefore, in resource-limited conditions like larval crowding, we can expect organisms to have poor immunocompetence in adult stages [22]. Additionally, mounting an immune response involves the generation of non-specific toxins, which poses a risk of damaging the host’s tissues [23, 24]. This can be particularly costly to an organism that has developed under larval crowding and is therefore in poor body condition.

While it is well known that larval crowding can affect subsequent adult traits like adult body size, longevity, fecundity [18, 19], some studies have recently shown that adaptation to larval crowding can result in the correlated *evolution* of a set of adult traits like increased adult longevity, increased pre-copulatory reproductive behaviour [19, 25–28]. However, no study has ever looked into the adult survivorship post-infection as a consequence of adaptation to poor developmental conditions, like larval crowding.

In this study, we used laboratory populations of *Drosophila melanogaster* evolving under larval crowding and their ancestral low larval density controls, to answer the following questions:

1. Do the resources available to an individual during pre-adult stages affect its ability to survive infection in the adult stage?
2. Does *adaptation* to larval crowding result in the evolution of ability to survive infection in the adult stage?

To answer these questions, we used populations of *Drosophila melanogaster* that have evolved for 240 generations in poor developmental (larval-crowding) conditions (MCU populations) and their low-density ancestral controls (MB populations). Larvae from both the regimes were grown at high and low larval densities, and we subsequently measured their survivorship post infection in adult stage with either a gram-negative bacterium *Pseudomonas entomophila* or a gram-positive bacterium *Enterococcus faecalis*. Along with causing severe damage to the gut, both the bacteria are known to cause systemic pathogenic infection [29, 30].

## Materials and methods

We used eight replicate laboratory populations of *Drosophila melanogaster* as our study system. **M**elanogaster **C**rowded as larvae, **U**ncrowded as adults (MCU) and **M**elanogaster **B**aseline (MB) populations. These populations have been described in detail elsewhere [27]. Briefly, the MCU populations are reared at a density of 800 larvae per vial in 1.5 mL of food in 8-dram glass vials, and MBs are maintained at a density of 70 larvae per 6–8 ml of food in 8-dram vials. Adults of these populations are reared in Plexiglas cages (24 cm × 19 cm × 14 cm) at a density of 2400–2800 flies per cage. All the populations are maintained on standard charcoal-cornmeal food on a 21-day discreet generation. MCU and MB populations represented by the same subscript are related by ancestry and, hence, are treated as a statistical block. At the time of the experiment, all the replicate populations of MCU had undergone a selection for almost 240 generations.

### Maintenance regime of control/baseline populations

These populations are same as described in detail in [20]. Briefly, MB (**M**elanogaster **B**aseline) populations (a set of 4 independently maintained populations) are maintained on a 21-day discrete generation cycle on standard cornmeal-charcoal food. Eggs laid by ~12-day-old females are dispensed into 8-dram glass vials containing 6–8 mL of cornmeal-charcoal food at a density of 60–80 eggs per vial. Forty such vials are set up for each of the four replicates. The vials are then incubated at 25°C temperature, 90% RH and constant light. 12 days post-egg collection, when almost all the adults have eclosed, flies are transferred into a Plexiglas cage (24 × 19 × 14 cm) containing a Petri plate of cornmeal-charcoal food and wet absorbent cotton for maintaining high RH levels. Thus, the adult number is approximately 2500 per population per generation. Fresh food plates are provided on every alternate day. On day 18 post-egg collection, the flies are provided with a fresh food plate supplemented with *ad libitum* live yeast paste. Two days later, the flies are provided with a fresh food plate and are allowed to oviposit for 18 h. These eggs are then used to start the next generation.

### Maintenance regime of selected populations

The maintenance regime of MCU is same as described in [27]. Briefly, MCU (**M**elanogaster **C**rowded as larvae and **U**ncrowded as adults) are maintained exactly like MB populations except for the fact that larval culture density of MCU flies is 800 eggs in 1.5 mL of food in a glass vials (25 mm diameter × 90 mm height). Twenty-four such vials are collected every generation per population. Daily, the adults eclosing from these vials are dumped into a Plexiglas cage (24 × 19 × 14 cm) containing a petri plate of cornmeal-charcoal food and wet absorbent cotton for maintaining high RH levels. Nearly 100–110 adults eclose from a crowded vial, which are then transferred into cages. Thus, the adult number is approximately 2500 per population per generation. On day 18 post-egg collection, the flies are provided with a fresh food plate supplemented with *ad libitum* live yeast paste. Two days later, the flies are provided with a fresh food plate and are allowed to oviposit for 18 h. These eggs are then used to start the next generation.

### Bacteria preparation

A gram negative bacteria (*Pseudomonas entomophila*) and a gram positive bacteria (*Enterococcus faecalis*) were used for the infection.

These bacteria are reported to be extracted from *Drosophila melanogaster* and are considered as a natural pathogen for the fruit flies [29, 30]. A day before infections, primary culture was set up by inoculating a small amount of bacterial inoculum from a cryovial into 10 mL of LB medium. The primary culture was grown at 27 degrees Celsius at 150 rpm overnight. After this, a secondary culture was set up by inoculating 10 mL of LB medium with 100 μl of primary culture. Bacteria (OD_600_ = 0.4 ± 0.1) for *Pseudomonas entomophila* and (OD_600_ = 0.8 ± 0.1) for *Enterococcus faecalis* were pelleted down and resuspended in 10 mM MgSO4 to get the same OD. This bacterial suspension was used to infect the flies.

### Infection protocol

Flies were infected in the lateral side of the thorax with the help of a fine needle (minutein pins, 0.1mm, *Fine Science Tools)* dipped in the bacterial suspension [30]. The needle was dipped in bacterial suspension each time the fly was infected. Sham infections for injury control were done in exactly the same way the only difference being 10 mM MgSO_4_ solution was used instead of the bacterial solution.

### Generation of flies for the experiment

To avoid any non-genetic parental effects, both MCU and MB populations were subjected to one generation of same rearing environment, a process referred to as ’standardization’ [31]. For standardisation of flies, 4 bottles of 300 larvae in 50–60 ml of food were grown for every population. Standardized MCU and MB flies housed in separate cages were given a food plate supplemented with ad-libitum live yeast for 48 hours.

Larval crowding, as well as adaptation to crowding, affect development time [32] in *D*. *melanogaster* populations. Therefore, egg collection for different populations and treatments was done on different days, ensuring the age of all flies as adults is comparable on the day of the experiment.

A fresh plate was introduced in cages for 6 hours and eggs laid during this time-window were collected. For the experiment, each replicate of both selected and control populations was subjected to two treatments:

1. The high-density (HD) treatment had 600 eggs per vial containing 2 mL of food.
2. The low-density treatment (LD) had 70 eggs per vial containing 6 mL of food.

Maintenance of experimental flies was same as stock flies, i.e. HD treatment adults were transferred into cages daily with ample food once they eclosed in the culture vials, whereas LD treatments adults were collected into cages on 12^th^-day post egg collection.

On the day of the experiment, four-day-old adults were aspirated out of the cage into sex separated vials. A total of 50 flies per sex per selection regime per treatment were infected with pathogenic bacteria, and equal number of flies were pricked with a needle dipped in a 10 mM sterile solution of MgSO_4_as injury control. Mortality readings were taken every hour for the next 96-hour post-infection. Experiment for each block was performed on a separate day.

### Statistical analyses

We did a survivorship analysis using Cox’s Proportional hazard in R (Version 3.6.2). Flies that were not dead by the end of the observation period were censored. We constructed a Cox model using the "Coxme" [33] package of R. In the model, (a) survivorship post-infection was used as a dependent factor (b) selection regime, density treatment, as independent factors, and (c) block as a random factor. Sexes were analyzed separately in the above model. Analysis of deviance was done on the result of the model to determine the factors affecting survivorship significantly. To plot survival curves, we used the Kaplan-Meier method using the ’Survival’ and "ggusurvplot" [33] packages in R. Since there was negligible mortality in MgSO_4_ pricked control treatment they were not included in the analysis.

## Results

### Survivorship against *Pseudomonas entomophila*

**Fig 1:**
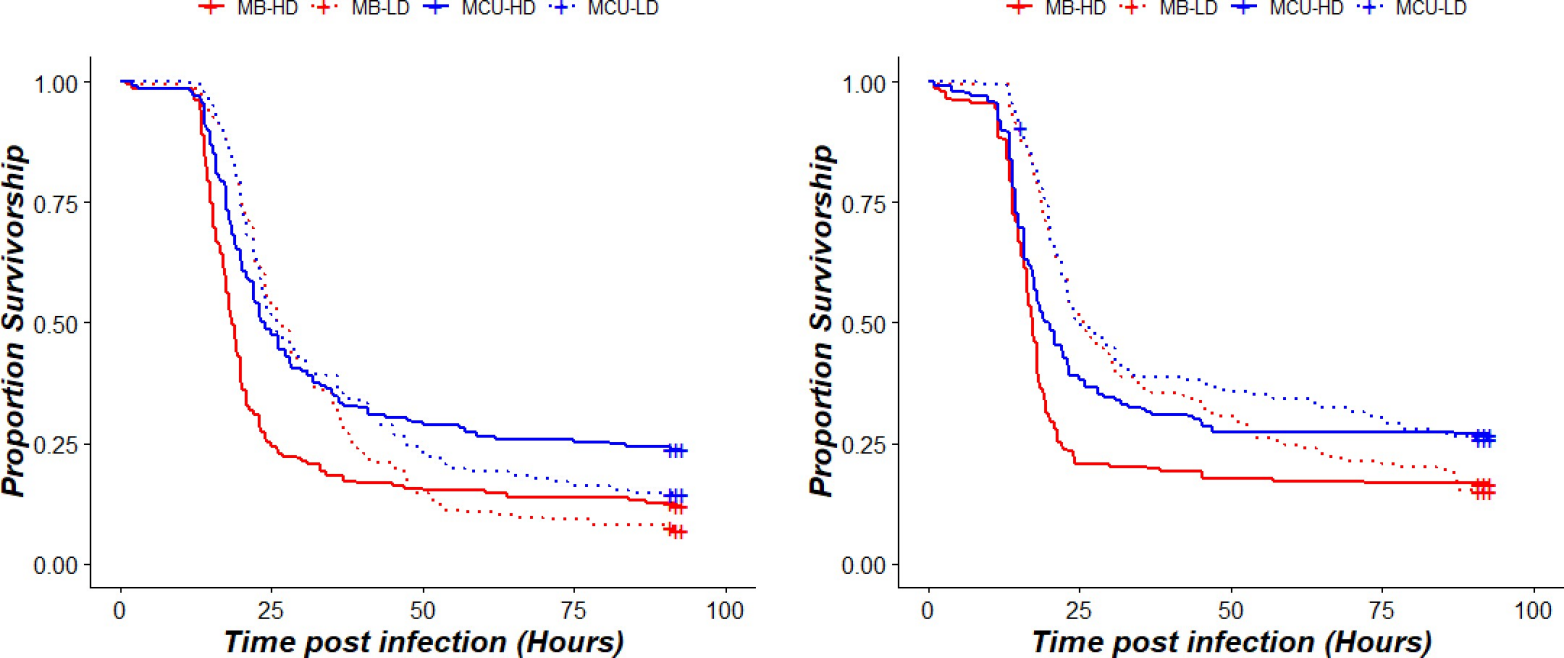
Effect of selection and treatment on post infection survivorship of adults against *Pseudomonas entomophila*: (a) Females (b) Males. Dotted lines represent LD treatments and solid lines represents HD treatments. Red lines are survivorship curves for MBs whereas Blue lines are survivorship curves for MCUs.

MCU populations were better at surviving the infection than MB populations. Cox proportional hazard analysis showed a significant effect of selection and larval-density on survivorship post-infection for both the sexes (Table 1). At the end of the 96-hour post-infection period, more MCU flies were alive as compared to MBs. The median time of survival was also higher for MCUs than MBs (S1 Table). There was a significant interaction between the selection regime and treatment in females but not in males (Fig 1 and Table 1). With greater median time to death, males and females of MB populations from LD treatment survived infection better than HD flies (S1 Table). However, in MCU flies, because of improved survivorship of MCU-HD flies, survivorship of MCU-LD was comparable to MCU-HD (Fig 1 and S1 Table). When blocks were analysed separately, MBs survival was never better than MCUs. However, MBs and MCUs had comparable survivorship in HD treatment of males and LD treatment of females in two out of four blocks (S4–S7 Tables). Block 2 also showed a comparable response of males of low density between MBs and MCUs. In our study, blocks act as an independent replicate of experiments and ancestry, and they show the consistency of evolutionary response. Since all the blocks have been maintained independently for over 240 generations now, the evolution of different correlated traits in different blocks is possible, which might lead to differential adult survivorship post infection across blocks. It is noteworthy that in none of the treatments in both males and females of any block, we found MCUs were worse than MBs in their survivorship post infection. Overall, averaged over all the blocks, both males and females of MCU populations showed significantly better survivorship post-infection than MB flies. We also implemented a logistic regression on flies alive at the end of observation period, the results of which also supports the main takeaway message of the discussed analysis, i.e., there is a significant effect of selection history on survivorship post-infection against *P*. *entomophila*, with MCU flies being better at surviving the infection than MB flies (S8 and S9 Tables). Additionally, overall, in both males and females there was significant effect of larval density treatment with flies from LD treatment having better survivorship than flies from HD treatment (S2(b) Table)

**Table 1:** Summary table of Cox proportional hazards analysis showing effect of selection, density treatment, and their interaction against *Pseudomonas entomophila*.

**Male**	**loglik**	**Chisq**	**df**	**Pr(>|Chi|)**
Null	-3711.4			
Treatment	-3682.6	57.6221	1	**3.18 x 10**^**−14**^
Selection	-3676.4	12.2983	1	**0.000453**
Treatment × Selection	-3675.8	1.2774	1	0.258382
**Female**				
Null	-4062.2			
Treatment	-4058.4	7.5534	1	**0.00599**
Selection	-4046.8	23.2567	1	**1.42 x 10**^**−06**^
Treatment × Selection	-4042.3	8.9215	1	**0.002818**

Significant terms are marked in bold.

### Survivorship against *Enterococcus faecalis*

**Fig 2:**
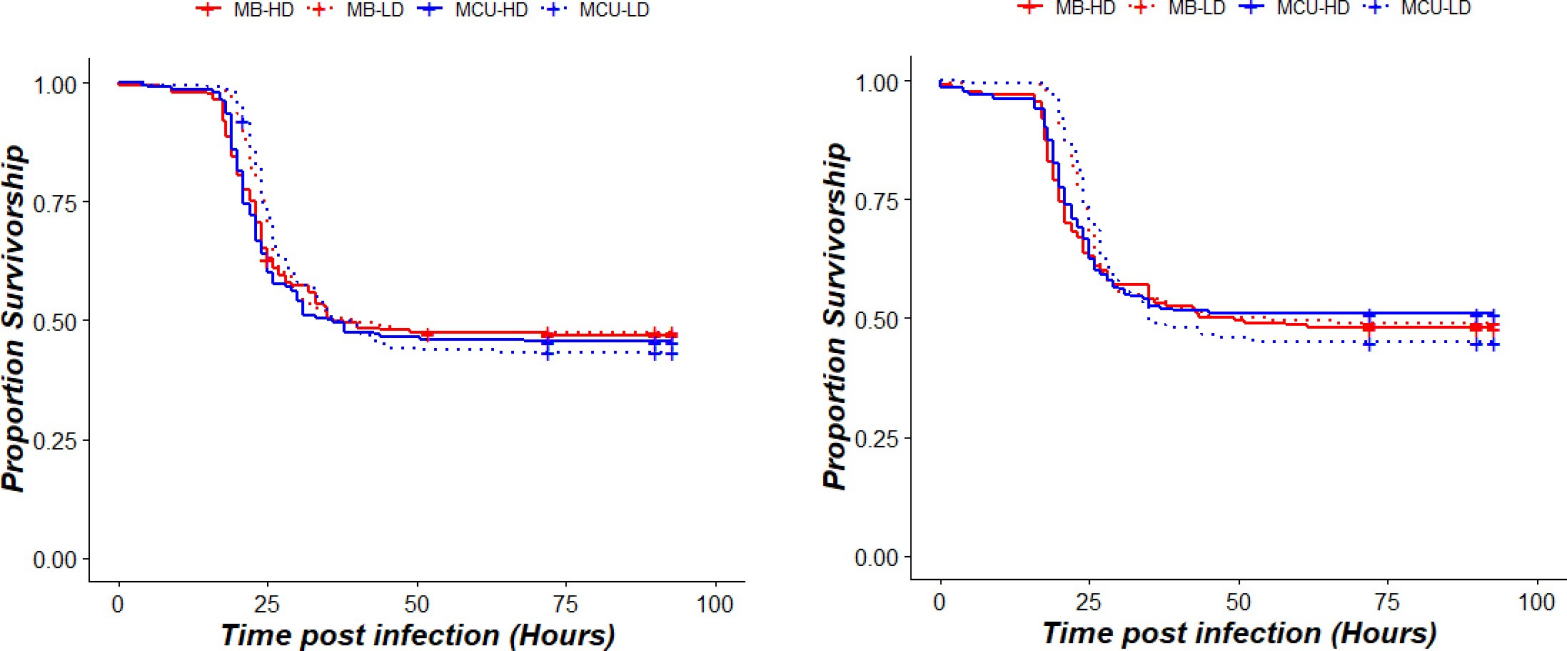
Effect of selection and treatment on post infection survivorship of adults against *Enterococcus faecalis*: (a) Females (b) Males. Dotted lines represent LD treatments and solid lines represents HD treatments. Red lines are survivorship curves for MBs whereas Blue lines are survivorship curves for MCUs.

Sex-specific effect of larval density was observed with males of HD treatments surviving marginally longer and suffering less mortality than males of LD treatment (S2 Table). Moreover, there was no effect of selection history in both male and female on survivorship against *Enterococcus faecalis* (Fig 2 and Table 2). Results were consistent across all the blocks with no effect of selection or larval density treatment in survivorship against *E*. *faecalis*.

**Table 2:** Summary table of Cox proportional hazards analysis showing effect of selection, density treatment, and their interaction against *Enterococcus faecalis*.

Male	loglik	Chisq	df	Pr(>|Chi|)
NULL	-2635.1			
Treatment	-2628.9	12.4704	1	**0.000413**
Selection	-2628.9	0.0012	1	0.972566
Treatment x Selection	-2628.7	0.3182	1	0.572664
**Female**				
NULL	-2746.8			
Treatment	-2745.4	2.9655	1	0.08506
Selection	-2745.2	0.3355	1	0.56246
Treatment x Selection	-2745.2	0.0006	1	0.98024

Significant terms are marked in bold.

## Discussion

In this study, we attempted to answer the following questions:

1. Does larval crowding affect the survivorship post infection in the adult stage?
2. Does adaptation to larval crowding result in the evolution of survivorship post-infection in the adult stage?

We observed an effect of selection history and larval density on adult survivorship post-infection when the flies were infected with gram-negative bacteria, but not when infected gram-positive bacteria.

Previous studies suggest that poor nutritional conditions during larval stages lead to poor adult immune response [34–38] due to lack of resources and poor adult body conditions. The high larval density (HD) treatment used in this experiment leads to significantly smaller adult body size than low larval density (LD) treatments [27]. Since the concentration of bacteria used for infection was the same for both HD and LD treatments, it is possible that HD flies received a higher dose of bacteria per unit body mass than LD flies. Therefore, all else being equal, one might expect lower post infection survival of flies from HD treatment. However, we did not find any such consistent effects of larval growth density on adult post infection survival. In case of *E*. *faecalis* infection, larval density affected survivorship of males from both selected and control populations but not in females. In case of *P*. *entomophila* infection, females and males of selected populations grown under HD treatment had similar survivorship compared to selected females and males from LD treatment. Whereas in control populations, LD flies had better survivorship compared to HD flies. Thus, our results suggest that larval crowding need not necessarily reduce post infection survivorship of adults. The effects of larval crowding on adult survivorship post-infection is likely to depend on the type of pathogen, and the selection history of the host.

We saw an effect of selection on survivorship post infection against *P*. *entomophila* with males and females from the crowding adapted Populations (MCUs) showing significantly better survivorship against *Pseudomonas entomophila* compared to the survivorship of flies from the control populations, suggesting the evolution of survivorship post-infection against a gram-negative pathogen. However, we did not observe the effect of selection history against a gram-positive pathogen. These results suggest that depending on the type of the pathogen, populations adapted to larval crowding can show an evolved improved ability to survive infection.

In *Drosophila*, gram-negative and gram-positive bacteria invoke two different immune response pathways [39, 40]. While the toll pathway is involved in mounting an immune response against gram-positive bacteria, IMD pathway is involved in dealing with immune challenge imposed by gram-negative bacteria [39, 40]. Our results are suggestive of the fact that perhaps there has been a correlated evolution of IMD pathway in MCUs as a result of adaptation to larval crowding environment. Future work involving the investigation of a component of IMD and toll pathways will highlight the proper mechanistic reason responsible for the evolution of pathogen-specific survivorship in these populations.

Effects of developmental environment on the adult immune response have been widely studied in both vertebrate and invertebrate models where the major focus of studies so far has just been on abiotic components of developmental environment like temperature, nutritional availability, etc [34–36, 41, 42]. In larval crowding, both biotic and abiotic components of the environment pose challenges on organisms [32, 43]. Accumulation of toxic waste is abiotic stress as it deteriorates the nutritional value of available food, whereas biotic factor-like intra-specific competition for resources increases the challenges for organisms. Such conditions impose Density-Dependent Selection (DDS) [44, 17]. Theories suggest that due to DDS, different traits are selected across different densities. For example, in *D*. *melanogaster*, evolution at different larval densities lead to the evolution of different life-histories [40]. Evolution of improved pathogen-specific adult survivorship as a result of *adaptation* to larval crowding is a novel addition to our understanding of how DDS acting on one life-stage can shape the traits in other life-stages.

## Supporting information

S1 Table Showing total events (death), median death time for both selected and control populations in males and females against *Pseudomonas entomophila*. HD is low density and LD is high density.
(DOCX)

S2 Table Showing total events (death), median death time for both selected and control populations in males and females against a) *E*. *faecalis b) P*. *entomophila*. HD is low density and LD is high density.
(DOCX)

S3 Table Showing total events (death), median death time for both selected and control populations in males and females against *Enterococcus faecalis*. HD is low density and LD is high density.
(DOCX)

S4 Table Showing total events (death), median death time for both selected and control populations in males and females. HD is low density and LD is high density.
(DOCX)

S5 Table Showing total events (death), median death time for both selected and control populations in males and females. HD is low density and LD is high density.
(DOCX)

S6 Table Showing total events (death), median death time for both selected and control populations in males and females. HD is low density and LD is high density.
(DOCX)

S7 Table Showing total events (death), median death time for both selected and control populations in males and females. HD is low density and LD is high density.
(DOCX)

S8 Table Logistic regression of males alive at the end of the observation period against *P*.*entomophila*. (DOCX)

S9 Table Logistic regression of females alive at the end of the observation period against *P*.*entomophila*. (DOCX)

S10 Table Survivorship data against *Pseudomonas entomophila* bacteria with time of death in hours post infection. (DOCX)

S11 Table Survivorship data against *Enterococcus faecalis* bacteria with time of death in hours post infection. (DOCX)
