# Supplementary material for: Evolution of pathogen-specific improved survivorship post-infection in populations of Drosophila melanogaster adapted to larval crowding
Source: PLoS One. 2021 Apr 14;16(4):e0250055. doi: 10.1371/journal.pone.0250055 (PMC8046209; doi:10.1371/journal.pone.0250055)
Supplement: S1 Table — HD is low density and LD is high density. (DOCX) [file pone.0250055.s001.docx]

| **Selection** | **Treatment** | **Sample size** | **Number of deaths** | **Median** | **0.95 LCL** | **0.95 UCL** |
| --- | --- | --- | --- | --- | --- | --- |
| MB Males | HD | 198 | 165 | 17.3 | 16.3 | 18 |
|  | LD | 184 | 156 | 25.6 | 23 | 30 |
| MCU Males | HD | 198 | 151 | 23.6 | 22 | 28 |
|  | LD | 198 | 169 | 26 | 24 | 30 |
| MB Females | HD | 198 | 174 | 18.5 | 17.5 | 20 |
|  | LD | 197 | 183 | 26 | 24 | 30 |
| MCU Females | HD | 197 | 144 | 20 | 18 | 23 |
|  | LD | 199 | 146 | 25.3 | 23 | 31 |

S1 Table: Showing total events (death), median death time for both selected and control populations in males and females against *Pseudomonas entomophila*. HD is low density and LD is high density
