# Supplementary material for: Evolution of pathogen-specific improved survivorship post-infection in populations of Drosophila melanogaster adapted to larval crowding
Source: PLoS One. 2021 Apr 14;16(4):e0250055. doi: 10.1371/journal.pone.0250055 (PMC8046209; doi:10.1371/journal.pone.0250055)
Supplement: S2 Table — Showing total events (death), median death time for both selected and control populations in males and females against a) E. faecalis b) P. entomophila. HD is low density and LD is high density. (DOCX) [file pone.0250055.s002.docx]

1. *E. faecalis*

| **Treatment** | **Sample size** | **Number of deaths** | **Median** | **0.95 LCL** | **0.95 UCL** |
| --- | --- | --- | --- | --- | --- |
| Treatment=HD | 400 | 202 | 54.5 | 35 | NA |
| Treatment=LD | 400 | 212 | 41 | 33 | NA |
|  |  |  |  |  |  |
| b) *P. entomophila* |  |  |  |  |  |
| Treatment=HD | 791 | 634 | 19 | 18.3 | 20 |
| Treatment=LD | 778 | 654 | 26 | 24.3 | 28 |
|  |  |  |  |  |  |

S2 Table: Showing total events (death), median death time for both selected and control populations in males and females against a) *E. faecalis b) P. entomophila*. HD is low density and LD is high density
