# Supplementary material for: Evolution of pathogen-specific improved survivorship post-infection in populations of Drosophila melanogaster adapted to larval crowding
Source: PLoS One. 2021 Apr 14;16(4):e0250055. doi: 10.1371/journal.pone.0250055 (PMC8046209; doi:10.1371/journal.pone.0250055)
Supplement: S3 Table — HD is low density and LD is high density. (DOCX) [file pone.0250055.s003.docx]

| **Selection** | **Treatment** | **Sample size** | **Number of deaths** | **Median** | **0.95 LCL** | **0.95 UCL** |
| --- | --- | --- | --- | --- | --- | --- |
| MB Males | HD | 200 | 104 | 46.5 | 35 | NA |
|  | LD | 200 | 102 | 50.5 | 29 | NA |
| MCU Males | HD | 200 | 98 | NA | 30 | NA |
|  | LD | 200 | 110 | 35 | 30 | NA |
| MB Females | HD | 200 | 106 | 36 | 32 | NA |
|  | LD | 200 | 105 | 39.5 | 30 | NA |
| MCU Females | HD | 200 | 109 | 35.5 | 29 | NA |
|  | LD | 200 | 113 | 37 | 33 | NA |

S3 Table: Showing total events (death), median death time for both selected and control populations in males and females against *Enterococcus faecalis*. HD is low density and LD is high density
