# Supplementary material for: Evolution of pathogen-specific improved survivorship post-infection in populations of Drosophila melanogaster adapted to larval crowding
Source: PLoS One. 2021 Apr 14;16(4):e0250055. doi: 10.1371/journal.pone.0250055 (PMC8046209; doi:10.1371/journal.pone.0250055)
Supplement: S4 Table — HD is low density and LD is high density. (DOCX) [file pone.0250055.s004.docx]

*Block 1 Female*

*Block 1 Male*

S4 Table: Showing total events (death), median death time for both selected and control populations in males and females. HD is low density and LD is high density

|  |  | n | events | median | 0.95LCL | 0.95UCL |
| --- | --- | --- | --- | --- | --- | --- |
| SELECTION=MCU, | TREATMENT=HD | 50 | 46 | 14.5 | 13.5 | 16.5 |
| SELECTION=MCU, | TREATMENT=LD | 50 | 34 | 23 | 20 | 65 |
| SELECTION=MB, | TREATMENT=HD | 50 | 45 | 15 | 13.5 | 16.5 |
| SELECTION=MB, | TREATMENT=LD | 50 | 41 | 18.5 | 17.5 | 25 |

|  |  | n | events | median | 0.95LCL | 0.95UCL |
| --- | --- | --- | --- | --- | --- | --- |
| SELECTION=MCU, | TREATMENT=HD | 50 | 36 | 18.5 | 17.5 | 62 |
| SELECTION=MCU, | TREATMENT=LD | 50 | 48 | 23.5 | 22 | 43 |
| SELECTION=MB, | TREATMENT=HD | 50 | 47 | 16 | 14.5 | 17.5 |
| SELECTION=MB, | TREATMENT=LD | 50 | 47 | 22.5 | 21 | 32 |
