# Supplementary material for: Evolution of pathogen-specific improved survivorship post-infection in populations of Drosophila melanogaster adapted to larval crowding
Source: PLoS One. 2021 Apr 14;16(4):e0250055. doi: 10.1371/journal.pone.0250055 (PMC8046209; doi:10.1371/journal.pone.0250055)
Supplement: S5 Table — HD is low density and LD is high density. (DOCX) [file pone.0250055.s005.docx]

|  |  | n | events | median | 0.95LCL | 0.95UCL |
| --- | --- | --- | --- | --- | --- | --- |
| SELECTION=MCU, | TREATMENT=HD | 49 | 26 | 45.3 | 28 | NA |
| SELECTION=MCU, | TREATMENT=LD | 51 | 38 | 25.3 | 21.3 | 34 |
| SELECTION=MB, | TREATMENT=HD | 50 | 44 | 19.3 | 17.3 | 21.3 |
| SELECTION=MB, | TREATMENT=LD | 36 | 35 | 31.5 | 27.3 | 45.3 |

|  |  | n | events | median | 0.95LCL | 0.95UCL |
| --- | --- | --- | --- | --- | --- | --- |
| SELECTION=MCU, | TREATMENT=HD | 50 | 39 | 27.3 | 26.3 | 36.3 |
| SELECTION=MCU, | TREATMENT=LD | 50 | 38 | 24.8 | 23.3 | 34 |
| SELECTION=MB, | TREATMENT=HD | 50 | 40 | 19.3 | 17.3 | 23.3 |
| SELECTION=MB, | TREATMENT=LD | 49 | 45 | 24.3 | 22.3 | 31 |

Block 2 Males

Block 2 Females

S5 Table: Showing total events (death), median death time for both selected and control populations in males and females. HD is low density and LD is high density
