# Supplementary material for: Evolution of pathogen-specific improved survivorship post-infection in populations of Drosophila melanogaster adapted to larval crowding
Source: PLoS One. 2021 Apr 14;16(4):e0250055. doi: 10.1371/journal.pone.0250055 (PMC8046209; doi:10.1371/journal.pone.0250055)
Supplement: S6 Table — HD is low density and LD is high density. (DOCX) [file pone.0250055.s006.docx]

Block 3 Male

Block 3 Female

S6 Table: Showing total events (death), median death time for both selected and control populations in males and females. HD is low density and LD is high density

|  |  | n | events | median | 0.95LCL | 0.95UCL |
| --- | --- | --- | --- | --- | --- | --- |
| SELECTION=MCU, | TREATMENT=HD | 50 | 31 | 27.5 | 19 | NA |
| SELECTION=MCU, | TREATMENT=LD | 50 | 36 | 40 | 22 | 84 |
| SELECTION=MB, | TREATMENT=HD | 50 | 36 | 18 | 15 | 18 |
| SELECTION=MB, | TREATMENT=LD | 50 | 37 | 34 | 24 | 59 |

|  |  | n | events | median | 0.95LCL | 0.95UCL |
| --- | --- | --- | --- | --- | --- | --- |
| SELECTION=MCU, | TREATMENT=HD | 50 | 39 | 22 | 19 | 28 |
| SELECTION=MCU, | TREATMENT=LD | 50 | 38 | 33 | 23 | 47 |
| SELECTION=MB, | TREATMENT=HD | 50 | 42 | 21 | 19 | 33 |
| SELECTION=MB, | TREATMENT=LD | 50 | 48 | 31 | 27 | 39 |
