# Supplementary material for: Evolution of pathogen-specific improved survivorship post-infection in populations of Drosophila melanogaster adapted to larval crowding
Source: PLoS One. 2021 Apr 14;16(4):e0250055. doi: 10.1371/journal.pone.0250055 (PMC8046209; doi:10.1371/journal.pone.0250055)
Supplement: S7 Table — HD is low density and LD is high density. (DOCX) [file pone.0250055.s007.docx]

|  |  | n | events | median | 0.95LCL | 0.95UCL |
| --- | --- | --- | --- | --- | --- | --- |
| SELECTION=MCU, | TREATMENT=HD | 48 | 41 | 17.5 | 16 | 22 |
| SELECTION=MCU, | TREATMENT=LD | 48 | 38 | 25.5 | 22 | 34 |
| SELECTION=MB, | TREATMENT=HD | 48 | 40 | 17 | 15 | 21 |
| SELECTION=MB, | TREATMENT=LD | 48 | 43 | 23 | 22 | 30 |

|  |  | n | events | median | 0.95LCL | 0.95UCL |
| --- | --- | --- | --- | --- | --- | --- |
| SELECTION=MCU, | TREATMENT=HD | 48 | 37 | 29.5 | 23 | 41 |
| SELECTION=MCU, | TREATMENT=LD | 48 | 45 | 27 | 24 | 36 |
| SELECTION=MB, | TREATMENT=HD | 48 | 45 | 19 | 18 | 20 |
| SELECTION=MB, | TREATMENT=LD | 48 | 43 | 26 | 24 | 34 |

Block 4 Males

Block 4 Females

S7 Table: Showing total events (death), median death time for both selected and control populations in males and females. HD is low density and LD is high density
