# Supplementary material for: Evolution of pathogen-specific improved survivorship post-infection in populations of Drosophila melanogaster adapted to larval crowding
Source: PLoS One. 2021 Apr 14;16(4):e0250055. doi: 10.1371/journal.pone.0250055 (PMC8046209; doi:10.1371/journal.pone.0250055)
Supplement: S8 Table — (DOCX) [file pone.0250055.s008.docx]

|  | Estimate | Std. Error | z value | Pr(>\|z\|) |
| --- | --- | --- | --- | --- |
| (Intercept) | 1.64047 | 0.24178 | 6.785 | 1.16E-11 |
| SelectionMCU | -0.61922 | 0.25089 | -2.468 | **0.0136** |
| TreatmentLD | 0.10592 | 0.28164 | 0.376 | 0.7068 |
| SelectionMCU:TreatmentLD | -0.09135 | 0.36285 | -0.252 | 0.8012 |

S8 Table: logistic regression of males alive at the end of the observation period against *P.entomophila*
