# Supplementary material for: Evolution of pathogen-specific improved survivorship post-infection in populations of Drosophila melanogaster adapted to larval crowding
Source: PLoS One. 2021 Apr 14;16(4):e0250055. doi: 10.1371/journal.pone.0250055 (PMC8046209; doi:10.1371/journal.pone.0250055)
Supplement: S9 Table — (DOCX) [file pone.0250055.s009.docx]

|  | Estimate | Std. Error | z value | Pr(>\|z\|) |
| --- | --- | --- | --- | --- |
| (Intercept) | 1.993907 | 0.235964 | 8.45 | < 2e-16 |
| SelectionMCU | -0.81752 | 0.274827 | -2.975 | **0.00293** |
| TreatmentLD | 0.590203 | 0.352652 | 1.674 | 0.09421 |
| SelectionMCU:TreatmentLD | 0.008111 | 0.439173 | 0.018 | 0.98527 |

S9 Table: logistic regression of females alive at the end of the observation period against *P.entomophila*
