# Supplementary material for: Evolution of pathogen-specific improved survivorship post-infection in populations of Drosophila melanogaster adapted to larval crowding
Source: PLoS One. 2021 Apr 14;16(4):e0250055. doi: 10.1371/journal.pone.0250055 (PMC8046209; doi:10.1371/journal.pone.0250055)
Supplement: S10 Table — (DOCX) [file pone.0250055.s010.docx]

**Raw Data Files**

**S10 Table: Survivorship data against *Pseudomonas entomophila* bacteria with time of death in hours post infection**

| Block | Selection | Treatment | Sex | Time | Censor |
| --- | --- | --- | --- | --- | --- |
| 1 | MCU | HD | F | 2 | 1 |
| 1 | MCU | HD | F | 2 | 1 |
| 1 | MCU | HD | F | 11.5 | 1 |
| 1 | MCU | HD | F | 12 | 1 |
| 1 | MCU | HD | F | 13.5 | 1 |
| 1 | MCU | HD | F | 13.5 | 1 |
| 1 | MCU | HD | F | 14.5 | 1 |
| 1 | MCU | HD | F | 15.5 | 1 |
| 1 | MCU | HD | F | 15.5 | 1 |
| 1 | MCU | HD | F | 15.5 | 1 |
| 1 | MCU | HD | F | 15.5 | 1 |
| 1 | MCU | HD | F | 16.5 | 1 |
| 1 | MCU | HD | F | 17.5 | 1 |
| 1 | MCU | HD | F | 17.5 | 1 |
| 1 | MCU | HD | F | 17.5 | 1 |
| 1 | MCU | HD | F | 17.5 | 1 |
| 1 | MCU | HD | F | 17.5 | 1 |
| 1 | MCU | HD | F | 17.5 | 1 |
| 1 | MCU | HD | F | 17.5 | 1 |
| 1 | MCU | HD | F | 17.5 | 1 |
| 1 | MCU | HD | F | 17.5 | 1 |
| 1 | MCU | HD | F | 17.5 | 1 |
| 1 | MCU | HD | F | 18.5 | 1 |
| 1 | MCU | HD | F | 18.5 | 1 |
| 1 | MCU | HD | F | 18.5 | 1 |
| 1 | MCU | HD | F | 18.5 | 1 |
| 1 | MCU | HD | F | 20 | 1 |
| 1 | MCU | HD | F | 22 | 1 |
| 1 | MCU | HD | F | 22 | 1 |
| 1 | MCU | HD | F | 23 | 1 |
| 1 | MCU | HD | F | 25 | 1 |
| 1 | MCU | HD | F | 30 | 1 |
| 1 | MCU | HD | F | 47.5 | 1 |
| 1 | MCU | HD | F | 62 | 1 |
| 1 | MCU | HD | F | 83.5 | 1 |
| 1 | MCU | HD | F | 91 | 1 |
| 1 | MCU | HD | F | 91 | 0 |
| 1 | MCU | HD | F | 91 | 0 |
| 1 | MCU | HD | F | 91 | 0 |
| 1 | MCU | HD | F | 91 | 0 |
| 1 | MCU | HD | F | 91 | 0 |
| 1 | MCU | HD | F | 91 | 0 |
| 1 | MCU | HD | F | 91 | 0 |
| 1 | MCU | HD | F | 91 | 0 |
| 1 | MCU | HD | F | 91 | 0 |
| 1 | MCU | HD | F | 91 | 0 |
| 1 | MCU | HD | F | 91 | 0 |
| 1 | MCU | HD | F | 91 | 0 |
| 1 | MCU | HD | F | 91 | 0 |
| 1 | MCU | HD | F | 91 | 0 |
| 1 | MCU | LD | F | 11.5 | 1 |
| 1 | MCU | LD | F | 13.5 | 1 |
| 1 | MCU | LD | F | 13.5 | 1 |
| 1 | MCU | LD | F | 13.5 | 1 |
| 1 | MCU | LD | F | 14.5 | 1 |
| 1 | MCU | LD | F | 16.5 | 1 |
| 1 | MCU | LD | F | 17.5 | 1 |
| 1 | MCU | LD | F | 18.5 | 1 |
| 1 | MCU | LD | F | 18.5 | 1 |
| 1 | MCU | LD | F | 18.5 | 1 |
| 1 | MCU | LD | F | 18.5 | 1 |
| 1 | MCU | LD | F | 19.5 | 1 |
| 1 | MCU | LD | F | 19.5 | 1 |
| 1 | MCU | LD | F | 21 | 1 |
| 1 | MCU | LD | F | 21 | 1 |
| 1 | MCU | LD | F | 21 | 1 |
| 1 | MCU | LD | F | 21 | 1 |
| 1 | MCU | LD | F | 22 | 1 |
| 1 | MCU | LD | F | 22 | 1 |
| 1 | MCU | LD | F | 22 | 1 |
| 1 | MCU | LD | F | 22 | 1 |
| 1 | MCU | LD | F | 22 | 1 |
| 1 | MCU | LD | F | 22 | 1 |
| 1 | MCU | LD | F | 23 | 1 |
| 1 | MCU | LD | F | 23 | 1 |
| 1 | MCU | LD | F | 24 | 1 |
| 1 | MCU | LD | F | 26 | 1 |
| 1 | MCU | LD | F | 26 | 1 |
| 1 | MCU | LD | F | 26 | 1 |
| 1 | MCU | LD | F | 28 | 1 |
| 1 | MCU | LD | F | 28 | 1 |
| 1 | MCU | LD | F | 36 | 1 |
| 1 | MCU | LD | F | 36 | 1 |
| 1 | MCU | LD | F | 43 | 1 |
| 1 | MCU | LD | F | 43 | 1 |
| 1 | MCU | LD | F | 45 | 1 |
| 1 | MCU | LD | F | 45 | 1 |
| 1 | MCU | LD | F | 47.5 | 1 |
| 1 | MCU | LD | F | 49.5 | 1 |
| 1 | MCU | LD | F | 50 | 1 |
| 1 | MCU | LD | F | 51 | 1 |
| 1 | MCU | LD | F | 54 | 1 |
| 1 | MCU | LD | F | 65 | 1 |
| 1 | MCU | LD | F | 68 | 1 |
| 1 | MCU | LD | F | 71 | 1 |
| 1 | MCU | LD | F | 74 | 1 |
| 1 | MCU | LD | F | 81 | 1 |
| 1 | MCU | LD | F | 81 | 1 |
| 1 | MCU | LD | F | 91 | 0 |
| 1 | MCU | LD | F | 91 | 0 |
| 2 | MCU | LD | F | 15.3 | 1 |
| 2 | MCU | LD | F | 16.3 | 1 |
| 2 | MCU | LD | F | 16.3 | 1 |
| 2 | MCU | LD | F | 17.3 | 1 |
| 2 | MCU | LD | F | 17.3 | 1 |
| 2 | MCU | LD | F | 17.3 | 1 |
| 2 | MCU | LD | F | 18.3 | 1 |
| 2 | MCU | LD | F | 19.3 | 1 |
| 2 | MCU | LD | F | 19.3 | 1 |
| 2 | MCU | LD | F | 19.3 | 1 |
| 2 | MCU | LD | F | 20.3 | 1 |
| 2 | MCU | LD | F | 20.3 | 1 |
| 2 | MCU | LD | F | 20.3 | 1 |
| 2 | MCU | LD | F | 20.3 | 1 |
| 2 | MCU | LD | F | 21.3 | 1 |
| 2 | MCU | LD | F | 21.3 | 1 |
| 2 | MCU | LD | F | 22.3 | 1 |
| 2 | MCU | LD | F | 22.3 | 1 |
| 2 | MCU | LD | F | 23.3 | 1 |
| 2 | MCU | LD | F | 23.3 | 1 |
| 2 | MCU | LD | F | 23.3 | 1 |
| 2 | MCU | LD | F | 23.3 | 1 |
| 2 | MCU | LD | F | 24.3 | 1 |
| 2 | MCU | LD | F | 24.3 | 1 |
| 2 | MCU | LD | F | 24.3 | 1 |
| 2 | MCU | LD | F | 25.3 | 1 |
| 2 | MCU | LD | F | 25.3 | 1 |
| 2 | MCU | LD | F | 27.3 | 1 |
| 2 | MCU | LD | F | 29.3 | 1 |
| 2 | MCU | LD | F | 30 | 1 |
| 2 | MCU | LD | F | 31 | 1 |
| 2 | MCU | LD | F | 31 | 1 |
| 2 | MCU | LD | F | 31 | 1 |
| 2 | MCU | LD | F | 34 | 1 |
| 2 | MCU | LD | F | 40.3 | 1 |
| 2 | MCU | LD | F | 45.3 | 1 |
| 2 | MCU | LD | F | 57 | 1 |
| 2 | MCU | LD | F | 64.3 | 1 |
| 2 | MCU | LD | F | 92 | 0 |
| 2 | MCU | LD | F | 92 | 0 |
| 2 | MCU | LD | F | 92 | 0 |
| 2 | MCU | LD | F | 92 | 0 |
| 2 | MCU | LD | F | 92 | 0 |
| 2 | MCU | LD | F | 92 | 0 |
| 2 | MCU | LD | F | 92 | 0 |
| 2 | MCU | LD | F | 92 | 0 |
| 2 | MCU | LD | F | 92 | 0 |
| 2 | MCU | LD | F | 92 | 0 |
| 2 | MCU | LD | F | 92 | 0 |
| 2 | MCU | LD | F | 92 | 0 |
| 2 | MCU | HD | F | 13.3 | 1 |
| 2 | MCU | HD | F | 14.3 | 1 |
| 2 | MCU | HD | F | 16.3 | 1 |
| 2 | MCU | HD | F | 16.3 | 1 |
| 2 | MCU | HD | F | 17.3 | 1 |
| 2 | MCU | HD | F | 17.3 | 1 |
| 2 | MCU | HD | F | 18.3 | 1 |
| 2 | MCU | HD | F | 18.3 | 1 |
| 2 | MCU | HD | F | 19.3 | 1 |
| 2 | MCU | HD | F | 19.3 | 1 |
| 2 | MCU | HD | F | 20.3 | 1 |
| 2 | MCU | HD | F | 20.3 | 1 |
| 2 | MCU | HD | F | 20.3 | 1 |
| 2 | MCU | HD | F | 20.3 | 1 |
| 2 | MCU | HD | F | 21.3 | 1 |
| 2 | MCU | HD | F | 22.3 | 1 |
| 2 | MCU | HD | F | 23.3 | 1 |
| 2 | MCU | HD | F | 23.3 | 1 |
| 2 | MCU | HD | F | 26.3 | 1 |
| 2 | MCU | HD | F | 26.3 | 1 |
| 2 | MCU | HD | F | 26.3 | 1 |
| 2 | MCU | HD | F | 26.3 | 1 |
| 2 | MCU | HD | F | 26.3 | 1 |
| 2 | MCU | HD | F | 26.3 | 1 |
| 2 | MCU | HD | F | 27.3 | 1 |
| 2 | MCU | HD | F | 27.3 | 1 |
| 2 | MCU | HD | F | 27.3 | 1 |
| 2 | MCU | HD | F | 28.3 | 1 |
| 2 | MCU | HD | F | 28.3 | 1 |
| 2 | MCU | HD | F | 31 | 1 |
| 2 | MCU | HD | F | 33 | 1 |
| 2 | MCU | HD | F | 35.3 | 1 |
| 2 | MCU | HD | F | 36.3 | 1 |
| 2 | MCU | HD | F | 36.3 | 1 |
| 2 | MCU | HD | F | 39.3 | 1 |
| 2 | MCU | HD | F | 41.3 | 1 |
| 2 | MCU | HD | F | 48.3 | 1 |
| 2 | MCU | HD | F | 57 | 1 |
| 2 | MCU | HD | F | 57 | 1 |
| 2 | MCU | HD | F | 92 | 0 |
| 2 | MCU | HD | F | 92 | 0 |
| 2 | MCU | HD | F | 92 | 0 |
| 2 | MCU | HD | F | 92 | 0 |
| 2 | MCU | HD | F | 92 | 0 |
| 2 | MCU | HD | F | 92 | 0 |
| 2 | MCU | HD | F | 92 | 0 |
| 2 | MCU | HD | F | 92 | 0 |
| 2 | MCU | HD | F | 92 | 0 |
| 2 | MCU | HD | F | 92 | 0 |
| 2 | MCU | HD | F | 92 | 0 |
| 3 | MCU | HD | F | 3 | 1 |
| 3 | MCU | HD | F | 12 | 1 |
| 3 | MCU | HD | F | 14 | 1 |
| 3 | MCU | HD | F | 14 | 1 |
| 3 | MCU | HD | F | 14 | 1 |
| 3 | MCU | HD | F | 14 | 1 |
| 3 | MCU | HD | F | 14 | 1 |
| 3 | MCU | HD | F | 15 | 1 |
| 3 | MCU | HD | F | 15 | 1 |
| 3 | MCU | HD | F | 15 | 1 |
| 3 | MCU | HD | F | 16 | 1 |
| 3 | MCU | HD | F | 16 | 1 |
| 3 | MCU | HD | F | 16 | 1 |
| 3 | MCU | HD | F | 16 | 1 |
| 3 | MCU | HD | F | 16 | 1 |
| 3 | MCU | HD | F | 18 | 1 |
| 3 | MCU | HD | F | 18 | 1 |
| 3 | MCU | HD | F | 18 | 1 |
| 3 | MCU | HD | F | 19 | 1 |
| 3 | MCU | HD | F | 19 | 1 |
| 3 | MCU | HD | F | 20 | 1 |
| 3 | MCU | HD | F | 21 | 1 |
| 3 | MCU | HD | F | 21 | 1 |
| 3 | MCU | HD | F | 22 | 1 |
| 3 | MCU | HD | F | 22 | 1 |
| 3 | MCU | HD | F | 22 | 1 |
| 3 | MCU | HD | F | 22 | 1 |
| 3 | MCU | HD | F | 22 | 1 |
| 3 | MCU | HD | F | 23 | 1 |
| 3 | MCU | HD | F | 23 | 1 |
| 3 | MCU | HD | F | 24 | 1 |
| 3 | MCU | HD | F | 24 | 1 |
| 3 | MCU | HD | F | 25 | 1 |
| 3 | MCU | HD | F | 28 | 1 |
| 3 | MCU | HD | F | 34 | 1 |
| 3 | MCU | HD | F | 37 | 1 |
| 3 | MCU | HD | F | 59 | 1 |
| 3 | MCU | HD | F | 59 | 1 |
| 3 | MCU | HD | F | 81 | 1 |
| 3 | MCU | HD | F | 93 | 0 |
| 3 | MCU | HD | F | 93 | 0 |
| 3 | MCU | HD | F | 93 | 0 |
| 3 | MCU | HD | F | 93 | 0 |
| 3 | MCU | HD | F | 93 | 0 |
| 3 | MCU | HD | F | 93 | 0 |
| 3 | MCU | HD | F | 93 | 0 |
| 3 | MCU | HD | F | 93 | 0 |
| 3 | MCU | HD | F | 93 | 0 |
| 3 | MCU | HD | F | 93 | 0 |
| 3 | MCU | HD | F | 93 | 0 |
| 3 | MCU | LD | F | 14 | 1 |
| 3 | MCU | LD | F | 14 | 1 |
| 3 | MCU | LD | F | 16 | 1 |
| 3 | MCU | LD | F | 16 | 1 |
| 3 | MCU | LD | F | 16 | 1 |
| 3 | MCU | LD | F | 17 | 1 |
| 3 | MCU | LD | F | 17 | 1 |
| 3 | MCU | LD | F | 17 | 1 |
| 3 | MCU | LD | F | 18 | 1 |
| 3 | MCU | LD | F | 18 | 1 |
| 3 | MCU | LD | F | 19 | 1 |
| 3 | MCU | LD | F | 19 | 1 |
| 3 | MCU | LD | F | 19 | 1 |
| 3 | MCU | LD | F | 20 | 1 |
| 3 | MCU | LD | F | 20 | 1 |
| 3 | MCU | LD | F | 20 | 1 |
| 3 | MCU | LD | F | 21 | 1 |
| 3 | MCU | LD | F | 21 | 1 |
| 3 | MCU | LD | F | 23 | 1 |
| 3 | MCU | LD | F | 23 | 1 |
| 3 | MCU | LD | F | 23 | 1 |
| 3 | MCU | LD | F | 24 | 1 |
| 3 | MCU | LD | F | 24 | 1 |
| 3 | MCU | LD | F | 24 | 1 |
| 3 | MCU | LD | F | 30 | 1 |
| 3 | MCU | LD | F | 36 | 1 |
| 3 | MCU | LD | F | 36 | 1 |
| 3 | MCU | LD | F | 36 | 1 |
| 3 | MCU | LD | F | 36 | 1 |
| 3 | MCU | LD | F | 41 | 1 |
| 3 | MCU | LD | F | 43 | 1 |
| 3 | MCU | LD | F | 43 | 1 |
| 3 | MCU | LD | F | 45 | 1 |
| 3 | MCU | LD | F | 47 | 1 |
| 3 | MCU | LD | F | 47 | 1 |
| 3 | MCU | LD | F | 54 | 1 |
| 3 | MCU | LD | F | 75 | 1 |
| 3 | MCU | LD | F | 84 | 1 |
| 3 | MCU | LD | F | 93 | 0 |
| 3 | MCU | LD | F | 93 | 0 |
| 3 | MCU | LD | F | 93 | 0 |
| 3 | MCU | LD | F | 93 | 0 |
| 3 | MCU | LD | F | 93 | 0 |
| 3 | MCU | LD | F | 93 | 0 |
| 3 | MCU | LD | F | 93 | 0 |
| 3 | MCU | LD | F | 93 | 0 |
| 3 | MCU | LD | F | 93 | 0 |
| 3 | MCU | LD | F | 93 | 0 |
| 3 | MCU | LD | F | 93 | 0 |
| 3 | MCU | LD | F | 93 | 0 |
| 4 | MCU | HD | F | 14 | 1 |
| 4 | MCU | HD | F | 14 | 1 |
| 4 | MCU | HD | F | 14 | 1 |
| 4 | MCU | HD | F | 14 | 1 |
| 4 | MCU | HD | F | 15 | 1 |
| 4 | MCU | HD | F | 15 | 1 |
| 4 | MCU | HD | F | 15 | 1 |
| 4 | MCU | HD | F | 16 | 1 |
| 4 | MCU | HD | F | 16 | 1 |
| 4 | MCU | HD | F | 16 | 1 |
| 4 | MCU | HD | F | 18 | 1 |
| 4 | MCU | HD | F | 19 | 1 |
| 4 | MCU | HD | F | 19 | 1 |
| 4 | MCU | HD | F | 20 | 1 |
| 4 | MCU | HD | F | 20 | 1 |
| 4 | MCU | HD | F | 20 | 1 |
| 4 | MCU | HD | F | 21 | 1 |
| 4 | MCU | HD | F | 22 | 1 |
| 4 | MCU | HD | F | 23 | 1 |
| 4 | MCU | HD | F | 23 | 1 |
| 4 | MCU | HD | F | 23 | 1 |
| 4 | MCU | HD | F | 24 | 1 |
| 4 | MCU | HD | F | 28 | 1 |
| 4 | MCU | HD | F | 28 | 1 |
| 4 | MCU | HD | F | 31 | 1 |
| 4 | MCU | HD | F | 32 | 1 |
| 4 | MCU | HD | F | 32 | 1 |
| 4 | MCU | HD | F | 32 | 1 |
| 4 | MCU | HD | F | 35 | 1 |
| 4 | MCU | HD | F | 35 | 1 |
| 4 | MCU | HD | F | 36 | 1 |
| 4 | MCU | HD | F | 41 | 1 |
| 4 | MCU | HD | F | 41 | 1 |
| 4 | MCU | HD | F | 44 | 1 |
| 4 | MCU | HD | F | 50 | 1 |
| 4 | MCU | HD | F | 56 | 1 |
| 4 | MCU | HD | F | 75 | 1 |
| 4 | MCU | HD | F | 92 | 0 |
| 4 | MCU | HD | F | 92 | 0 |
| 4 | MCU | HD | F | 92 | 0 |
| 4 | MCU | HD | F | 92 | 0 |
| 4 | MCU | HD | F | 92 | 0 |
| 4 | MCU | HD | F | 92 | 0 |
| 4 | MCU | HD | F | 92 | 0 |
| 4 | MCU | HD | F | 92 | 0 |
| 4 | MCU | HD | F | 92 | 0 |
| 4 | MCU | HD | F | 92 | 0 |
| 4 | MCU | HD | F | 92 | 0 |
| 4 | MCU | LD | F | 15 | 1 |
| 4 | MCU | LD | F | 15 | 1 |
| 4 | MCU | LD | F | 16 | 1 |
| 4 | MCU | LD | F | 16 | 1 |
| 4 | MCU | LD | F | 18 | 1 |
| 4 | MCU | LD | F | 20 | 1 |
| 4 | MCU | LD | F | 20 | 1 |
| 4 | MCU | LD | F | 20 | 1 |
| 4 | MCU | LD | F | 20 | 1 |
| 4 | MCU | LD | F | 20 | 1 |
| 4 | MCU | LD | F | 20 | 1 |
| 4 | MCU | LD | F | 20 | 1 |
| 4 | MCU | LD | F | 21 | 1 |
| 4 | MCU | LD | F | 21 | 1 |
| 4 | MCU | LD | F | 23 | 1 |
| 4 | MCU | LD | F | 23 | 1 |
| 4 | MCU | LD | F | 23 | 1 |
| 4 | MCU | LD | F | 24 | 1 |
| 4 | MCU | LD | F | 24 | 1 |
| 4 | MCU | LD | F | 25 | 1 |
| 4 | MCU | LD | F | 25 | 1 |
| 4 | MCU | LD | F | 26 | 1 |
| 4 | MCU | LD | F | 26 | 1 |
| 4 | MCU | LD | F | 27 | 1 |
| 4 | MCU | LD | F | 27 | 1 |
| 4 | MCU | LD | F | 27 | 1 |
| 4 | MCU | LD | F | 28 | 1 |
| 4 | MCU | LD | F | 29 | 1 |
| 4 | MCU | LD | F | 29 | 1 |
| 4 | MCU | LD | F | 31 | 1 |
| 4 | MCU | LD | F | 32 | 1 |
| 4 | MCU | LD | F | 36 | 1 |
| 4 | MCU | LD | F | 37 | 1 |
| 4 | MCU | LD | F | 38 | 1 |
| 4 | MCU | LD | F | 38 | 1 |
| 4 | MCU | LD | F | 41 | 1 |
| 4 | MCU | LD | F | 41 | 1 |
| 4 | MCU | LD | F | 41 | 1 |
| 4 | MCU | LD | F | 41 | 1 |
| 4 | MCU | LD | F | 47 | 1 |
| 4 | MCU | LD | F | 47 | 1 |
| 4 | MCU | LD | F | 50 | 1 |
| 4 | MCU | LD | F | 53 | 1 |
| 4 | MCU | LD | F | 53 | 1 |
| 4 | MCU | LD | F | 53 | 1 |
| 4 | MCU | LD | F | 92 | 0 |
| 4 | MCU | LD | F | 92 | 0 |
| 4 | MCU | LD | F | 92 | 0 |
| 1 | MCU | HD | M | 4 | 1 |
| 1 | MCU | HD | M | 10 | 1 |
| 1 | MCU | HD | M | 11.5 | 1 |
| 1 | MCU | HD | M | 11.5 | 1 |
| 1 | MCU | HD | M | 11.5 | 1 |
| 1 | MCU | HD | M | 11.5 | 1 |
| 1 | MCU | HD | M | 11.5 | 1 |
| 1 | MCU | HD | M | 11.5 | 1 |
| 1 | MCU | HD | M | 11.5 | 1 |
| 1 | MCU | HD | M | 12 | 1 |
| 1 | MCU | HD | M | 12 | 1 |
| 1 | MCU | HD | M | 12 | 1 |
| 1 | MCU | HD | M | 13.5 | 1 |
| 1 | MCU | HD | M | 13.5 | 1 |
| 1 | MCU | HD | M | 13.5 | 1 |
| 1 | MCU | HD | M | 13.5 | 1 |
| 1 | MCU | HD | M | 13.5 | 1 |
| 1 | MCU | HD | M | 13.5 | 1 |
| 1 | MCU | HD | M | 13.5 | 1 |
| 1 | MCU | HD | M | 13.5 | 1 |
| 1 | MCU | HD | M | 13.5 | 1 |
| 1 | MCU | HD | M | 13.5 | 1 |
| 1 | MCU | HD | M | 13.5 | 1 |
| 1 | MCU | HD | M | 14.5 | 1 |
| 1 | MCU | HD | M | 14.5 | 1 |
| 1 | MCU | HD | M | 14.5 | 1 |
| 1 | MCU | HD | M | 14.5 | 1 |
| 1 | MCU | HD | M | 14.5 | 1 |
| 1 | MCU | HD | M | 14.5 | 1 |
| 1 | MCU | HD | M | 14.5 | 1 |
| 1 | MCU | HD | M | 14.5 | 1 |
| 1 | MCU | HD | M | 14.5 | 1 |
| 1 | MCU | HD | M | 14.5 | 1 |
| 1 | MCU | HD | M | 16.5 | 1 |
| 1 | MCU | HD | M | 16.5 | 1 |
| 1 | MCU | HD | M | 17.5 | 1 |
| 1 | MCU | HD | M | 17.5 | 1 |
| 1 | MCU | HD | M | 17.5 | 1 |
| 1 | MCU | HD | M | 17.5 | 1 |
| 1 | MCU | HD | M | 17.5 | 1 |
| 1 | MCU | HD | M | 18.5 | 1 |
| 1 | MCU | HD | M | 18.5 | 1 |
| 1 | MCU | HD | M | 18.5 | 1 |
| 1 | MCU | HD | M | 21 | 1 |
| 1 | MCU | HD | M | 32 | 1 |
| 1 | MCU | HD | M | 43 | 1 |
| 1 | MCU | HD | M | 91 | 0 |
| 1 | MCU | HD | M | 91 | 0 |
| 1 | MCU | HD | M | 91 | 0 |
| 1 | MCU | HD | M | 91 | 0 |
| 1 | MCU | LD | M | 8 | 1 |
| 1 | MCU | LD | M | 13.5 | 1 |
| 1 | MCU | LD | M | 13.5 | 1 |
| 1 | MCU | LD | M | 13.5 | 1 |
| 1 | MCU | LD | M | 13.5 | 1 |
| 1 | MCU | LD | M | 13.5 | 1 |
| 1 | MCU | LD | M | 13.5 | 1 |
| 1 | MCU | LD | M | 13.5 | 1 |
| 1 | MCU | LD | M | 14.5 | 1 |
| 1 | MCU | LD | M | 15.5 | 1 |
| 1 | MCU | LD | M | 15.5 | 1 |
| 1 | MCU | LD | M | 15.5 | 1 |
| 1 | MCU | LD | M | 16.5 | 1 |
| 1 | MCU | LD | M | 18.5 | 1 |
| 1 | MCU | LD | M | 18.5 | 1 |
| 1 | MCU | LD | M | 19.5 | 1 |
| 1 | MCU | LD | M | 19.5 | 1 |
| 1 | MCU | LD | M | 19.5 | 1 |
| 1 | MCU | LD | M | 20 | 1 |
| 1 | MCU | LD | M | 20 | 1 |
| 1 | MCU | LD | M | 22 | 1 |
| 1 | MCU | LD | M | 22 | 1 |
| 1 | MCU | LD | M | 22 | 1 |
| 1 | MCU | LD | M | 23 | 1 |
| 1 | MCU | LD | M | 23 | 1 |
| 1 | MCU | LD | M | 23 | 1 |
| 1 | MCU | LD | M | 23 | 1 |
| 1 | MCU | LD | M | 24 | 1 |
| 1 | MCU | LD | M | 24 | 1 |
| 1 | MCU | LD | M | 28 | 1 |
| 1 | MCU | LD | M | 32 | 1 |
| 1 | MCU | LD | M | 45 | 1 |
| 1 | MCU | LD | M | 50 | 1 |
| 1 | MCU | LD | M | 65 | 1 |
| 1 | MCU | LD | M | 91 | 0 |
| 1 | MCU | LD | M | 91 | 0 |
| 1 | MCU | LD | M | 91 | 0 |
| 1 | MCU | LD | M | 91 | 0 |
| 1 | MCU | LD | M | 91 | 0 |
| 1 | MCU | LD | M | 91 | 0 |
| 1 | MCU | LD | M | 91 | 0 |
| 1 | MCU | LD | M | 91 | 0 |
| 1 | MCU | LD | M | 91 | 0 |
| 1 | MCU | LD | M | 91 | 0 |
| 1 | MCU | LD | M | 91 | 0 |
| 1 | MCU | LD | M | 91 | 0 |
| 1 | MCU | LD | M | 91 | 0 |
| 1 | MCU | LD | M | 91 | 0 |
| 1 | MCU | LD | M | 91 | 0 |
| 1 | MCU | LD | M | 91 | 0 |
| 2 | MCU | HD | M | 4 | 1 |
| 2 | MCU | HD | M | 6 | 1 |
| 2 | MCU | HD | M | 14.3 | 1 |
| 2 | MCU | HD | M | 17.3 | 1 |
| 2 | MCU | HD | M | 17.3 | 1 |
| 2 | MCU | HD | M | 17.3 | 1 |
| 2 | MCU | HD | M | 18.3 | 1 |
| 2 | MCU | HD | M | 19.3 | 1 |
| 2 | MCU | HD | M | 20.3 | 1 |
| 2 | MCU | HD | M | 20.3 | 1 |
| 2 | MCU | HD | M | 20.3 | 1 |
| 2 | MCU | HD | M | 22.3 | 1 |
| 2 | MCU | HD | M | 22.3 | 1 |
| 2 | MCU | HD | M | 22.3 | 1 |
| 2 | MCU | HD | M | 23.3 | 1 |
| 2 | MCU | HD | M | 23.3 | 1 |
| 2 | MCU | HD | M | 23.3 | 1 |
| 2 | MCU | HD | M | 23.3 | 1 |
| 2 | MCU | HD | M | 28 | 1 |
| 2 | MCU | HD | M | 28 | 1 |
| 2 | MCU | HD | M | 31 | 1 |
| 2 | MCU | HD | M | 32 | 1 |
| 2 | MCU | HD | M | 33 | 1 |
| 2 | MCU | HD | M | 35.3 | 1 |
| 2 | MCU | HD | M | 45.3 | 1 |
| 2 | MCU | HD | M | 45.3 | 1 |
| 2 | MCU | HD | M | 92 | 0 |
| 2 | MCU | HD | M | 92 | 0 |
| 2 | MCU | HD | M | 92 | 0 |
| 2 | MCU | HD | M | 92 | 0 |
| 2 | MCU | HD | M | 92 | 0 |
| 2 | MCU | HD | M | 92 | 0 |
| 2 | MCU | HD | M | 92 | 0 |
| 2 | MCU | HD | M | 92 | 0 |
| 2 | MCU | HD | M | 92 | 0 |
| 2 | MCU | HD | M | 92 | 0 |
| 2 | MCU | HD | M | 92 | 0 |
| 2 | MCU | HD | M | 92 | 0 |
| 2 | MCU | HD | M | 92 | 0 |
| 2 | MCU | HD | M | 92 | 0 |
| 2 | MCU | HD | M | 92 | 0 |
| 2 | MCU | HD | M | 92 | 0 |
| 2 | MCU | HD | M | 92 | 0 |
| 2 | MCU | HD | M | 92 | 0 |
| 2 | MCU | HD | M | 92 | 0 |
| 2 | MCU | HD | M | 92 | 0 |
| 2 | MCU | HD | M | 92 | 0 |
| 2 | MCU | HD | M | 92 | 0 |
| 2 | MCU | HD | M | 92 | 0 |
| 2 | MCU | LD | M | 15.3 | 0 |
| 2 | MCU | LD | M | 15.3 | 0 |
| 2 | MCU | LD | M | 16.3 | 1 |
| 2 | MCU | LD | M | 17.3 | 1 |
| 2 | MCU | LD | M | 17.3 | 1 |
| 2 | MCU | LD | M | 18.3 | 1 |
| 2 | MCU | LD | M | 18.3 | 1 |
| 2 | MCU | LD | M | 18.3 | 1 |
| 2 | MCU | LD | M | 19.3 | 1 |
| 2 | MCU | LD | M | 19.3 | 1 |
| 2 | MCU | LD | M | 20.3 | 1 |
| 2 | MCU | LD | M | 20.3 | 1 |
| 2 | MCU | LD | M | 20.3 | 1 |
| 2 | MCU | LD | M | 20.3 | 1 |
| 2 | MCU | LD | M | 20.3 | 1 |
| 2 | MCU | LD | M | 20.3 | 1 |
| 2 | MCU | LD | M | 20.3 | 1 |
| 2 | MCU | LD | M | 20.3 | 1 |
| 2 | MCU | LD | M | 21.3 | 1 |
| 2 | MCU | LD | M | 21.3 | 1 |
| 2 | MCU | LD | M | 21.3 | 1 |
| 2 | MCU | LD | M | 23.3 | 1 |
| 2 | MCU | LD | M | 23.3 | 1 |
| 2 | MCU | LD | M | 24.3 | 1 |
| 2 | MCU | LD | M | 24.3 | 1 |
| 2 | MCU | LD | M | 24.3 | 1 |
| 2 | MCU | LD | M | 25.3 | 1 |
| 2 | MCU | LD | M | 25.3 | 1 |
| 2 | MCU | LD | M | 27.3 | 1 |
| 2 | MCU | LD | M | 29.3 | 1 |
| 2 | MCU | LD | M | 30 | 1 |
| 2 | MCU | LD | M | 31 | 1 |
| 2 | MCU | LD | M | 31 | 1 |
| 2 | MCU | LD | M | 31 | 1 |
| 2 | MCU | LD | M | 34 | 1 |
| 2 | MCU | LD | M | 45.3 | 1 |
| 2 | MCU | LD | M | 53 | 1 |
| 2 | MCU | LD | M | 57 | 1 |
| 2 | MCU | LD | M | 69.3 | 1 |
| 2 | MCU | LD | M | 69.3 | 1 |
| 2 | MCU | LD | M | 92 | 0 |
| 2 | MCU | LD | M | 92 | 0 |
| 2 | MCU | LD | M | 92 | 0 |
| 2 | MCU | LD | M | 92 | 0 |
| 2 | MCU | LD | M | 92 | 0 |
| 2 | MCU | LD | M | 92 | 0 |
| 2 | MCU | LD | M | 92 | 0 |
| 2 | MCU | LD | M | 92 | 0 |
| 2 | MCU | LD | M | 92 | 0 |
| 2 | MCU | LD | M | 92 | 0 |
| 2 | MCU | LD | M | 92 | 0 |
| 3 | MCU | HD | M | 7 | 1 |
| 3 | MCU | HD | M | 14 | 1 |
| 3 | MCU | HD | M | 14 | 1 |
| 3 | MCU | HD | M | 14 | 1 |
| 3 | MCU | HD | M | 14 | 1 |
| 3 | MCU | HD | M | 14 | 1 |
| 3 | MCU | HD | M | 14 | 1 |
| 3 | MCU | HD | M | 16 | 1 |
| 3 | MCU | HD | M | 16 | 1 |
| 3 | MCU | HD | M | 16 | 1 |
| 3 | MCU | HD | M | 16 | 1 |
| 3 | MCU | HD | M | 16 | 1 |
| 3 | MCU | HD | M | 16 | 1 |
| 3 | MCU | HD | M | 16 | 1 |
| 3 | MCU | HD | M | 17 | 1 |
| 3 | MCU | HD | M | 18 | 1 |
| 3 | MCU | HD | M | 18 | 1 |
| 3 | MCU | HD | M | 19 | 1 |
| 3 | MCU | HD | M | 19 | 1 |
| 3 | MCU | HD | M | 21 | 1 |
| 3 | MCU | HD | M | 21 | 1 |
| 3 | MCU | HD | M | 21 | 1 |
| 3 | MCU | HD | M | 23 | 1 |
| 3 | MCU | HD | M | 26 | 1 |
| 3 | MCU | HD | M | 26 | 1 |
| 3 | MCU | HD | M | 29 | 1 |
| 3 | MCU | HD | M | 36 | 1 |
| 3 | MCU | HD | M | 37 | 1 |
| 3 | MCU | HD | M | 45 | 1 |
| 3 | MCU | HD | M | 47 | 1 |
| 3 | MCU | HD | M | 47 | 1 |
| 3 | MCU | HD | M | 93 | 0 |
| 3 | MCU | HD | M | 93 | 0 |
| 3 | MCU | HD | M | 93 | 0 |
| 3 | MCU | HD | M | 93 | 0 |
| 3 | MCU | HD | M | 93 | 0 |
| 3 | MCU | HD | M | 93 | 0 |
| 3 | MCU | HD | M | 93 | 0 |
| 3 | MCU | HD | M | 93 | 0 |
| 3 | MCU | HD | M | 93 | 0 |
| 3 | MCU | HD | M | 93 | 0 |
| 3 | MCU | HD | M | 93 | 0 |
| 3 | MCU | HD | M | 93 | 0 |
| 3 | MCU | HD | M | 93 | 0 |
| 3 | MCU | HD | M | 93 | 0 |
| 3 | MCU | HD | M | 93 | 0 |
| 3 | MCU | HD | M | 93 | 0 |
| 3 | MCU | HD | M | 93 | 0 |
| 3 | MCU | HD | M | 93 | 0 |
| 3 | MCU | HD | M | 93 | 0 |
| 3 | MCU | LD | M | 13 | 1 |
| 3 | MCU | LD | M | 14 | 1 |
| 3 | MCU | LD | M | 15 | 1 |
| 3 | MCU | LD | M | 15 | 1 |
| 3 | MCU | LD | M | 15 | 1 |
| 3 | MCU | LD | M | 15 | 1 |
| 3 | MCU | LD | M | 16 | 1 |
| 3 | MCU | LD | M | 17 | 1 |
| 3 | MCU | LD | M | 17 | 1 |
| 3 | MCU | LD | M | 18 | 1 |
| 3 | MCU | LD | M | 18 | 1 |
| 3 | MCU | LD | M | 19 | 1 |
| 3 | MCU | LD | M | 19 | 1 |
| 3 | MCU | LD | M | 20 | 1 |
| 3 | MCU | LD | M | 21 | 1 |
| 3 | MCU | LD | M | 22 | 1 |
| 3 | MCU | LD | M | 22 | 1 |
| 3 | MCU | LD | M | 22 | 1 |
| 3 | MCU | LD | M | 22 | 1 |
| 3 | MCU | LD | M | 22 | 1 |
| 3 | MCU | LD | M | 22 | 1 |
| 3 | MCU | LD | M | 31 | 1 |
| 3 | MCU | LD | M | 31 | 1 |
| 3 | MCU | LD | M | 31 | 1 |
| 3 | MCU | LD | M | 32 | 1 |
| 3 | MCU | LD | M | 48 | 1 |
| 3 | MCU | LD | M | 56 | 1 |
| 3 | MCU | LD | M | 64 | 1 |
| 3 | MCU | LD | M | 64 | 1 |
| 3 | MCU | LD | M | 72 | 1 |
| 3 | MCU | LD | M | 78 | 1 |
| 3 | MCU | LD | M | 78 | 1 |
| 3 | MCU | LD | M | 84 | 1 |
| 3 | MCU | LD | M | 84 | 1 |
| 3 | MCU | LD | M | 87 | 1 |
| 3 | MCU | LD | M | 90 | 1 |
| 3 | MCU | LD | M | 93 | 0 |
| 3 | MCU | LD | M | 93 | 0 |
| 3 | MCU | LD | M | 93 | 0 |
| 3 | MCU | LD | M | 93 | 0 |
| 3 | MCU | LD | M | 93 | 0 |
| 3 | MCU | LD | M | 93 | 0 |
| 3 | MCU | LD | M | 93 | 0 |
| 3 | MCU | LD | M | 93 | 0 |
| 3 | MCU | LD | M | 93 | 0 |
| 3 | MCU | LD | M | 93 | 0 |
| 3 | MCU | LD | M | 93 | 0 |
| 3 | MCU | LD | M | 93 | 0 |
| 3 | MCU | LD | M | 93 | 0 |
| 3 | MCU | LD | M | 93 | 0 |
| 4 | MCU | HD | M | 1 | 1 |
| 4 | MCU | HD | M | 1 | 1 |
| 4 | MCU | HD | M | 10 | 1 |
| 4 | MCU | HD | M | 11 | 1 |
| 4 | MCU | HD | M | 12 | 1 |
| 4 | MCU | HD | M | 13 | 1 |
| 4 | MCU | HD | M | 14 | 1 |
| 4 | MCU | HD | M | 14 | 1 |
| 4 | MCU | HD | M | 14 | 1 |
| 4 | MCU | HD | M | 14 | 1 |
| 4 | MCU | HD | M | 14 | 1 |
| 4 | MCU | HD | M | 15 | 1 |
| 4 | MCU | HD | M | 15 | 1 |
| 4 | MCU | HD | M | 15 | 1 |
| 4 | MCU | HD | M | 15 | 1 |
| 4 | MCU | HD | M | 15 | 1 |
| 4 | MCU | HD | M | 15 | 1 |
| 4 | MCU | HD | M | 16 | 1 |
| 4 | MCU | HD | M | 16 | 1 |
| 4 | MCU | HD | M | 16 | 1 |
| 4 | MCU | HD | M | 16 | 1 |
| 4 | MCU | HD | M | 16 | 1 |
| 4 | MCU | HD | M | 16 | 1 |
| 4 | MCU | HD | M | 17 | 1 |
| 4 | MCU | HD | M | 18 | 1 |
| 4 | MCU | HD | M | 18 | 1 |
| 4 | MCU | HD | M | 18 | 1 |
| 4 | MCU | HD | M | 19 | 1 |
| 4 | MCU | HD | M | 20 | 1 |
| 4 | MCU | HD | M | 21 | 1 |
| 4 | MCU | HD | M | 21 | 1 |
| 4 | MCU | HD | M | 22 | 1 |
| 4 | MCU | HD | M | 22 | 1 |
| 4 | MCU | HD | M | 23 | 1 |
| 4 | MCU | HD | M | 23 | 1 |
| 4 | MCU | HD | M | 25 | 1 |
| 4 | MCU | HD | M | 25 | 1 |
| 4 | MCU | HD | M | 26 | 1 |
| 4 | MCU | HD | M | 28 | 1 |
| 4 | MCU | HD | M | 44 | 1 |
| 4 | MCU | HD | M | 87 | 1 |
| 4 | MCU | HD | M | 92 | 0 |
| 4 | MCU | HD | M | 92 | 0 |
| 4 | MCU | HD | M | 92 | 0 |
| 4 | MCU | HD | M | 92 | 0 |
| 4 | MCU | HD | M | 92 | 0 |
| 4 | MCU | HD | M | 92 | 0 |
| 4 | MCU | HD | M | 92 | 0 |
| 4 | MCU | LD | M | 14 | 1 |
| 4 | MCU | LD | M | 14 | 1 |
| 4 | MCU | LD | M | 15 | 1 |
| 4 | MCU | LD | M | 15 | 1 |
| 4 | MCU | LD | M | 16 | 1 |
| 4 | MCU | LD | M | 16 | 1 |
| 4 | MCU | LD | M | 16 | 1 |
| 4 | MCU | LD | M | 16 | 1 |
| 4 | MCU | LD | M | 17 | 1 |
| 4 | MCU | LD | M | 17 | 1 |
| 4 | MCU | LD | M | 17 | 1 |
| 4 | MCU | LD | M | 18 | 1 |
| 4 | MCU | LD | M | 20 | 1 |
| 4 | MCU | LD | M | 20 | 1 |
| 4 | MCU | LD | M | 20 | 1 |
| 4 | MCU | LD | M | 20 | 1 |
| 4 | MCU | LD | M | 21 | 1 |
| 4 | MCU | LD | M | 21 | 1 |
| 4 | MCU | LD | M | 22 | 1 |
| 4 | MCU | LD | M | 23 | 1 |
| 4 | MCU | LD | M | 23 | 1 |
| 4 | MCU | LD | M | 24 | 1 |
| 4 | MCU | LD | M | 24 | 1 |
| 4 | MCU | LD | M | 25 | 1 |
| 4 | MCU | LD | M | 26 | 1 |
| 4 | MCU | LD | M | 27 | 1 |
| 4 | MCU | LD | M | 28 | 1 |
| 4 | MCU | LD | M | 29 | 1 |
| 4 | MCU | LD | M | 29 | 1 |
| 4 | MCU | LD | M | 32 | 1 |
| 4 | MCU | LD | M | 34 | 1 |
| 4 | MCU | LD | M | 34 | 1 |
| 4 | MCU | LD | M | 42 | 1 |
| 4 | MCU | LD | M | 47 | 1 |
| 4 | MCU | LD | M | 72 | 1 |
| 4 | MCU | LD | M | 75 | 1 |
| 4 | MCU | LD | M | 75 | 1 |
| 4 | MCU | LD | M | 75 | 1 |
| 4 | MCU | LD | M | 92 | 0 |
| 4 | MCU | LD | M | 92 | 0 |
| 4 | MCU | LD | M | 92 | 0 |
| 4 | MCU | LD | M | 92 | 0 |
| 4 | MCU | LD | M | 92 | 0 |
| 4 | MCU | LD | M | 92 | 0 |
| 4 | MCU | LD | M | 92 | 0 |
| 4 | MCU | LD | M | 92 | 0 |
| 4 | MCU | LD | M | 92 | 0 |
| 4 | MCU | LD | M | 92 | 0 |
| 1 | MB | HD | F | 2 | 1 |
| 1 | MB | HD | F | 12 | 1 |
| 1 | MB | HD | F | 12 | 1 |
| 1 | MB | HD | F | 13.5 | 1 |
| 1 | MB | HD | F | 13.5 | 1 |
| 1 | MB | HD | F | 13.5 | 1 |
| 1 | MB | HD | F | 13.5 | 1 |
| 1 | MB | HD | F | 13.5 | 1 |
| 1 | MB | HD | F | 13.5 | 1 |
| 1 | MB | HD | F | 13.5 | 1 |
| 1 | MB | HD | F | 13.5 | 1 |
| 1 | MB | HD | F | 13.5 | 1 |
| 1 | MB | HD | F | 13.5 | 1 |
| 1 | MB | HD | F | 14.5 | 1 |
| 1 | MB | HD | F | 14.5 | 1 |
| 1 | MB | HD | F | 14.5 | 1 |
| 1 | MB | HD | F | 14.5 | 1 |
| 1 | MB | HD | F | 14.5 | 1 |
| 1 | MB | HD | F | 14.5 | 1 |
| 1 | MB | HD | F | 15.5 | 1 |
| 1 | MB | HD | F | 15.5 | 1 |
| 1 | MB | HD | F | 15.5 | 1 |
| 1 | MB | HD | F | 15.5 | 1 |
| 1 | MB | HD | F | 15.5 | 1 |
| 1 | MB | HD | F | 15.5 | 1 |
| 1 | MB | HD | F | 16.5 | 1 |
| 1 | MB | HD | F | 16.5 | 1 |
| 1 | MB | HD | F | 16.5 | 1 |
| 1 | MB | HD | F | 16.5 | 1 |
| 1 | MB | HD | F | 17.5 | 1 |
| 1 | MB | HD | F | 17.5 | 1 |
| 1 | MB | HD | F | 17.5 | 1 |
| 1 | MB | HD | F | 17.5 | 1 |
| 1 | MB | HD | F | 17.5 | 1 |
| 1 | MB | HD | F | 17.5 | 1 |
| 1 | MB | HD | F | 17.5 | 1 |
| 1 | MB | HD | F | 18.5 | 1 |
| 1 | MB | HD | F | 18.5 | 1 |
| 1 | MB | HD | F | 18.5 | 1 |
| 1 | MB | HD | F | 19.5 | 1 |
| 1 | MB | HD | F | 19.5 | 1 |
| 1 | MB | HD | F | 21 | 1 |
| 1 | MB | HD | F | 22 | 1 |
| 1 | MB | HD | F | 26 | 1 |
| 1 | MB | HD | F | 34 | 1 |
| 1 | MB | HD | F | 34 | 1 |
| 1 | MB | HD | F | 50 | 1 |
| 1 | MB | HD | F | 91 | 0 |
| 1 | MB | HD | F | 91 | 0 |
| 1 | MB | HD | F | 91 | 0 |
| 1 | MB | LD | F | 13.5 | 1 |
| 1 | MB | LD | F | 13.5 | 1 |
| 1 | MB | LD | F | 13.5 | 1 |
| 1 | MB | LD | F | 13.5 | 1 |
| 1 | MB | LD | F | 13.5 | 1 |
| 1 | MB | LD | F | 13.5 | 1 |
| 1 | MB | LD | F | 14.5 | 1 |
| 1 | MB | LD | F | 15.5 | 1 |
| 1 | MB | LD | F | 15.5 | 1 |
| 1 | MB | LD | F | 16.5 | 1 |
| 1 | MB | LD | F | 17.5 | 1 |
| 1 | MB | LD | F | 17.5 | 1 |
| 1 | MB | LD | F | 17.5 | 1 |
| 1 | MB | LD | F | 17.5 | 1 |
| 1 | MB | LD | F | 18.5 | 1 |
| 1 | MB | LD | F | 18.5 | 1 |
| 1 | MB | LD | F | 19.5 | 1 |
| 1 | MB | LD | F | 19.5 | 1 |
| 1 | MB | LD | F | 21 | 1 |
| 1 | MB | LD | F | 21 | 1 |
| 1 | MB | LD | F | 21 | 1 |
| 1 | MB | LD | F | 22 | 1 |
| 1 | MB | LD | F | 22 | 1 |
| 1 | MB | LD | F | 22 | 1 |
| 1 | MB | LD | F | 22 | 1 |
| 1 | MB | LD | F | 23 | 1 |
| 1 | MB | LD | F | 23 | 1 |
| 1 | MB | LD | F | 24 | 1 |
| 1 | MB | LD | F | 24 | 1 |
| 1 | MB | LD | F | 26 | 1 |
| 1 | MB | LD | F | 28 | 1 |
| 1 | MB | LD | F | 28 | 1 |
| 1 | MB | LD | F | 32 | 1 |
| 1 | MB | LD | F | 32 | 1 |
| 1 | MB | LD | F | 36 | 1 |
| 1 | MB | LD | F | 36 | 1 |
| 1 | MB | LD | F | 36 | 1 |
| 1 | MB | LD | F | 37 | 1 |
| 1 | MB | LD | F | 37 | 1 |
| 1 | MB | LD | F | 39 | 1 |
| 1 | MB | LD | F | 41 | 1 |
| 1 | MB | LD | F | 45 | 1 |
| 1 | MB | LD | F | 51 | 1 |
| 1 | MB | LD | F | 51 | 1 |
| 1 | MB | LD | F | 51 | 1 |
| 1 | MB | LD | F | 62 | 1 |
| 1 | MB | LD | F | 91 | 1 |
| 1 | MB | LD | F | 91 | 0 |
| 1 | MB | LD | F | 91 | 0 |
| 1 | MB | LD | F | 91 | 0 |
| 2 | MB | LD | F | 10.3 | 1 |
| 2 | MB | LD | F | 10.3 | 1 |
| 2 | MB | LD | F | 13.3 | 1 |
| 2 | MB | LD | F | 14.3 | 1 |
| 2 | MB | LD | F | 15.3 | 1 |
| 2 | MB | LD | F | 17.3 | 1 |
| 2 | MB | LD | F | 18.3 | 1 |
| 2 | MB | LD | F | 18.3 | 1 |
| 2 | MB | LD | F | 18.3 | 1 |
| 2 | MB | LD | F | 19.3 | 1 |
| 2 | MB | LD | F | 19.3 | 1 |
| 2 | MB | LD | F | 20.3 | 1 |
| 2 | MB | LD | F | 20.3 | 1 |
| 2 | MB | LD | F | 20.3 | 1 |
| 2 | MB | LD | F | 20.3 | 1 |
| 2 | MB | LD | F | 21.3 | 1 |
| 2 | MB | LD | F | 21.3 | 1 |
| 2 | MB | LD | F | 21.3 | 1 |
| 2 | MB | LD | F | 22.3 | 1 |
| 2 | MB | LD | F | 22.3 | 1 |
| 2 | MB | LD | F | 23.3 | 1 |
| 2 | MB | LD | F | 23.3 | 1 |
| 2 | MB | LD | F | 23.3 | 1 |
| 2 | MB | LD | F | 24.3 | 1 |
| 2 | MB | LD | F | 24.3 | 1 |
| 2 | MB | LD | F | 26 | 1 |
| 2 | MB | LD | F | 26 | 1 |
| 2 | MB | LD | F | 27 | 1 |
| 2 | MB | LD | F | 28 | 1 |
| 2 | MB | LD | F | 31 | 1 |
| 2 | MB | LD | F | 31 | 1 |
| 2 | MB | LD | F | 31 | 1 |
| 2 | MB | LD | F | 31 | 1 |
| 2 | MB | LD | F | 32 | 1 |
| 2 | MB | LD | F | 35.3 | 1 |
| 2 | MB | LD | F | 35.3 | 1 |
| 2 | MB | LD | F | 38.3 | 1 |
| 2 | MB | LD | F | 39.3 | 1 |
| 2 | MB | LD | F | 40.3 | 1 |
| 2 | MB | LD | F | 41.3 | 1 |
| 2 | MB | LD | F | 42.3 | 1 |
| 2 | MB | LD | F | 45.3 | 1 |
| 2 | MB | LD | F | 53 | 1 |
| 2 | MB | LD | F | 53 | 1 |
| 2 | MB | LD | F | 92 | 1 |
| 2 | MB | LD | F | 92 | 0 |
| 2 | MB | LD | F | 92 | 0 |
| 2 | MB | LD | F | 92 | 0 |
| 2 | MB | LD | F | 92 | 0 |
| 2 | MB | HD | F | 12.3 | 1 |
| 2 | MB | HD | F | 12.3 | 1 |
| 2 | MB | HD | F | 12.3 | 1 |
| 2 | MB | HD | F | 13.3 | 1 |
| 2 | MB | HD | F | 13.3 | 1 |
| 2 | MB | HD | F | 13.3 | 1 |
| 2 | MB | HD | F | 13.3 | 1 |
| 2 | MB | HD | F | 14.3 | 1 |
| 2 | MB | HD | F | 14.3 | 1 |
| 2 | MB | HD | F | 14.3 | 1 |
| 2 | MB | HD | F | 14.3 | 1 |
| 2 | MB | HD | F | 15.3 | 1 |
| 2 | MB | HD | F | 15.3 | 1 |
| 2 | MB | HD | F | 15.3 | 1 |
| 2 | MB | HD | F | 15.3 | 1 |
| 2 | MB | HD | F | 16.3 | 1 |
| 2 | MB | HD | F | 17.3 | 1 |
| 2 | MB | HD | F | 17.3 | 1 |
| 2 | MB | HD | F | 17.3 | 1 |
| 2 | MB | HD | F | 18.3 | 1 |
| 2 | MB | HD | F | 18.3 | 1 |
| 2 | MB | HD | F | 18.3 | 1 |
| 2 | MB | HD | F | 19.3 | 1 |
| 2 | MB | HD | F | 19.3 | 1 |
| 2 | MB | HD | F | 19.3 | 1 |
| 2 | MB | HD | F | 19.3 | 1 |
| 2 | MB | HD | F | 19.3 | 1 |
| 2 | MB | HD | F | 20.3 | 1 |
| 2 | MB | HD | F | 20.3 | 1 |
| 2 | MB | HD | F | 20.3 | 1 |
| 2 | MB | HD | F | 21.3 | 1 |
| 2 | MB | HD | F | 21.3 | 1 |
| 2 | MB | HD | F | 23.3 | 1 |
| 2 | MB | HD | F | 23.3 | 1 |
| 2 | MB | HD | F | 24.3 | 1 |
| 2 | MB | HD | F | 26.3 | 1 |
| 2 | MB | HD | F | 26.3 | 1 |
| 2 | MB | HD | F | 29.3 | 1 |
| 2 | MB | HD | F | 30 | 1 |
| 2 | MB | HD | F | 60.3 | 1 |
| 2 | MB | HD | F | 92 | 0 |
| 2 | MB | HD | F | 92 | 0 |
| 2 | MB | HD | F | 92 | 0 |
| 2 | MB | HD | F | 92 | 0 |
| 2 | MB | HD | F | 92 | 0 |
| 2 | MB | HD | F | 92 | 0 |
| 2 | MB | HD | F | 92 | 0 |
| 2 | MB | HD | F | 92 | 0 |
| 2 | MB | HD | F | 92 | 0 |
| 2 | MB | HD | F | 92 | 0 |
| 3 | MB | HD | F | 14 | 1 |
| 3 | MB | HD | F | 14 | 1 |
| 3 | MB | HD | F | 14 | 1 |
| 3 | MB | HD | F | 14 | 1 |
| 3 | MB | HD | F | 15 | 1 |
| 3 | MB | HD | F | 15 | 1 |
| 3 | MB | HD | F | 15 | 1 |
| 3 | MB | HD | F | 16 | 1 |
| 3 | MB | HD | F | 16 | 1 |
| 3 | MB | HD | F | 16 | 1 |
| 3 | MB | HD | F | 16 | 1 |
| 3 | MB | HD | F | 17 | 1 |
| 3 | MB | HD | F | 17 | 1 |
| 3 | MB | HD | F | 17 | 1 |
| 3 | MB | HD | F | 17 | 1 |
| 3 | MB | HD | F | 17 | 1 |
| 3 | MB | HD | F | 18 | 1 |
| 3 | MB | HD | F | 18 | 1 |
| 3 | MB | HD | F | 19 | 1 |
| 3 | MB | HD | F | 19 | 1 |
| 3 | MB | HD | F | 20 | 1 |
| 3 | MB | HD | F | 20 | 1 |
| 3 | MB | HD | F | 20 | 1 |
| 3 | MB | HD | F | 21 | 1 |
| 3 | MB | HD | F | 21 | 1 |
| 3 | MB | HD | F | 21 | 1 |
| 3 | MB | HD | F | 22 | 1 |
| 3 | MB | HD | F | 23 | 1 |
| 3 | MB | HD | F | 23 | 1 |
| 3 | MB | HD | F | 23 | 1 |
| 3 | MB | HD | F | 24 | 1 |
| 3 | MB | HD | F | 27 | 1 |
| 3 | MB | HD | F | 33 | 1 |
| 3 | MB | HD | F | 33 | 1 |
| 3 | MB | HD | F | 33 | 1 |
| 3 | MB | HD | F | 37 | 1 |
| 3 | MB | HD | F | 37 | 1 |
| 3 | MB | HD | F | 39 | 1 |
| 3 | MB | HD | F | 45 | 1 |
| 3 | MB | HD | F | 64 | 1 |
| 3 | MB | HD | F | 64 | 1 |
| 3 | MB | HD | F | 84 | 1 |
| 3 | MB | HD | F | 93 | 0 |
| 3 | MB | HD | F | 93 | 0 |
| 3 | MB | HD | F | 93 | 0 |
| 3 | MB | HD | F | 93 | 0 |
| 3 | MB | HD | F | 93 | 0 |
| 3 | MB | HD | F | 93 | 0 |
| 3 | MB | HD | F | 93 | 0 |
| 3 | MB | HD | F | 93 | 0 |
| 3 | MB | LD | F | 17 | 1 |
| 3 | MB | LD | F | 17 | 1 |
| 3 | MB | LD | F | 18 | 1 |
| 3 | MB | LD | F | 19 | 1 |
| 3 | MB | LD | F | 20 | 1 |
| 3 | MB | LD | F | 20 | 1 |
| 3 | MB | LD | F | 20 | 1 |
| 3 | MB | LD | F | 22 | 1 |
| 3 | MB | LD | F | 22 | 1 |
| 3 | MB | LD | F | 22 | 1 |
| 3 | MB | LD | F | 22 | 1 |
| 3 | MB | LD | F | 24 | 1 |
| 3 | MB | LD | F | 24 | 1 |
| 3 | MB | LD | F | 24 | 1 |
| 3 | MB | LD | F | 24 | 1 |
| 3 | MB | LD | F | 25 | 1 |
| 3 | MB | LD | F | 26 | 1 |
| 3 | MB | LD | F | 27 | 1 |
| 3 | MB | LD | F | 27 | 1 |
| 3 | MB | LD | F | 28 | 1 |
| 3 | MB | LD | F | 28 | 1 |
| 3 | MB | LD | F | 28 | 1 |
| 3 | MB | LD | F | 28 | 1 |
| 3 | MB | LD | F | 28 | 1 |
| 3 | MB | LD | F | 31 | 1 |
| 3 | MB | LD | F | 31 | 1 |
| 3 | MB | LD | F | 31 | 1 |
| 3 | MB | LD | F | 36 | 1 |
| 3 | MB | LD | F | 36 | 1 |
| 3 | MB | LD | F | 37 | 1 |
| 3 | MB | LD | F | 37 | 1 |
| 3 | MB | LD | F | 37 | 1 |
| 3 | MB | LD | F | 37 | 1 |
| 3 | MB | LD | F | 39 | 1 |
| 3 | MB | LD | F | 41 | 1 |
| 3 | MB | LD | F | 47 | 1 |
| 3 | MB | LD | F | 47 | 1 |
| 3 | MB | LD | F | 47 | 1 |
| 3 | MB | LD | F | 47 | 1 |
| 3 | MB | LD | F | 47 | 1 |
| 3 | MB | LD | F | 48 | 1 |
| 3 | MB | LD | F | 48 | 1 |
| 3 | MB | LD | F | 48 | 1 |
| 3 | MB | LD | F | 48 | 1 |
| 3 | MB | LD | F | 48 | 1 |
| 3 | MB | LD | F | 66 | 1 |
| 3 | MB | LD | F | 70 | 1 |
| 3 | MB | LD | F | 78 | 1 |
| 3 | MB | LD | F | 93 | 0 |
| 3 | MB | LD | F | 93 | 0 |
| 4 | MB | HD | F | 1 | 1 |
| 4 | MB | HD | F | 2 | 1 |
| 4 | MB | HD | F | 14 | 1 |
| 4 | MB | HD | F | 14 | 1 |
| 4 | MB | HD | F | 14 | 1 |
| 4 | MB | HD | F | 14 | 1 |
| 4 | MB | HD | F | 14 | 1 |
| 4 | MB | HD | F | 15 | 1 |
| 4 | MB | HD | F | 15 | 1 |
| 4 | MB | HD | F | 15 | 1 |
| 4 | MB | HD | F | 15 | 1 |
| 4 | MB | HD | F | 15 | 1 |
| 4 | MB | HD | F | 15 | 1 |
| 4 | MB | HD | F | 16 | 1 |
| 4 | MB | HD | F | 16 | 1 |
| 4 | MB | HD | F | 17 | 1 |
| 4 | MB | HD | F | 18 | 1 |
| 4 | MB | HD | F | 18 | 1 |
| 4 | MB | HD | F | 18 | 1 |
| 4 | MB | HD | F | 18 | 1 |
| 4 | MB | HD | F | 18 | 1 |
| 4 | MB | HD | F | 19 | 1 |
| 4 | MB | HD | F | 19 | 1 |
| 4 | MB | HD | F | 19 | 1 |
| 4 | MB | HD | F | 19 | 1 |
| 4 | MB | HD | F | 20 | 1 |
| 4 | MB | HD | F | 20 | 1 |
| 4 | MB | HD | F | 20 | 1 |
| 4 | MB | HD | F | 20 | 1 |
| 4 | MB | HD | F | 20 | 1 |
| 4 | MB | HD | F | 20 | 1 |
| 4 | MB | HD | F | 20 | 1 |
| 4 | MB | HD | F | 20 | 1 |
| 4 | MB | HD | F | 21 | 1 |
| 4 | MB | HD | F | 21 | 1 |
| 4 | MB | HD | F | 23 | 1 |
| 4 | MB | HD | F | 23 | 1 |
| 4 | MB | HD | F | 24 | 1 |
| 4 | MB | HD | F | 24 | 1 |
| 4 | MB | HD | F | 25 | 1 |
| 4 | MB | HD | F | 25 | 1 |
| 4 | MB | HD | F | 31 | 1 |
| 4 | MB | HD | F | 47 | 1 |
| 4 | MB | HD | F | 87 | 1 |
| 4 | MB | HD | F | 92 | 1 |
| 4 | MB | HD | F | 92 | 0 |
| 4 | MB | HD | F | 92 | 0 |
| 4 | MB | HD | F | 92 | 0 |
| 4 | MB | LD | F | 3 | 1 |
| 4 | MB | LD | F | 16 | 1 |
| 4 | MB | LD | F | 16 | 1 |
| 4 | MB | LD | F | 17 | 1 |
| 4 | MB | LD | F | 19 | 1 |
| 4 | MB | LD | F | 19 | 1 |
| 4 | MB | LD | F | 19 | 1 |
| 4 | MB | LD | F | 19 | 1 |
| 4 | MB | LD | F | 19 | 1 |
| 4 | MB | LD | F | 20 | 1 |
| 4 | MB | LD | F | 20 | 1 |
| 4 | MB | LD | F | 20 | 1 |
| 4 | MB | LD | F | 20 | 1 |
| 4 | MB | LD | F | 20 | 1 |
| 4 | MB | LD | F | 22 | 1 |
| 4 | MB | LD | F | 22 | 1 |
| 4 | MB | LD | F | 22 | 1 |
| 4 | MB | LD | F | 22 | 1 |
| 4 | MB | LD | F | 24 | 1 |
| 4 | MB | LD | F | 25 | 1 |
| 4 | MB | LD | F | 25 | 1 |
| 4 | MB | LD | F | 25 | 1 |
| 4 | MB | LD | F | 26 | 1 |
| 4 | MB | LD | F | 26 | 1 |
| 4 | MB | LD | F | 26 | 1 |
| 4 | MB | LD | F | 28 | 1 |
| 4 | MB | LD | F | 29 | 1 |
| 4 | MB | LD | F | 29 | 1 |
| 4 | MB | LD | F | 30 | 1 |
| 4 | MB | LD | F | 32 | 1 |
| 4 | MB | LD | F | 33 | 1 |
| 4 | MB | LD | F | 34 | 1 |
| 4 | MB | LD | F | 35 | 1 |
| 4 | MB | LD | F | 35 | 1 |
| 4 | MB | LD | F | 35 | 1 |
| 4 | MB | LD | F | 36 | 1 |
| 4 | MB | LD | F | 38 | 1 |
| 4 | MB | LD | F | 38 | 1 |
| 4 | MB | LD | F | 38 | 1 |
| 4 | MB | LD | F | 53 | 1 |
| 4 | MB | LD | F | 53 | 1 |
| 4 | MB | LD | F | 56 | 1 |
| 4 | MB | LD | F | 78 | 1 |
| 4 | MB | LD | F | 92 | 0 |
| 4 | MB | LD | F | 92 | 0 |
| 4 | MB | LD | F | 92 | 0 |
| 4 | MB | LD | F | 92 | 0 |
| 4 | MB | LD | F | 92 | 0 |
| 1 | MB | HD | M | 2 | 1 |
| 1 | MB | HD | M | 7 | 1 |
| 1 | MB | HD | M | 11.5 | 1 |
| 1 | MB | HD | M | 11.5 | 1 |
| 1 | MB | HD | M | 11.5 | 1 |
| 1 | MB | HD | M | 11.5 | 1 |
| 1 | MB | HD | M | 11.5 | 1 |
| 1 | MB | HD | M | 11.5 | 1 |
| 1 | MB | HD | M | 11.5 | 1 |
| 1 | MB | HD | M | 11.5 | 1 |
| 1 | MB | HD | M | 11.5 | 1 |
| 1 | MB | HD | M | 11.5 | 1 |
| 1 | MB | HD | M | 11.5 | 1 |
| 1 | MB | HD | M | 11.5 | 1 |
| 1 | MB | HD | M | 13.5 | 1 |
| 1 | MB | HD | M | 13.5 | 1 |
| 1 | MB | HD | M | 13.5 | 1 |
| 1 | MB | HD | M | 13.5 | 1 |
| 1 | MB | HD | M | 13.5 | 1 |
| 1 | MB | HD | M | 13.5 | 1 |
| 1 | MB | HD | M | 13.5 | 1 |
| 1 | MB | HD | M | 13.5 | 1 |
| 1 | MB | HD | M | 14.5 | 1 |
| 1 | MB | HD | M | 14.5 | 1 |
| 1 | MB | HD | M | 14.5 | 1 |
| 1 | MB | HD | M | 15.5 | 1 |
| 1 | MB | HD | M | 15.5 | 1 |
| 1 | MB | HD | M | 15.5 | 1 |
| 1 | MB | HD | M | 15.5 | 1 |
| 1 | MB | HD | M | 15.5 | 1 |
| 1 | MB | HD | M | 15.5 | 1 |
| 1 | MB | HD | M | 16.5 | 1 |
| 1 | MB | HD | M | 16.5 | 1 |
| 1 | MB | HD | M | 16.5 | 1 |
| 1 | MB | HD | M | 16.5 | 1 |
| 1 | MB | HD | M | 16.5 | 1 |
| 1 | MB | HD | M | 17.5 | 1 |
| 1 | MB | HD | M | 17.5 | 1 |
| 1 | MB | HD | M | 18.5 | 1 |
| 1 | MB | HD | M | 18.5 | 1 |
| 1 | MB | HD | M | 19.5 | 1 |
| 1 | MB | HD | M | 19.5 | 1 |
| 1 | MB | HD | M | 19.5 | 1 |
| 1 | MB | HD | M | 19.5 | 1 |
| 1 | MB | HD | M | 19.5 | 1 |
| 1 | MB | HD | M | 91 | 0 |
| 1 | MB | HD | M | 91 | 0 |
| 1 | MB | HD | M | 91 | 0 |
| 1 | MB | HD | M | 91 | 0 |
| 1 | MB | HD | M | 91 | 0 |
| 1 | MB | LD | M | 13.5 | 1 |
| 1 | MB | LD | M | 13.5 | 1 |
| 1 | MB | LD | M | 13.5 | 1 |
| 1 | MB | LD | M | 13.5 | 1 |
| 1 | MB | LD | M | 13.5 | 1 |
| 1 | MB | LD | M | 13.5 | 1 |
| 1 | MB | LD | M | 13.5 | 1 |
| 1 | MB | LD | M | 13.5 | 1 |
| 1 | MB | LD | M | 13.5 | 1 |
| 1 | MB | LD | M | 14.5 | 1 |
| 1 | MB | LD | M | 14.5 | 1 |
| 1 | MB | LD | M | 15.5 | 1 |
| 1 | MB | LD | M | 15.5 | 1 |
| 1 | MB | LD | M | 15.5 | 1 |
| 1 | MB | LD | M | 15.5 | 1 |
| 1 | MB | LD | M | 16.5 | 1 |
| 1 | MB | LD | M | 16.5 | 1 |
| 1 | MB | LD | M | 16.5 | 1 |
| 1 | MB | LD | M | 17.5 | 1 |
| 1 | MB | LD | M | 17.5 | 1 |
| 1 | MB | LD | M | 17.5 | 1 |
| 1 | MB | LD | M | 17.5 | 1 |
| 1 | MB | LD | M | 17.5 | 1 |
| 1 | MB | LD | M | 18.5 | 1 |
| 1 | MB | LD | M | 18.5 | 1 |
| 1 | MB | LD | M | 18.5 | 1 |
| 1 | MB | LD | M | 18.5 | 1 |
| 1 | MB | LD | M | 19.5 | 1 |
| 1 | MB | LD | M | 19.5 | 1 |
| 1 | MB | LD | M | 22 | 1 |
| 1 | MB | LD | M | 23 | 1 |
| 1 | MB | LD | M | 23 | 1 |
| 1 | MB | LD | M | 24 | 1 |
| 1 | MB | LD | M | 25 | 1 |
| 1 | MB | LD | M | 26 | 1 |
| 1 | MB | LD | M | 26 | 1 |
| 1 | MB | LD | M | 32 | 1 |
| 1 | MB | LD | M | 43 | 1 |
| 1 | MB | LD | M | 51 | 1 |
| 1 | MB | LD | M | 51 | 1 |
| 1 | MB | LD | M | 68 | 1 |
| 1 | MB | LD | M | 91 | 0 |
| 1 | MB | LD | M | 91 | 0 |
| 1 | MB | LD | M | 91 | 0 |
| 1 | MB | LD | M | 91 | 0 |
| 1 | MB | LD | M | 91 | 0 |
| 1 | MB | LD | M | 91 | 0 |
| 1 | MB | LD | M | 91 | 0 |
| 1 | MB | LD | M | 91 | 0 |
| 1 | MB | LD | M | 91 | 0 |
| 2 | MB | HD | M | 12.3 | 1 |
| 2 | MB | HD | M | 13.3 | 1 |
| 2 | MB | HD | M | 16.3 | 1 |
| 2 | MB | HD | M | 16.3 | 1 |
| 2 | MB | HD | M | 16.3 | 1 |
| 2 | MB | HD | M | 16.3 | 1 |
| 2 | MB | HD | M | 16.3 | 1 |
| 2 | MB | HD | M | 16.3 | 1 |
| 2 | MB | HD | M | 16.3 | 1 |
| 2 | MB | HD | M | 16.3 | 1 |
| 2 | MB | HD | M | 16.3 | 1 |
| 2 | MB | HD | M | 16.3 | 1 |
| 2 | MB | HD | M | 17.3 | 1 |
| 2 | MB | HD | M | 17.3 | 1 |
| 2 | MB | HD | M | 17.3 | 1 |
| 2 | MB | HD | M | 17.3 | 1 |
| 2 | MB | HD | M | 17.3 | 1 |
| 2 | MB | HD | M | 17.3 | 1 |
| 2 | MB | HD | M | 17.3 | 1 |
| 2 | MB | HD | M | 17.3 | 1 |
| 2 | MB | HD | M | 18.3 | 1 |
| 2 | MB | HD | M | 18.3 | 1 |
| 2 | MB | HD | M | 18.3 | 1 |
| 2 | MB | HD | M | 18.3 | 1 |
| 2 | MB | HD | M | 19.3 | 1 |
| 2 | MB | HD | M | 19.3 | 1 |
| 2 | MB | HD | M | 19.3 | 1 |
| 2 | MB | HD | M | 20.3 | 1 |
| 2 | MB | HD | M | 20.3 | 1 |
| 2 | MB | HD | M | 21.3 | 1 |
| 2 | MB | HD | M | 21.3 | 1 |
| 2 | MB | HD | M | 21.3 | 1 |
| 2 | MB | HD | M | 21.3 | 1 |
| 2 | MB | HD | M | 21.3 | 1 |
| 2 | MB | HD | M | 22.3 | 1 |
| 2 | MB | HD | M | 24.3 | 1 |
| 2 | MB | HD | M | 24.3 | 1 |
| 2 | MB | HD | M | 24.3 | 1 |
| 2 | MB | HD | M | 36.3 | 1 |
| 2 | MB | HD | M | 38.3 | 1 |
| 2 | MB | HD | M | 45.3 | 1 |
| 2 | MB | HD | M | 45.3 | 1 |
| 2 | MB | HD | M | 45.3 | 1 |
| 2 | MB | HD | M | 57 | 1 |
| 2 | MB | HD | M | 92 | 0 |
| 2 | MB | HD | M | 92 | 0 |
| 2 | MB | HD | M | 92 | 0 |
| 2 | MB | HD | M | 92 | 0 |
| 2 | MB | HD | M | 92 | 0 |
| 2 | MB | HD | M | 92 | 0 |
| 2 | MB | LD | M | 16.3 | 1 |
| 2 | MB | LD | M | 17.3 | 1 |
| 2 | MB | LD | M | 17.3 | 1 |
| 2 | MB | LD | M | 18.3 | 1 |
| 2 | MB | LD | M | 18.3 | 1 |
| 2 | MB | LD | M | 18.3 | 1 |
| 2 | MB | LD | M | 19.3 | 1 |
| 2 | MB | LD | M | 20.3 | 1 |
| 2 | MB | LD | M | 23.3 | 1 |
| 2 | MB | LD | M | 23.3 | 1 |
| 2 | MB | LD | M | 25.3 | 1 |
| 2 | MB | LD | M | 26.3 | 1 |
| 2 | MB | LD | M | 27.3 | 1 |
| 2 | MB | LD | M | 27.3 | 1 |
| 2 | MB | LD | M | 28.3 | 1 |
| 2 | MB | LD | M | 28.3 | 1 |
| 2 | MB | LD | M | 29.3 | 1 |
| 2 | MB | LD | M | 31 | 1 |
| 2 | MB | LD | M | 32 | 1 |
| 2 | MB | LD | M | 34 | 1 |
| 2 | MB | LD | M | 35.3 | 1 |
| 2 | MB | LD | M | 42.3 | 1 |
| 2 | MB | LD | M | 45.3 | 1 |
| 2 | MB | LD | M | 45.3 | 1 |
| 2 | MB | LD | M | 45.3 | 1 |
| 2 | MB | LD | M | 53 | 1 |
| 2 | MB | LD | M | 57 | 1 |
| 2 | MB | LD | M | 64.3 | 1 |
| 2 | MB | LD | M | 64.3 | 1 |
| 2 | MB | LD | M | 69.3 | 1 |
| 2 | MB | LD | M | 87.3 | 1 |
| 2 | MB | LD | M | 87.3 | 1 |
| 2 | MB | LD | M | 87.3 | 1 |
| 2 | MB | LD | M | 87.3 | 1 |
| 2 | MB | LD | M | 87.3 | 1 |
| 2 | MB | LD | M | 92 | 0 |
| 3 | MB | HD | M | 3 | 1 |
| 3 | MB | HD | M | 11 | 1 |
| 3 | MB | HD | M | 11 | 1 |
| 3 | MB | HD | M | 13 | 1 |
| 3 | MB | HD | M | 13 | 1 |
| 3 | MB | HD | M | 13 | 1 |
| 3 | MB | HD | M | 13 | 1 |
| 3 | MB | HD | M | 14 | 1 |
| 3 | MB | HD | M | 14 | 1 |
| 3 | MB | HD | M | 14 | 1 |
| 3 | MB | HD | M | 14 | 1 |
| 3 | MB | HD | M | 14 | 1 |
| 3 | MB | HD | M | 14 | 1 |
| 3 | MB | HD | M | 14 | 1 |
| 3 | MB | HD | M | 14 | 1 |
| 3 | MB | HD | M | 14 | 1 |
| 3 | MB | HD | M | 15 | 1 |
| 3 | MB | HD | M | 15 | 1 |
| 3 | MB | HD | M | 15 | 1 |
| 3 | MB | HD | M | 15 | 1 |
| 3 | MB | HD | M | 16 | 1 |
| 3 | MB | HD | M | 16 | 1 |
| 3 | MB | HD | M | 17 | 1 |
| 3 | MB | HD | M | 18 | 1 |
| 3 | MB | HD | M | 18 | 1 |
| 3 | MB | HD | M | 18 | 1 |
| 3 | MB | HD | M | 18 | 1 |
| 3 | MB | HD | M | 18 | 1 |
| 3 | MB | HD | M | 18 | 1 |
| 3 | MB | HD | M | 18 | 1 |
| 3 | MB | HD | M | 18 | 1 |
| 3 | MB | HD | M | 18 | 1 |
| 3 | MB | HD | M | 18 | 1 |
| 3 | MB | HD | M | 18 | 1 |
| 3 | MB | HD | M | 19 | 1 |
| 3 | MB | HD | M | 20 | 1 |
| 3 | MB | HD | M | 93 | 0 |
| 3 | MB | HD | M | 93 | 0 |
| 3 | MB | HD | M | 93 | 0 |
| 3 | MB | HD | M | 93 | 0 |
| 3 | MB | HD | M | 93 | 0 |
| 3 | MB | HD | M | 93 | 0 |
| 3 | MB | HD | M | 93 | 0 |
| 3 | MB | HD | M | 93 | 0 |
| 3 | MB | HD | M | 93 | 0 |
| 3 | MB | HD | M | 93 | 0 |
| 3 | MB | HD | M | 93 | 0 |
| 3 | MB | HD | M | 93 | 0 |
| 3 | MB | HD | M | 93 | 0 |
| 3 | MB | HD | M | 93 | 0 |
| 3 | MB | LD | M | 1 | 1 |
| 3 | MB | LD | M | 14 | 1 |
| 3 | MB | LD | M | 15 | 1 |
| 3 | MB | LD | M | 17 | 1 |
| 3 | MB | LD | M | 18 | 1 |
| 3 | MB | LD | M | 18 | 1 |
| 3 | MB | LD | M | 19 | 1 |
| 3 | MB | LD | M | 19 | 1 |
| 3 | MB | LD | M | 20 | 1 |
| 3 | MB | LD | M | 20 | 1 |
| 3 | MB | LD | M | 21 | 1 |
| 3 | MB | LD | M | 21 | 1 |
| 3 | MB | LD | M | 21 | 1 |
| 3 | MB | LD | M | 22 | 1 |
| 3 | MB | LD | M | 22 | 1 |
| 3 | MB | LD | M | 23 | 1 |
| 3 | MB | LD | M | 23 | 1 |
| 3 | MB | LD | M | 23 | 1 |
| 3 | MB | LD | M | 24 | 1 |
| 3 | MB | LD | M | 27 | 1 |
| 3 | MB | LD | M | 27 | 1 |
| 3 | MB | LD | M | 29 | 1 |
| 3 | MB | LD | M | 31 | 1 |
| 3 | MB | LD | M | 31 | 1 |
| 3 | MB | LD | M | 31 | 1 |
| 3 | MB | LD | M | 37 | 1 |
| 3 | MB | LD | M | 37 | 1 |
| 3 | MB | LD | M | 47 | 1 |
| 3 | MB | LD | M | 47 | 1 |
| 3 | MB | LD | M | 48 | 1 |
| 3 | MB | LD | M | 52 | 1 |
| 3 | MB | LD | M | 54 | 1 |
| 3 | MB | LD | M | 54 | 1 |
| 3 | MB | LD | M | 59 | 1 |
| 3 | MB | LD | M | 66 | 1 |
| 3 | MB | LD | M | 75 | 1 |
| 3 | MB | LD | M | 84 | 1 |
| 3 | MB | LD | M | 93 | 0 |
| 3 | MB | LD | M | 93 | 0 |
| 3 | MB | LD | M | 93 | 0 |
| 3 | MB | LD | M | 93 | 0 |
| 3 | MB | LD | M | 93 | 0 |
| 3 | MB | LD | M | 93 | 0 |
| 3 | MB | LD | M | 93 | 0 |
| 3 | MB | LD | M | 93 | 0 |
| 3 | MB | LD | M | 93 | 0 |
| 3 | MB | LD | M | 93 | 0 |
| 3 | MB | LD | M | 93 | 0 |
| 3 | MB | LD | M | 93 | 0 |
| 3 | MB | LD | M | 93 | 0 |
| 4 | MB | HD | M | 1 | 1 |
| 4 | MB | HD | M | 1 | 1 |
| 4 | MB | HD | M | 1 | 1 |
| 4 | MB | HD | M | 3 | 1 |
| 4 | MB | HD | M | 3 | 1 |
| 4 | MB | HD | M | 4 | 1 |
| 4 | MB | HD | M | 13 | 1 |
| 4 | MB | HD | M | 13 | 1 |
| 4 | MB | HD | M | 13 | 1 |
| 4 | MB | HD | M | 13 | 1 |
| 4 | MB | HD | M | 14 | 1 |
| 4 | MB | HD | M | 14 | 1 |
| 4 | MB | HD | M | 14 | 1 |
| 4 | MB | HD | M | 14 | 1 |
| 4 | MB | HD | M | 14 | 1 |
| 4 | MB | HD | M | 15 | 1 |
| 4 | MB | HD | M | 15 | 1 |
| 4 | MB | HD | M | 15 | 1 |
| 4 | MB | HD | M | 15 | 1 |
| 4 | MB | HD | M | 16 | 1 |
| 4 | MB | HD | M | 16 | 1 |
| 4 | MB | HD | M | 16 | 1 |
| 4 | MB | HD | M | 17 | 1 |
| 4 | MB | HD | M | 17 | 1 |
| 4 | MB | HD | M | 17 | 1 |
| 4 | MB | HD | M | 17 | 1 |
| 4 | MB | HD | M | 18 | 1 |
| 4 | MB | HD | M | 18 | 1 |
| 4 | MB | HD | M | 19 | 1 |
| 4 | MB | HD | M | 20 | 1 |
| 4 | MB | HD | M | 21 | 1 |
| 4 | MB | HD | M | 21 | 1 |
| 4 | MB | HD | M | 21 | 1 |
| 4 | MB | HD | M | 22 | 1 |
| 4 | MB | HD | M | 22 | 1 |
| 4 | MB | HD | M | 23 | 1 |
| 4 | MB | HD | M | 24 | 1 |
| 4 | MB | HD | M | 24 | 1 |
| 4 | MB | HD | M | 30 | 1 |
| 4 | MB | HD | M | 72 | 1 |
| 4 | MB | HD | M | 92 | 0 |
| 4 | MB | HD | M | 92 | 0 |
| 4 | MB | HD | M | 92 | 0 |
| 4 | MB | HD | M | 92 | 0 |
| 4 | MB | HD | M | 92 | 0 |
| 4 | MB | HD | M | 92 | 0 |
| 4 | MB | HD | M | 92 | 0 |
| 4 | MB | HD | M | 92 | 0 |
| 4 | MB | LD | M | 14 | 1 |
| 4 | MB | LD | M | 14 | 1 |
| 4 | MB | LD | M | 14 | 1 |
| 4 | MB | LD | M | 15 | 1 |
| 4 | MB | LD | M | 15 | 1 |
| 4 | MB | LD | M | 16 | 1 |
| 4 | MB | LD | M | 17 | 1 |
| 4 | MB | LD | M | 19 | 1 |
| 4 | MB | LD | M | 19 | 1 |
| 4 | MB | LD | M | 19 | 1 |
| 4 | MB | LD | M | 19 | 1 |
| 4 | MB | LD | M | 20 | 1 |
| 4 | MB | LD | M | 20 | 1 |
| 4 | MB | LD | M | 21 | 1 |
| 4 | MB | LD | M | 21 | 1 |
| 4 | MB | LD | M | 21 | 1 |
| 4 | MB | LD | M | 21 | 1 |
| 4 | MB | LD | M | 22 | 1 |
| 4 | MB | LD | M | 22 | 1 |
| 4 | MB | LD | M | 22 | 1 |
| 4 | MB | LD | M | 22 | 1 |
| 4 | MB | LD | M | 23 | 1 |
| 4 | MB | LD | M | 23 | 1 |
| 4 | MB | LD | M | 23 | 1 |
| 4 | MB | LD | M | 23 | 1 |
| 4 | MB | LD | M | 24 | 1 |
| 4 | MB | LD | M | 25 | 1 |
| 4 | MB | LD | M | 25 | 1 |
| 4 | MB | LD | M | 26 | 1 |
| 4 | MB | LD | M | 30 | 1 |
| 4 | MB | LD | M | 30 | 1 |
| 4 | MB | LD | M | 30 | 1 |
| 4 | MB | LD | M | 34 | 1 |
| 4 | MB | LD | M | 35 | 1 |
| 4 | MB | LD | M | 44 | 1 |
| 4 | MB | LD | M | 53 | 1 |
| 4 | MB | LD | M | 53 | 1 |
| 4 | MB | LD | M | 59 | 1 |
| 4 | MB | LD | M | 62 | 1 |
| 4 | MB | LD | M | 78 | 1 |
| 4 | MB | LD | M | 87 | 1 |
| 4 | MB | LD | M | 87 | 1 |
| 4 | MB | LD | M | 87 | 1 |
| 4 | MB | LD | M | 92 | 0 |
| 4 | MB | LD | M | 92 | 0 |
| 4 | MB | LD | M | 92 | 0 |
| 4 | MB | LD | M | 92 | 0 |
| 4 | MB | LD | M | 92 | 0 |
