# Supplementary material for: Evolution of pathogen-specific improved survivorship post-infection in populations of Drosophila melanogaster adapted to larval crowding
Source: PLoS One. 2021 Apr 14;16(4):e0250055. doi: 10.1371/journal.pone.0250055 (PMC8046209; doi:10.1371/journal.pone.0250055)
Supplement: S11 Table — (DOCX) [file pone.0250055.s011.docx]

**S11 Table: Survivorship data against *Enterococcus faecalis* bacteria with time of death in hours post infection**

| Block | Selection | Treatment | Sex | Time | Censor |
| --- | --- | --- | --- | --- | --- |
| 1 | MCU | HD | F | 20 | 1 |
| 1 | MCU | HD | F | 20 | 1 |
| 1 | MCU | HD | F | 21 | 1 |
| 1 | MCU | HD | F | 21 | 1 |
| 1 | MCU | HD | F | 22 | 1 |
| 1 | MCU | HD | F | 23 | 1 |
| 1 | MCU | HD | F | 23 | 1 |
| 1 | MCU | HD | F | 24 | 1 |
| 1 | MCU | HD | F | 24 | 1 |
| 1 | MCU | HD | F | 25 | 1 |
| 1 | MCU | HD | F | 25 | 1 |
| 1 | MCU | HD | F | 25 | 1 |
| 1 | MCU | HD | F | 25 | 1 |
| 1 | MCU | HD | F | 30 | 1 |
| 1 | MCU | HD | F | 31 | 1 |
| 1 | MCU | HD | F | 38 | 1 |
| 1 | MCU | HD | F | 38 | 1 |
| 1 | MCU | HD | F | 38 | 1 |
| 1 | MCU | HD | F | 43 | 1 |
| 1 | MCU | HD | F | 44 | 1 |
| 1 | MCU | HD | F | 72 | 0 |
| 1 | MCU | HD | F | 72 | 0 |
| 1 | MCU | HD | F | 72 | 0 |
| 1 | MCU | HD | F | 72 | 0 |
| 1 | MCU | HD | F | 72 | 0 |
| 1 | MCU | HD | F | 72 | 0 |
| 1 | MCU | HD | F | 72 | 0 |
| 1 | MCU | HD | F | 72 | 0 |
| 1 | MCU | HD | F | 72 | 0 |
| 1 | MCU | HD | F | 72 | 0 |
| 1 | MCU | HD | F | 72 | 0 |
| 1 | MCU | HD | F | 72 | 0 |
| 1 | MCU | HD | F | 72 | 0 |
| 1 | MCU | HD | F | 72 | 0 |
| 1 | MCU | HD | F | 72 | 0 |
| 1 | MCU | HD | F | 72 | 0 |
| 1 | MCU | HD | F | 72 | 0 |
| 1 | MCU | HD | F | 72 | 0 |
| 1 | MCU | HD | F | 72 | 0 |
| 1 | MCU | HD | F | 72 | 0 |
| 1 | MCU | HD | F | 72 | 0 |
| 1 | MCU | HD | F | 72 | 0 |
| 1 | MCU | HD | F | 72 | 0 |
| 1 | MCU | HD | F | 72 | 0 |
| 1 | MCU | HD | F | 72 | 0 |
| 1 | MCU | HD | F | 72 | 0 |
| 1 | MCU | HD | F | 72 | 0 |
| 1 | MCU | HD | F | 72 | 0 |
| 1 | MCU | HD | F | 72 | 0 |
| 1 | MCU | HD | F | 72 | 0 |
| 1 | MCU | HD | M | 19 | 1 |
| 1 | MCU | HD | M | 20 | 1 |
| 1 | MCU | HD | M | 20 | 1 |
| 1 | MCU | HD | M | 22 | 1 |
| 1 | MCU | HD | M | 22 | 1 |
| 1 | MCU | HD | M | 23 | 1 |
| 1 | MCU | HD | M | 25 | 1 |
| 1 | MCU | HD | M | 25 | 1 |
| 1 | MCU | HD | M | 25 | 1 |
| 1 | MCU | HD | M | 28 | 1 |
| 1 | MCU | HD | M | 30 | 1 |
| 1 | MCU | HD | M | 31 | 1 |
| 1 | MCU | HD | M | 31 | 1 |
| 1 | MCU | HD | M | 35 | 1 |
| 1 | MCU | HD | M | 35 | 1 |
| 1 | MCU | HD | M | 45 | 1 |
| 1 | MCU | HD | M | 72 | 0 |
| 1 | MCU | HD | M | 72 | 0 |
| 1 | MCU | HD | M | 72 | 0 |
| 1 | MCU | HD | M | 72 | 0 |
| 1 | MCU | HD | M | 72 | 0 |
| 1 | MCU | HD | M | 72 | 0 |
| 1 | MCU | HD | M | 72 | 0 |
| 1 | MCU | HD | M | 72 | 0 |
| 1 | MCU | HD | M | 72 | 0 |
| 1 | MCU | HD | M | 72 | 0 |
| 1 | MCU | HD | M | 72 | 0 |
| 1 | MCU | HD | M | 72 | 0 |
| 1 | MCU | HD | M | 72 | 0 |
| 1 | MCU | HD | M | 72 | 0 |
| 1 | MCU | HD | M | 72 | 0 |
| 1 | MCU | HD | M | 72 | 0 |
| 1 | MCU | HD | M | 72 | 0 |
| 1 | MCU | HD | M | 72 | 0 |
| 1 | MCU | HD | M | 72 | 0 |
| 1 | MCU | HD | M | 72 | 0 |
| 1 | MCU | HD | M | 72 | 0 |
| 1 | MCU | HD | M | 72 | 0 |
| 1 | MCU | HD | M | 72 | 0 |
| 1 | MCU | HD | M | 72 | 0 |
| 1 | MCU | HD | M | 72 | 0 |
| 1 | MCU | HD | M | 72 | 0 |
| 1 | MCU | HD | M | 72 | 0 |
| 1 | MCU | HD | M | 72 | 0 |
| 1 | MCU | HD | M | 72 | 0 |
| 1 | MCU | HD | M | 72 | 0 |
| 1 | MCU | HD | M | 72 | 0 |
| 1 | MCU | HD | M | 72 | 0 |
| 1 | MCU | HD | M | 72 | 0 |
| 1 | MCU | HD | M | 72 | 0 |
| 2 | MCU | HD | F | 4 | 1 |
| 2 | MCU | HD | F | 9 | 1 |
| 2 | MCU | HD | F | 17.5 | 1 |
| 2 | MCU | HD | F | 19 | 1 |
| 2 | MCU | HD | F | 19 | 1 |
| 2 | MCU | HD | F | 19 | 1 |
| 2 | MCU | HD | F | 19 | 1 |
| 2 | MCU | HD | F | 19 | 1 |
| 2 | MCU | HD | F | 19 | 1 |
| 2 | MCU | HD | F | 19 | 1 |
| 2 | MCU | HD | F | 19 | 1 |
| 2 | MCU | HD | F | 20 | 1 |
| 2 | MCU | HD | F | 20 | 1 |
| 2 | MCU | HD | F | 20 | 1 |
| 2 | MCU | HD | F | 21 | 1 |
| 2 | MCU | HD | F | 21 | 1 |
| 2 | MCU | HD | F | 21 | 1 |
| 2 | MCU | HD | F | 21 | 1 |
| 2 | MCU | HD | F | 21 | 1 |
| 2 | MCU | HD | F | 21 | 1 |
| 2 | MCU | HD | F | 22 | 1 |
| 2 | MCU | HD | F | 23 | 1 |
| 2 | MCU | HD | F | 23 | 1 |
| 2 | MCU | HD | F | 23 | 1 |
| 2 | MCU | HD | F | 24 | 1 |
| 2 | MCU | HD | F | 25 | 1 |
| 2 | MCU | HD | F | 26 | 1 |
| 2 | MCU | HD | F | 26 | 1 |
| 2 | MCU | HD | F | 30 | 1 |
| 2 | MCU | HD | F | 33 | 1 |
| 2 | MCU | HD | F | 68 | 1 |
| 2 | MCU | HD | F | 72 | 0 |
| 2 | MCU | HD | F | 72 | 0 |
| 2 | MCU | HD | F | 72 | 0 |
| 2 | MCU | HD | F | 72 | 0 |
| 2 | MCU | HD | F | 72 | 0 |
| 2 | MCU | HD | F | 72 | 0 |
| 2 | MCU | HD | F | 72 | 0 |
| 2 | MCU | HD | F | 72 | 0 |
| 2 | MCU | HD | F | 72 | 0 |
| 2 | MCU | HD | F | 72 | 0 |
| 2 | MCU | HD | F | 72 | 0 |
| 2 | MCU | HD | F | 72 | 0 |
| 2 | MCU | HD | F | 72 | 0 |
| 2 | MCU | HD | F | 72 | 0 |
| 2 | MCU | HD | F | 72 | 0 |
| 2 | MCU | HD | F | 72 | 0 |
| 2 | MCU | HD | F | 72 | 0 |
| 2 | MCU | HD | F | 72 | 0 |
| 2 | MCU | HD | F | 72 | 0 |
| 2 | MCU | HD | M | 4 | 1 |
| 2 | MCU | HD | M | 4 | 1 |
| 2 | MCU | HD | M | 9 | 1 |
| 2 | MCU | HD | M | 9 | 1 |
| 2 | MCU | HD | M | 17.5 | 1 |
| 2 | MCU | HD | M | 17.5 | 1 |
| 2 | MCU | HD | M | 17.5 | 1 |
| 2 | MCU | HD | M | 17.5 | 1 |
| 2 | MCU | HD | M | 17.5 | 1 |
| 2 | MCU | HD | M | 17.5 | 1 |
| 2 | MCU | HD | M | 17.5 | 1 |
| 2 | MCU | HD | M | 17.5 | 1 |
| 2 | MCU | HD | M | 19 | 1 |
| 2 | MCU | HD | M | 19 | 1 |
| 2 | MCU | HD | M | 19 | 1 |
| 2 | MCU | HD | M | 19 | 1 |
| 2 | MCU | HD | M | 20 | 1 |
| 2 | MCU | HD | M | 20 | 1 |
| 2 | MCU | HD | M | 20 | 1 |
| 2 | MCU | HD | M | 21 | 1 |
| 2 | MCU | HD | M | 21 | 1 |
| 2 | MCU | HD | M | 21 | 1 |
| 2 | MCU | HD | M | 21 | 1 |
| 2 | MCU | HD | M | 21 | 1 |
| 2 | MCU | HD | M | 22 | 1 |
| 2 | MCU | HD | M | 22 | 1 |
| 2 | MCU | HD | M | 25 | 1 |
| 2 | MCU | HD | M | 25 | 1 |
| 2 | MCU | HD | M | 25 | 1 |
| 2 | MCU | HD | M | 26 | 1 |
| 2 | MCU | HD | M | 26 | 1 |
| 2 | MCU | HD | M | 26 | 1 |
| 2 | MCU | HD | M | 27 | 1 |
| 2 | MCU | HD | M | 27 | 1 |
| 2 | MCU | HD | M | 29 | 1 |
| 2 | MCU | HD | M | 29 | 1 |
| 2 | MCU | HD | M | 39 | 1 |
| 2 | MCU | HD | M | 72 | 0 |
| 2 | MCU | HD | M | 72 | 0 |
| 2 | MCU | HD | M | 72 | 0 |
| 2 | MCU | HD | M | 72 | 0 |
| 2 | MCU | HD | M | 72 | 0 |
| 2 | MCU | HD | M | 72 | 0 |
| 2 | MCU | HD | M | 72 | 0 |
| 2 | MCU | HD | M | 72 | 0 |
| 2 | MCU | HD | M | 72 | 0 |
| 2 | MCU | HD | M | 72 | 0 |
| 2 | MCU | HD | M | 72 | 0 |
| 2 | MCU | HD | M | 72 | 0 |
| 2 | MCU | HD | M | 72 | 0 |
| 3 | MCU | HD | F | 16 | 1 |
| 3 | MCU | HD | F | 17 | 1 |
| 3 | MCU | HD | F | 17 | 1 |
| 3 | MCU | HD | F | 18 | 1 |
| 3 | MCU | HD | F | 18 | 1 |
| 3 | MCU | HD | F | 18 | 1 |
| 3 | MCU | HD | F | 19 | 1 |
| 3 | MCU | HD | F | 19 | 1 |
| 3 | MCU | HD | F | 19 | 1 |
| 3 | MCU | HD | F | 19 | 1 |
| 3 | MCU | HD | F | 20 | 1 |
| 3 | MCU | HD | F | 21 | 1 |
| 3 | MCU | HD | F | 21 | 1 |
| 3 | MCU | HD | F | 21 | 1 |
| 3 | MCU | HD | F | 21 | 1 |
| 3 | MCU | HD | F | 21 | 1 |
| 3 | MCU | HD | F | 22 | 1 |
| 3 | MCU | HD | F | 23 | 1 |
| 3 | MCU | HD | F | 23 | 1 |
| 3 | MCU | HD | F | 23 | 1 |
| 3 | MCU | HD | F | 25 | 1 |
| 3 | MCU | HD | F | 25 | 1 |
| 3 | MCU | HD | F | 28 | 1 |
| 3 | MCU | HD | F | 29 | 1 |
| 3 | MCU | HD | F | 29 | 1 |
| 3 | MCU | HD | F | 30 | 1 |
| 3 | MCU | HD | F | 31 | 1 |
| 3 | MCU | HD | F | 35 | 1 |
| 3 | MCU | HD | F | 36 | 1 |
| 3 | MCU | HD | F | 50.5 | 1 |
| 3 | MCU | HD | F | 90 | 0 |
| 3 | MCU | HD | F | 90 | 0 |
| 3 | MCU | HD | F | 90 | 0 |
| 3 | MCU | HD | F | 90 | 0 |
| 3 | MCU | HD | F | 90 | 0 |
| 3 | MCU | HD | F | 90 | 0 |
| 3 | MCU | HD | F | 90 | 0 |
| 3 | MCU | HD | F | 90 | 0 |
| 3 | MCU | HD | F | 90 | 0 |
| 3 | MCU | HD | F | 90 | 0 |
| 3 | MCU | HD | F | 90 | 0 |
| 3 | MCU | HD | F | 90 | 0 |
| 3 | MCU | HD | F | 90 | 0 |
| 3 | MCU | HD | F | 90 | 0 |
| 3 | MCU | HD | F | 90 | 0 |
| 3 | MCU | HD | F | 90 | 0 |
| 3 | MCU | HD | F | 90 | 0 |
| 3 | MCU | HD | F | 90 | 0 |
| 3 | MCU | HD | F | 90 | 0 |
| 3 | MCU | HD | F | 90 | 0 |
| 3 | MCU | HD | M | 0 | 1 |
| 3 | MCU | HD | M | 16 | 1 |
| 3 | MCU | HD | M | 16 | 1 |
| 3 | MCU | HD | M | 16 | 1 |
| 3 | MCU | HD | M | 16 | 1 |
| 3 | MCU | HD | M | 18 | 1 |
| 3 | MCU | HD | M | 19 | 1 |
| 3 | MCU | HD | M | 19 | 1 |
| 3 | MCU | HD | M | 19 | 1 |
| 3 | MCU | HD | M | 19 | 1 |
| 3 | MCU | HD | M | 19 | 1 |
| 3 | MCU | HD | M | 20 | 1 |
| 3 | MCU | HD | M | 20 | 1 |
| 3 | MCU | HD | M | 20 | 1 |
| 3 | MCU | HD | M | 21 | 1 |
| 3 | MCU | HD | M | 22 | 1 |
| 3 | MCU | HD | M | 23 | 1 |
| 3 | MCU | HD | M | 24 | 1 |
| 3 | MCU | HD | M | 24 | 1 |
| 3 | MCU | HD | M | 24 | 1 |
| 3 | MCU | HD | M | 25 | 1 |
| 3 | MCU | HD | M | 28 | 1 |
| 3 | MCU | HD | M | 34 | 1 |
| 3 | MCU | HD | M | 35 | 1 |
| 3 | MCU | HD | M | 90 | 0 |
| 3 | MCU | HD | M | 90 | 0 |
| 3 | MCU | HD | M | 90 | 0 |
| 3 | MCU | HD | M | 90 | 0 |
| 3 | MCU | HD | M | 90 | 0 |
| 3 | MCU | HD | M | 90 | 0 |
| 3 | MCU | HD | M | 90 | 0 |
| 3 | MCU | HD | M | 90 | 0 |
| 3 | MCU | HD | M | 90 | 0 |
| 3 | MCU | HD | M | 90 | 0 |
| 3 | MCU | HD | M | 90 | 0 |
| 3 | MCU | HD | M | 90 | 0 |
| 3 | MCU | HD | M | 90 | 0 |
| 3 | MCU | HD | M | 90 | 0 |
| 3 | MCU | HD | M | 90 | 0 |
| 3 | MCU | HD | M | 90 | 0 |
| 3 | MCU | HD | M | 90 | 0 |
| 3 | MCU | HD | M | 90 | 0 |
| 3 | MCU | HD | M | 90 | 0 |
| 3 | MCU | HD | M | 90 | 0 |
| 3 | MCU | HD | M | 90 | 0 |
| 3 | MCU | HD | M | 90 | 0 |
| 3 | MCU | HD | M | 90 | 0 |
| 3 | MCU | HD | M | 90 | 0 |
| 3 | MCU | HD | M | 90 | 0 |
| 3 | MCU | HD | M | 90 | 0 |
| 4 | MCU | HD | F | 5 | 1 |
| 4 | MCU | HD | F | 17 | 1 |
| 4 | MCU | HD | F | 18 | 1 |
| 4 | MCU | HD | F | 18 | 1 |
| 4 | MCU | HD | F | 19 | 1 |
| 4 | MCU | HD | F | 19 | 1 |
| 4 | MCU | HD | F | 19 | 1 |
| 4 | MCU | HD | F | 20 | 1 |
| 4 | MCU | HD | F | 20 | 1 |
| 4 | MCU | HD | F | 20 | 1 |
| 4 | MCU | HD | F | 21 | 1 |
| 4 | MCU | HD | F | 22 | 1 |
| 4 | MCU | HD | F | 22 | 1 |
| 4 | MCU | HD | F | 23 | 1 |
| 4 | MCU | HD | F | 23 | 1 |
| 4 | MCU | HD | F | 23 | 1 |
| 4 | MCU | HD | F | 24 | 1 |
| 4 | MCU | HD | F | 24 | 1 |
| 4 | MCU | HD | F | 25 | 1 |
| 4 | MCU | HD | F | 26 | 1 |
| 4 | MCU | HD | F | 26 | 1 |
| 4 | MCU | HD | F | 26 | 1 |
| 4 | MCU | HD | F | 30 | 1 |
| 4 | MCU | HD | F | 31 | 1 |
| 4 | MCU | HD | F | 31 | 1 |
| 4 | MCU | HD | F | 31 | 1 |
| 4 | MCU | HD | F | 31 | 1 |
| 4 | MCU | HD | F | 38 | 1 |
| 4 | MCU | HD | F | 93 | 0 |
| 4 | MCU | HD | F | 93 | 0 |
| 4 | MCU | HD | F | 93 | 0 |
| 4 | MCU | HD | F | 93 | 0 |
| 4 | MCU | HD | F | 93 | 0 |
| 4 | MCU | HD | F | 93 | 0 |
| 4 | MCU | HD | F | 93 | 0 |
| 4 | MCU | HD | F | 93 | 0 |
| 4 | MCU | HD | F | 93 | 0 |
| 4 | MCU | HD | F | 93 | 0 |
| 4 | MCU | HD | F | 93 | 0 |
| 4 | MCU | HD | F | 93 | 0 |
| 4 | MCU | HD | F | 93 | 0 |
| 4 | MCU | HD | F | 93 | 0 |
| 4 | MCU | HD | F | 93 | 0 |
| 4 | MCU | HD | F | 93 | 0 |
| 4 | MCU | HD | F | 93 | 0 |
| 4 | MCU | HD | F | 93 | 0 |
| 4 | MCU | HD | F | 93 | 0 |
| 4 | MCU | HD | F | 93 | 0 |
| 4 | MCU | HD | F | 93 | 0 |
| 4 | MCU | HD | F | 93 | 0 |
| 4 | MCU | HD | M | 0 | 1 |
| 4 | MCU | HD | M | 0 | 1 |
| 4 | MCU | HD | M | 5 | 1 |
| 4 | MCU | HD | M | 18 | 1 |
| 4 | MCU | HD | M | 18 | 1 |
| 4 | MCU | HD | M | 18 | 1 |
| 4 | MCU | HD | M | 18 | 1 |
| 4 | MCU | HD | M | 20 | 1 |
| 4 | MCU | HD | M | 20 | 1 |
| 4 | MCU | HD | M | 21 | 1 |
| 4 | MCU | HD | M | 22 | 1 |
| 4 | MCU | HD | M | 23 | 1 |
| 4 | MCU | HD | M | 23 | 1 |
| 4 | MCU | HD | M | 24 | 1 |
| 4 | MCU | HD | M | 24 | 1 |
| 4 | MCU | HD | M | 25 | 1 |
| 4 | MCU | HD | M | 26 | 1 |
| 4 | MCU | HD | M | 26 | 1 |
| 4 | MCU | HD | M | 29 | 1 |
| 4 | MCU | HD | M | 32 | 1 |
| 4 | MCU | HD | M | 37 | 1 |
| 4 | MCU | HD | M | 93 | 0 |
| 4 | MCU | HD | M | 93 | 0 |
| 4 | MCU | HD | M | 93 | 0 |
| 4 | MCU | HD | M | 93 | 0 |
| 4 | MCU | HD | M | 93 | 0 |
| 4 | MCU | HD | M | 93 | 0 |
| 4 | MCU | HD | M | 93 | 0 |
| 4 | MCU | HD | M | 93 | 0 |
| 4 | MCU | HD | M | 93 | 0 |
| 4 | MCU | HD | M | 93 | 0 |
| 4 | MCU | HD | M | 93 | 0 |
| 4 | MCU | HD | M | 93 | 0 |
| 4 | MCU | HD | M | 93 | 0 |
| 4 | MCU | HD | M | 93 | 0 |
| 4 | MCU | HD | M | 93 | 0 |
| 4 | MCU | HD | M | 93 | 0 |
| 4 | MCU | HD | M | 93 | 0 |
| 4 | MCU | HD | M | 93 | 0 |
| 4 | MCU | HD | M | 93 | 0 |
| 4 | MCU | HD | M | 93 | 0 |
| 4 | MCU | HD | M | 93 | 0 |
| 4 | MCU | HD | M | 93 | 0 |
| 4 | MCU | HD | M | 93 | 0 |
| 4 | MCU | HD | M | 93 | 0 |
| 4 | MCU | HD | M | 93 | 0 |
| 4 | MCU | HD | M | 93 | 0 |
| 4 | MCU | HD | M | 93 | 0 |
| 4 | MCU | HD | M | 93 | 0 |
| 4 | MCU | HD | M | 93 | 0 |
| 1 | MCU | LD | F | 21 | 1 |
| 1 | MCU | LD | F | 21 | 1 |
| 1 | MCU | LD | F | 21 | 1 |
| 1 | MCU | LD | F | 22 | 1 |
| 1 | MCU | LD | F | 22 | 1 |
| 1 | MCU | LD | F | 22 | 1 |
| 1 | MCU | LD | F | 22 | 1 |
| 1 | MCU | LD | F | 23 | 1 |
| 1 | MCU | LD | F | 23 | 1 |
| 1 | MCU | LD | F | 24 | 1 |
| 1 | MCU | LD | F | 24 | 1 |
| 1 | MCU | LD | F | 25 | 1 |
| 1 | MCU | LD | F | 26 | 1 |
| 1 | MCU | LD | F | 26 | 1 |
| 1 | MCU | LD | F | 26 | 1 |
| 1 | MCU | LD | F | 26 | 1 |
| 1 | MCU | LD | F | 26 | 1 |
| 1 | MCU | LD | F | 27 | 1 |
| 1 | MCU | LD | F | 27 | 1 |
| 1 | MCU | LD | F | 27 | 1 |
| 1 | MCU | LD | F | 28 | 1 |
| 1 | MCU | LD | F | 28 | 1 |
| 1 | MCU | LD | F | 33 | 1 |
| 1 | MCU | LD | F | 34 | 1 |
| 1 | MCU | LD | F | 43 | 1 |
| 1 | MCU | LD | F | 44 | 1 |
| 1 | MCU | LD | F | 51 | 1 |
| 1 | MCU | LD | F | 72 | 0 |
| 1 | MCU | LD | F | 72 | 0 |
| 1 | MCU | LD | F | 72 | 0 |
| 1 | MCU | LD | F | 72 | 0 |
| 1 | MCU | LD | F | 72 | 0 |
| 1 | MCU | LD | F | 72 | 0 |
| 1 | MCU | LD | F | 72 | 0 |
| 1 | MCU | LD | F | 72 | 0 |
| 1 | MCU | LD | F | 72 | 0 |
| 1 | MCU | LD | F | 72 | 0 |
| 1 | MCU | LD | F | 72 | 0 |
| 1 | MCU | LD | F | 72 | 0 |
| 1 | MCU | LD | F | 72 | 0 |
| 1 | MCU | LD | F | 72 | 0 |
| 1 | MCU | LD | F | 72 | 0 |
| 1 | MCU | LD | F | 72 | 0 |
| 1 | MCU | LD | F | 72 | 0 |
| 1 | MCU | LD | F | 72 | 0 |
| 1 | MCU | LD | F | 72 | 0 |
| 1 | MCU | LD | F | 72 | 0 |
| 1 | MCU | LD | F | 72 | 0 |
| 1 | MCU | LD | F | 72 | 0 |
| 1 | MCU | LD | F | 72 | 0 |
| 1 | MCU | LD | M | 18 | 1 |
| 1 | MCU | LD | M | 19 | 1 |
| 1 | MCU | LD | M | 21 | 1 |
| 1 | MCU | LD | M | 21 | 1 |
| 1 | MCU | LD | M | 21 | 1 |
| 1 | MCU | LD | M | 22 | 1 |
| 1 | MCU | LD | M | 22 | 1 |
| 1 | MCU | LD | M | 23 | 1 |
| 1 | MCU | LD | M | 23 | 1 |
| 1 | MCU | LD | M | 24 | 1 |
| 1 | MCU | LD | M | 24 | 1 |
| 1 | MCU | LD | M | 24 | 1 |
| 1 | MCU | LD | M | 25 | 1 |
| 1 | MCU | LD | M | 25 | 1 |
| 1 | MCU | LD | M | 25 | 1 |
| 1 | MCU | LD | M | 27 | 1 |
| 1 | MCU | LD | M | 29 | 1 |
| 1 | MCU | LD | M | 31 | 1 |
| 1 | MCU | LD | M | 33 | 1 |
| 1 | MCU | LD | M | 33 | 1 |
| 1 | MCU | LD | M | 35 | 1 |
| 1 | MCU | LD | M | 35 | 1 |
| 1 | MCU | LD | M | 44 | 1 |
| 1 | MCU | LD | M | 44 | 1 |
| 1 | MCU | LD | M | 44 | 1 |
| 1 | MCU | LD | M | 72 | 0 |
| 1 | MCU | LD | M | 72 | 0 |
| 1 | MCU | LD | M | 72 | 0 |
| 1 | MCU | LD | M | 72 | 0 |
| 1 | MCU | LD | M | 72 | 0 |
| 1 | MCU | LD | M | 72 | 0 |
| 1 | MCU | LD | M | 72 | 0 |
| 1 | MCU | LD | M | 72 | 0 |
| 1 | MCU | LD | M | 72 | 0 |
| 1 | MCU | LD | M | 72 | 0 |
| 1 | MCU | LD | M | 72 | 0 |
| 1 | MCU | LD | M | 72 | 0 |
| 1 | MCU | LD | M | 72 | 0 |
| 1 | MCU | LD | M | 72 | 0 |
| 1 | MCU | LD | M | 72 | 0 |
| 1 | MCU | LD | M | 72 | 0 |
| 1 | MCU | LD | M | 72 | 0 |
| 1 | MCU | LD | M | 72 | 0 |
| 1 | MCU | LD | M | 72 | 0 |
| 1 | MCU | LD | M | 72 | 0 |
| 1 | MCU | LD | M | 72 | 0 |
| 1 | MCU | LD | M | 72 | 0 |
| 1 | MCU | LD | M | 72 | 0 |
| 1 | MCU | LD | M | 72 | 0 |
| 1 | MCU | LD | M | 72 | 0 |
| 2 | MCU | LD | F | 15 | 1 |
| 2 | MCU | LD | F | 20 | 1 |
| 2 | MCU | LD | F | 20 | 1 |
| 2 | MCU | LD | F | 20 | 1 |
| 2 | MCU | LD | F | 21 | 1 |
| 2 | MCU | LD | F | 21 | 1 |
| 2 | MCU | LD | F | 21 | 1 |
| 2 | MCU | LD | F | 21 | 0 |
| 2 | MCU | LD | F | 23 | 1 |
| 2 | MCU | LD | F | 23 | 1 |
| 2 | MCU | LD | F | 23 | 1 |
| 2 | MCU | LD | F | 23 | 1 |
| 2 | MCU | LD | F | 24 | 1 |
| 2 | MCU | LD | F | 24 | 1 |
| 2 | MCU | LD | F | 24 | 1 |
| 2 | MCU | LD | F | 25 | 1 |
| 2 | MCU | LD | F | 25 | 1 |
| 2 | MCU | LD | F | 26 | 1 |
| 2 | MCU | LD | F | 26 | 1 |
| 2 | MCU | LD | F | 27 | 1 |
| 2 | MCU | LD | F | 28 | 1 |
| 2 | MCU | LD | F | 28 | 1 |
| 2 | MCU | LD | F | 28 | 1 |
| 2 | MCU | LD | F | 30 | 1 |
| 2 | MCU | LD | F | 32 | 1 |
| 2 | MCU | LD | F | 33 | 1 |
| 2 | MCU | LD | F | 35 | 1 |
| 2 | MCU | LD | F | 35 | 1 |
| 2 | MCU | LD | F | 35 | 1 |
| 2 | MCU | LD | F | 41.5 | 1 |
| 2 | MCU | LD | F | 44.5 | 1 |
| 2 | MCU | LD | F | 64 | 1 |
| 2 | MCU | LD | F | 72 | 0 |
| 2 | MCU | LD | F | 72 | 0 |
| 2 | MCU | LD | F | 72 | 0 |
| 2 | MCU | LD | F | 72 | 0 |
| 2 | MCU | LD | F | 72 | 0 |
| 2 | MCU | LD | F | 72 | 0 |
| 2 | MCU | LD | F | 72 | 0 |
| 2 | MCU | LD | F | 72 | 0 |
| 2 | MCU | LD | F | 72 | 0 |
| 2 | MCU | LD | F | 72 | 0 |
| 2 | MCU | LD | F | 72 | 0 |
| 2 | MCU | LD | F | 72 | 0 |
| 2 | MCU | LD | F | 72 | 0 |
| 2 | MCU | LD | F | 72 | 0 |
| 2 | MCU | LD | F | 72 | 0 |
| 2 | MCU | LD | F | 72 | 0 |
| 2 | MCU | LD | F | 72 | 0 |
| 2 | MCU | LD | F | 72 | 0 |
| 2 | MCU | LD | M | 4 | 1 |
| 2 | MCU | LD | M | 17.5 | 1 |
| 2 | MCU | LD | M | 19 | 1 |
| 2 | MCU | LD | M | 20 | 1 |
| 2 | MCU | LD | M | 20 | 1 |
| 2 | MCU | LD | M | 20 | 1 |
| 2 | MCU | LD | M | 20 | 1 |
| 2 | MCU | LD | M | 21 | 1 |
| 2 | MCU | LD | M | 21 | 1 |
| 2 | MCU | LD | M | 21 | 1 |
| 2 | MCU | LD | M | 21 | 1 |
| 2 | MCU | LD | M | 21 | 1 |
| 2 | MCU | LD | M | 22 | 1 |
| 2 | MCU | LD | M | 23 | 1 |
| 2 | MCU | LD | M | 23 | 1 |
| 2 | MCU | LD | M | 24 | 1 |
| 2 | MCU | LD | M | 24 | 1 |
| 2 | MCU | LD | M | 24 | 1 |
| 2 | MCU | LD | M | 25 | 1 |
| 2 | MCU | LD | M | 25 | 1 |
| 2 | MCU | LD | M | 30 | 1 |
| 2 | MCU | LD | M | 32 | 1 |
| 2 | MCU | LD | M | 34 | 1 |
| 2 | MCU | LD | M | 35 | 1 |
| 2 | MCU | LD | M | 35 | 1 |
| 2 | MCU | LD | M | 36 | 1 |
| 2 | MCU | LD | M | 38 | 1 |
| 2 | MCU | LD | M | 47 | 1 |
| 2 | MCU | LD | M | 72 | 0 |
| 2 | MCU | LD | M | 72 | 0 |
| 2 | MCU | LD | M | 72 | 0 |
| 2 | MCU | LD | M | 72 | 0 |
| 2 | MCU | LD | M | 72 | 0 |
| 2 | MCU | LD | M | 72 | 0 |
| 2 | MCU | LD | M | 72 | 0 |
| 2 | MCU | LD | M | 72 | 0 |
| 2 | MCU | LD | M | 72 | 0 |
| 2 | MCU | LD | M | 72 | 0 |
| 2 | MCU | LD | M | 72 | 0 |
| 2 | MCU | LD | M | 72 | 0 |
| 2 | MCU | LD | M | 72 | 0 |
| 2 | MCU | LD | M | 72 | 0 |
| 2 | MCU | LD | M | 72 | 0 |
| 2 | MCU | LD | M | 72 | 0 |
| 2 | MCU | LD | M | 72 | 0 |
| 2 | MCU | LD | M | 72 | 0 |
| 2 | MCU | LD | M | 72 | 0 |
| 2 | MCU | LD | M | 72 | 0 |
| 2 | MCU | LD | M | 72 | 0 |
| 2 | MCU | LD | M | 72 | 0 |
| 3 | MCU | LD | F | 5 | 1 |
| 3 | MCU | LD | F | 19 | 1 |
| 3 | MCU | LD | F | 21 | 1 |
| 3 | MCU | LD | F | 22 | 1 |
| 3 | MCU | LD | F | 22 | 1 |
| 3 | MCU | LD | F | 22 | 1 |
| 3 | MCU | LD | F | 23 | 1 |
| 3 | MCU | LD | F | 23 | 1 |
| 3 | MCU | LD | F | 24 | 1 |
| 3 | MCU | LD | F | 24 | 1 |
| 3 | MCU | LD | F | 24 | 1 |
| 3 | MCU | LD | F | 24 | 1 |
| 3 | MCU | LD | F | 24 | 1 |
| 3 | MCU | LD | F | 25 | 1 |
| 3 | MCU | LD | F | 25 | 1 |
| 3 | MCU | LD | F | 26 | 1 |
| 3 | MCU | LD | F | 26 | 1 |
| 3 | MCU | LD | F | 26 | 1 |
| 3 | MCU | LD | F | 30 | 1 |
| 3 | MCU | LD | F | 30 | 1 |
| 3 | MCU | LD | F | 33 | 1 |
| 3 | MCU | LD | F | 33 | 1 |
| 3 | MCU | LD | F | 34 | 1 |
| 3 | MCU | LD | F | 35 | 1 |
| 3 | MCU | LD | F | 35 | 1 |
| 3 | MCU | LD | F | 35 | 1 |
| 3 | MCU | LD | F | 40.5 | 1 |
| 3 | MCU | LD | F | 40.5 | 1 |
| 3 | MCU | LD | F | 45.5 | 1 |
| 3 | MCU | LD | F | 90 | 0 |
| 3 | MCU | LD | F | 90 | 0 |
| 3 | MCU | LD | F | 90 | 0 |
| 3 | MCU | LD | F | 90 | 0 |
| 3 | MCU | LD | F | 90 | 0 |
| 3 | MCU | LD | F | 90 | 0 |
| 3 | MCU | LD | F | 90 | 0 |
| 3 | MCU | LD | F | 90 | 0 |
| 3 | MCU | LD | F | 90 | 0 |
| 3 | MCU | LD | F | 90 | 0 |
| 3 | MCU | LD | F | 90 | 0 |
| 3 | MCU | LD | F | 90 | 0 |
| 3 | MCU | LD | F | 90 | 0 |
| 3 | MCU | LD | F | 90 | 0 |
| 3 | MCU | LD | F | 90 | 0 |
| 3 | MCU | LD | F | 90 | 0 |
| 3 | MCU | LD | F | 90 | 0 |
| 3 | MCU | LD | F | 90 | 0 |
| 3 | MCU | LD | F | 90 | 0 |
| 3 | MCU | LD | F | 90 | 0 |
| 3 | MCU | LD | F | 90 | 0 |
| 3 | MCU | LD | M | 18 | 1 |
| 3 | MCU | LD | M | 18 | 1 |
| 3 | MCU | LD | M | 20 | 1 |
| 3 | MCU | LD | M | 20 | 1 |
| 3 | MCU | LD | M | 21 | 1 |
| 3 | MCU | LD | M | 23 | 1 |
| 3 | MCU | LD | M | 23 | 1 |
| 3 | MCU | LD | M | 23 | 1 |
| 3 | MCU | LD | M | 23 | 1 |
| 3 | MCU | LD | M | 24 | 1 |
| 3 | MCU | LD | M | 24 | 1 |
| 3 | MCU | LD | M | 24 | 1 |
| 3 | MCU | LD | M | 26 | 1 |
| 3 | MCU | LD | M | 26 | 1 |
| 3 | MCU | LD | M | 26 | 1 |
| 3 | MCU | LD | M | 27 | 1 |
| 3 | MCU | LD | M | 29 | 1 |
| 3 | MCU | LD | M | 29 | 1 |
| 3 | MCU | LD | M | 29 | 1 |
| 3 | MCU | LD | M | 30 | 1 |
| 3 | MCU | LD | M | 30 | 1 |
| 3 | MCU | LD | M | 30 | 1 |
| 3 | MCU | LD | M | 35 | 1 |
| 3 | MCU | LD | M | 35 | 1 |
| 3 | MCU | LD | M | 90 | 0 |
| 3 | MCU | LD | M | 90 | 0 |
| 3 | MCU | LD | M | 90 | 0 |
| 3 | MCU | LD | M | 90 | 0 |
| 3 | MCU | LD | M | 90 | 0 |
| 3 | MCU | LD | M | 90 | 0 |
| 3 | MCU | LD | M | 90 | 0 |
| 3 | MCU | LD | M | 90 | 0 |
| 3 | MCU | LD | M | 90 | 0 |
| 3 | MCU | LD | M | 90 | 0 |
| 3 | MCU | LD | M | 90 | 0 |
| 3 | MCU | LD | M | 90 | 0 |
| 3 | MCU | LD | M | 90 | 0 |
| 3 | MCU | LD | M | 90 | 0 |
| 3 | MCU | LD | M | 90 | 0 |
| 3 | MCU | LD | M | 90 | 0 |
| 3 | MCU | LD | M | 90 | 0 |
| 3 | MCU | LD | M | 90 | 0 |
| 3 | MCU | LD | M | 90 | 0 |
| 3 | MCU | LD | M | 90 | 0 |
| 3 | MCU | LD | M | 90 | 0 |
| 3 | MCU | LD | M | 90 | 0 |
| 3 | MCU | LD | M | 90 | 0 |
| 3 | MCU | LD | M | 90 | 0 |
| 3 | MCU | LD | M | 90 | 0 |
| 3 | MCU | LD | M | 90 | 0 |
| 4 | MCU | LD | F | 17 | 1 |
| 4 | MCU | LD | F | 20 | 1 |
| 4 | MCU | LD | F | 20 | 1 |
| 4 | MCU | LD | F | 22 | 1 |
| 4 | MCU | LD | F | 22 | 1 |
| 4 | MCU | LD | F | 22 | 1 |
| 4 | MCU | LD | F | 22 | 1 |
| 4 | MCU | LD | F | 23 | 1 |
| 4 | MCU | LD | F | 24 | 1 |
| 4 | MCU | LD | F | 24 | 1 |
| 4 | MCU | LD | F | 24 | 1 |
| 4 | MCU | LD | F | 24 | 1 |
| 4 | MCU | LD | F | 25 | 1 |
| 4 | MCU | LD | F | 25 | 1 |
| 4 | MCU | LD | F | 26 | 1 |
| 4 | MCU | LD | F | 26 | 1 |
| 4 | MCU | LD | F | 29 | 1 |
| 4 | MCU | LD | F | 29 | 1 |
| 4 | MCU | LD | F | 30 | 1 |
| 4 | MCU | LD | F | 31 | 1 |
| 4 | MCU | LD | F | 36 | 1 |
| 4 | MCU | LD | F | 37 | 1 |
| 4 | MCU | LD | F | 40 | 1 |
| 4 | MCU | LD | F | 40 | 1 |
| 4 | MCU | LD | F | 40 | 1 |
| 4 | MCU | LD | F | 42.5 | 1 |
| 4 | MCU | LD | F | 93 | 0 |
| 4 | MCU | LD | F | 93 | 0 |
| 4 | MCU | LD | F | 93 | 0 |
| 4 | MCU | LD | F | 93 | 0 |
| 4 | MCU | LD | F | 93 | 0 |
| 4 | MCU | LD | F | 93 | 0 |
| 4 | MCU | LD | F | 93 | 0 |
| 4 | MCU | LD | F | 93 | 0 |
| 4 | MCU | LD | F | 93 | 0 |
| 4 | MCU | LD | F | 93 | 0 |
| 4 | MCU | LD | F | 93 | 0 |
| 4 | MCU | LD | F | 93 | 0 |
| 4 | MCU | LD | F | 93 | 0 |
| 4 | MCU | LD | F | 93 | 0 |
| 4 | MCU | LD | F | 93 | 0 |
| 4 | MCU | LD | F | 93 | 0 |
| 4 | MCU | LD | F | 93 | 0 |
| 4 | MCU | LD | F | 93 | 0 |
| 4 | MCU | LD | F | 93 | 0 |
| 4 | MCU | LD | F | 93 | 0 |
| 4 | MCU | LD | F | 93 | 0 |
| 4 | MCU | LD | F | 93 | 0 |
| 4 | MCU | LD | F | 93 | 0 |
| 4 | MCU | LD | F | 93 | 0 |
| 4 | MCU | LD | M | 19 | 1 |
| 4 | MCU | LD | M | 20 | 1 |
| 4 | MCU | LD | M | 20 | 1 |
| 4 | MCU | LD | M | 22 | 1 |
| 4 | MCU | LD | M | 22 | 1 |
| 4 | MCU | LD | M | 24 | 1 |
| 4 | MCU | LD | M | 24 | 1 |
| 4 | MCU | LD | M | 24 | 1 |
| 4 | MCU | LD | M | 24 | 1 |
| 4 | MCU | LD | M | 24 | 1 |
| 4 | MCU | LD | M | 24 | 1 |
| 4 | MCU | LD | M | 25 | 1 |
| 4 | MCU | LD | M | 26 | 1 |
| 4 | MCU | LD | M | 27 | 1 |
| 4 | MCU | LD | M | 27 | 1 |
| 4 | MCU | LD | M | 27 | 1 |
| 4 | MCU | LD | M | 27 | 1 |
| 4 | MCU | LD | M | 27 | 1 |
| 4 | MCU | LD | M | 27 | 1 |
| 4 | MCU | LD | M | 27 | 1 |
| 4 | MCU | LD | M | 27 | 1 |
| 4 | MCU | LD | M | 27 | 1 |
| 4 | MCU | LD | M | 27 | 1 |
| 4 | MCU | LD | M | 28 | 1 |
| 4 | MCU | LD | M | 28 | 1 |
| 4 | MCU | LD | M | 28 | 1 |
| 4 | MCU | LD | M | 28 | 1 |
| 4 | MCU | LD | M | 29 | 1 |
| 4 | MCU | LD | M | 32 | 1 |
| 4 | MCU | LD | M | 34 | 1 |
| 4 | MCU | LD | M | 37 | 1 |
| 4 | MCU | LD | M | 52.5 | 1 |
| 4 | MCU | LD | M | 52.5 | 1 |
| 4 | MCU | LD | M | 93 | 0 |
| 4 | MCU | LD | M | 93 | 0 |
| 4 | MCU | LD | M | 93 | 0 |
| 4 | MCU | LD | M | 93 | 0 |
| 4 | MCU | LD | M | 93 | 0 |
| 4 | MCU | LD | M | 93 | 0 |
| 4 | MCU | LD | M | 93 | 0 |
| 4 | MCU | LD | M | 93 | 0 |
| 4 | MCU | LD | M | 93 | 0 |
| 4 | MCU | LD | M | 93 | 0 |
| 4 | MCU | LD | M | 93 | 0 |
| 4 | MCU | LD | M | 93 | 0 |
| 4 | MCU | LD | M | 93 | 0 |
| 4 | MCU | LD | M | 93 | 0 |
| 4 | MCU | LD | M | 93 | 0 |
| 4 | MCU | LD | M | 93 | 0 |
| 4 | MCU | LD | M | 93 | 0 |
| 1 | MB | HD | F | 18 | 1 |
| 1 | MB | HD | F | 19 | 1 |
| 1 | MB | HD | F | 20 | 1 |
| 1 | MB | HD | F | 20 | 1 |
| 1 | MB | HD | F | 20 | 1 |
| 1 | MB | HD | F | 20 | 1 |
| 1 | MB | HD | F | 22 | 1 |
| 1 | MB | HD | F | 22 | 1 |
| 1 | MB | HD | F | 23 | 1 |
| 1 | MB | HD | F | 23 | 1 |
| 1 | MB | HD | F | 24 | 1 |
| 1 | MB | HD | F | 24 | 1 |
| 1 | MB | HD | F | 24 | 1 |
| 1 | MB | HD | F | 25 | 1 |
| 1 | MB | HD | F | 25 | 1 |
| 1 | MB | HD | F | 26 | 1 |
| 1 | MB | HD | F | 33 | 1 |
| 1 | MB | HD | F | 33 | 1 |
| 1 | MB | HD | F | 33 | 1 |
| 1 | MB | HD | F | 33 | 1 |
| 1 | MB | HD | F | 35 | 1 |
| 1 | MB | HD | F | 35 | 1 |
| 1 | MB | HD | F | 44 | 1 |
| 1 | MB | HD | F | 49 | 1 |
| 1 | MB | HD | F | 72 | 0 |
| 1 | MB | HD | F | 72 | 0 |
| 1 | MB | HD | F | 72 | 0 |
| 1 | MB | HD | F | 72 | 0 |
| 1 | MB | HD | F | 72 | 0 |
| 1 | MB | HD | F | 72 | 0 |
| 1 | MB | HD | F | 72 | 0 |
| 1 | MB | HD | F | 72 | 0 |
| 1 | MB | HD | F | 72 | 0 |
| 1 | MB | HD | F | 72 | 0 |
| 1 | MB | HD | F | 72 | 0 |
| 1 | MB | HD | F | 72 | 0 |
| 1 | MB | HD | F | 72 | 0 |
| 1 | MB | HD | F | 72 | 0 |
| 1 | MB | HD | F | 72 | 0 |
| 1 | MB | HD | F | 72 | 0 |
| 1 | MB | HD | F | 72 | 0 |
| 1 | MB | HD | F | 72 | 0 |
| 1 | MB | HD | F | 72 | 0 |
| 1 | MB | HD | F | 72 | 0 |
| 1 | MB | HD | F | 72 | 0 |
| 1 | MB | HD | F | 72 | 0 |
| 1 | MB | HD | F | 72 | 0 |
| 1 | MB | HD | F | 72 | 0 |
| 1 | MB | HD | F | 72 | 0 |
| 1 | MB | HD | F | 72 | 0 |
| 1 | MB | HD | M | 18 | 1 |
| 1 | MB | HD | M | 18 | 1 |
| 1 | MB | HD | M | 20 | 1 |
| 1 | MB | HD | M | 20 | 1 |
| 1 | MB | HD | M | 21 | 1 |
| 1 | MB | HD | M | 22 | 1 |
| 1 | MB | HD | M | 22 | 1 |
| 1 | MB | HD | M | 24 | 1 |
| 1 | MB | HD | M | 24 | 1 |
| 1 | MB | HD | M | 24 | 1 |
| 1 | MB | HD | M | 24 | 1 |
| 1 | MB | HD | M | 26 | 1 |
| 1 | MB | HD | M | 26 | 1 |
| 1 | MB | HD | M | 26 | 1 |
| 1 | MB | HD | M | 27 | 1 |
| 1 | MB | HD | M | 28 | 1 |
| 1 | MB | HD | M | 28 | 1 |
| 1 | MB | HD | M | 28 | 1 |
| 1 | MB | HD | M | 28 | 1 |
| 1 | MB | HD | M | 35 | 1 |
| 1 | MB | HD | M | 35 | 1 |
| 1 | MB | HD | M | 35 | 1 |
| 1 | MB | HD | M | 43 | 1 |
| 1 | MB | HD | M | 43 | 1 |
| 1 | MB | HD | M | 72 | 0 |
| 1 | MB | HD | M | 72 | 0 |
| 1 | MB | HD | M | 72 | 0 |
| 1 | MB | HD | M | 72 | 0 |
| 1 | MB | HD | M | 72 | 0 |
| 1 | MB | HD | M | 72 | 0 |
| 1 | MB | HD | M | 72 | 0 |
| 1 | MB | HD | M | 72 | 0 |
| 1 | MB | HD | M | 72 | 0 |
| 1 | MB | HD | M | 72 | 0 |
| 1 | MB | HD | M | 72 | 0 |
| 1 | MB | HD | M | 72 | 0 |
| 1 | MB | HD | M | 72 | 0 |
| 1 | MB | HD | M | 72 | 0 |
| 1 | MB | HD | M | 72 | 0 |
| 1 | MB | HD | M | 72 | 0 |
| 1 | MB | HD | M | 72 | 0 |
| 1 | MB | HD | M | 72 | 0 |
| 1 | MB | HD | M | 72 | 0 |
| 1 | MB | HD | M | 72 | 0 |
| 1 | MB | HD | M | 72 | 0 |
| 1 | MB | HD | M | 72 | 0 |
| 1 | MB | HD | M | 72 | 0 |
| 1 | MB | HD | M | 72 | 0 |
| 1 | MB | HD | M | 72 | 0 |
| 1 | MB | HD | M | 72 | 0 |
| 2 | MB | HD | F | 9 | 1 |
| 2 | MB | HD | F | 9 | 1 |
| 2 | MB | HD | F | 15 | 1 |
| 2 | MB | HD | F | 17.5 | 1 |
| 2 | MB | HD | F | 17.5 | 1 |
| 2 | MB | HD | F | 17.5 | 1 |
| 2 | MB | HD | F | 17.5 | 1 |
| 2 | MB | HD | F | 17.5 | 1 |
| 2 | MB | HD | F | 17.5 | 1 |
| 2 | MB | HD | F | 17.5 | 1 |
| 2 | MB | HD | F | 17.5 | 1 |
| 2 | MB | HD | F | 17.5 | 1 |
| 2 | MB | HD | F | 19 | 1 |
| 2 | MB | HD | F | 19 | 1 |
| 2 | MB | HD | F | 19 | 1 |
| 2 | MB | HD | F | 19 | 1 |
| 2 | MB | HD | F | 21 | 1 |
| 2 | MB | HD | F | 21 | 1 |
| 2 | MB | HD | F | 22 | 1 |
| 2 | MB | HD | F | 22 | 1 |
| 2 | MB | HD | F | 24 | 1 |
| 2 | MB | HD | F | 24 | 1 |
| 2 | MB | HD | F | 25 | 0 |
| 2 | MB | HD | F | 26 | 1 |
| 2 | MB | HD | F | 32 | 1 |
| 2 | MB | HD | F | 32 | 1 |
| 2 | MB | HD | F | 32 | 1 |
| 2 | MB | HD | F | 35 | 1 |
| 2 | MB | HD | F | 35 | 1 |
| 2 | MB | HD | F | 36 | 1 |
| 2 | MB | HD | F | 72 | 0 |
| 2 | MB | HD | F | 72 | 0 |
| 2 | MB | HD | F | 72 | 0 |
| 2 | MB | HD | F | 72 | 0 |
| 2 | MB | HD | F | 72 | 0 |
| 2 | MB | HD | F | 72 | 0 |
| 2 | MB | HD | F | 72 | 0 |
| 2 | MB | HD | F | 72 | 0 |
| 2 | MB | HD | F | 72 | 0 |
| 2 | MB | HD | F | 72 | 0 |
| 2 | MB | HD | F | 72 | 0 |
| 2 | MB | HD | F | 72 | 0 |
| 2 | MB | HD | F | 72 | 0 |
| 2 | MB | HD | F | 72 | 0 |
| 2 | MB | HD | F | 72 | 0 |
| 2 | MB | HD | F | 72 | 0 |
| 2 | MB | HD | F | 72 | 0 |
| 2 | MB | HD | F | 72 | 0 |
| 2 | MB | HD | F | 72 | 0 |
| 2 | MB | HD | F | 72 | 0 |
| 2 | MB | HD | M | 4 | 1 |
| 2 | MB | HD | M | 4 | 1 |
| 2 | MB | HD | M | 7 | 1 |
| 2 | MB | HD | M | 17.5 | 1 |
| 2 | MB | HD | M | 17.5 | 1 |
| 2 | MB | HD | M | 17.5 | 1 |
| 2 | MB | HD | M | 17.5 | 1 |
| 2 | MB | HD | M | 17.5 | 1 |
| 2 | MB | HD | M | 17.5 | 1 |
| 2 | MB | HD | M | 17.5 | 1 |
| 2 | MB | HD | M | 17.5 | 1 |
| 2 | MB | HD | M | 17.5 | 1 |
| 2 | MB | HD | M | 19 | 1 |
| 2 | MB | HD | M | 19 | 1 |
| 2 | MB | HD | M | 19 | 1 |
| 2 | MB | HD | M | 20 | 1 |
| 2 | MB | HD | M | 20 | 1 |
| 2 | MB | HD | M | 20 | 1 |
| 2 | MB | HD | M | 20 | 1 |
| 2 | MB | HD | M | 21 | 1 |
| 2 | MB | HD | M | 21 | 1 |
| 2 | MB | HD | M | 26 | 1 |
| 2 | MB | HD | M | 27 | 1 |
| 2 | MB | HD | M | 35 | 1 |
| 2 | MB | HD | M | 35 | 1 |
| 2 | MB | HD | M | 36 | 1 |
| 2 | MB | HD | M | 51 | 1 |
| 2 | MB | HD | M | 58 | 1 |
| 2 | MB | HD | M | 72 | 0 |
| 2 | MB | HD | M | 72 | 0 |
| 2 | MB | HD | M | 72 | 0 |
| 2 | MB | HD | M | 72 | 0 |
| 2 | MB | HD | M | 72 | 0 |
| 2 | MB | HD | M | 72 | 0 |
| 2 | MB | HD | M | 72 | 0 |
| 2 | MB | HD | M | 72 | 0 |
| 2 | MB | HD | M | 72 | 0 |
| 2 | MB | HD | M | 72 | 0 |
| 2 | MB | HD | M | 72 | 0 |
| 2 | MB | HD | M | 72 | 0 |
| 2 | MB | HD | M | 72 | 0 |
| 2 | MB | HD | M | 72 | 0 |
| 2 | MB | HD | M | 72 | 0 |
| 2 | MB | HD | M | 72 | 0 |
| 2 | MB | HD | M | 72 | 0 |
| 2 | MB | HD | M | 72 | 0 |
| 2 | MB | HD | M | 72 | 0 |
| 2 | MB | HD | M | 72 | 0 |
| 2 | MB | HD | M | 72 | 0 |
| 2 | MB | HD | M | 72 | 0 |
| 3 | MB | HD | F | 7 | 1 |
| 3 | MB | HD | F | 16 | 1 |
| 3 | MB | HD | F | 16 | 1 |
| 3 | MB | HD | F | 18 | 1 |
| 3 | MB | HD | F | 18 | 1 |
| 3 | MB | HD | F | 18 | 1 |
| 3 | MB | HD | F | 18 | 1 |
| 3 | MB | HD | F | 18 | 1 |
| 3 | MB | HD | F | 19 | 1 |
| 3 | MB | HD | F | 19 | 1 |
| 3 | MB | HD | F | 21 | 1 |
| 3 | MB | HD | F | 22 | 1 |
| 3 | MB | HD | F | 23 | 1 |
| 3 | MB | HD | F | 23 | 1 |
| 3 | MB | HD | F | 23 | 1 |
| 3 | MB | HD | F | 23 | 1 |
| 3 | MB | HD | F | 23 | 1 |
| 3 | MB | HD | F | 23 | 1 |
| 3 | MB | HD | F | 24 | 1 |
| 3 | MB | HD | F | 24 | 1 |
| 3 | MB | HD | F | 24 | 1 |
| 3 | MB | HD | F | 24 | 1 |
| 3 | MB | HD | F | 27 | 1 |
| 3 | MB | HD | F | 27 | 1 |
| 3 | MB | HD | F | 28 | 1 |
| 3 | MB | HD | F | 28 | 1 |
| 3 | MB | HD | F | 35 | 1 |
| 3 | MB | HD | F | 38 | 1 |
| 3 | MB | HD | F | 52 | 0 |
| 3 | MB | HD | F | 90 | 0 |
| 3 | MB | HD | F | 90 | 0 |
| 3 | MB | HD | F | 90 | 0 |
| 3 | MB | HD | F | 90 | 0 |
| 3 | MB | HD | F | 90 | 0 |
| 3 | MB | HD | F | 90 | 0 |
| 3 | MB | HD | F | 90 | 0 |
| 3 | MB | HD | F | 90 | 0 |
| 3 | MB | HD | F | 90 | 0 |
| 3 | MB | HD | F | 90 | 0 |
| 3 | MB | HD | F | 90 | 0 |
| 3 | MB | HD | F | 90 | 0 |
| 3 | MB | HD | F | 90 | 0 |
| 3 | MB | HD | F | 90 | 0 |
| 3 | MB | HD | F | 90 | 0 |
| 3 | MB | HD | F | 90 | 0 |
| 3 | MB | HD | F | 90 | 0 |
| 3 | MB | HD | F | 90 | 0 |
| 3 | MB | HD | F | 90 | 0 |
| 3 | MB | HD | F | 90 | 0 |
| 3 | MB | HD | F | 90 | 0 |
| 3 | MB | HD | M | 0 | 1 |
| 3 | MB | HD | M | 16 | 1 |
| 3 | MB | HD | M | 16 | 1 |
| 3 | MB | HD | M | 16 | 1 |
| 3 | MB | HD | M | 17 | 1 |
| 3 | MB | HD | M | 17 | 1 |
| 3 | MB | HD | M | 17 | 1 |
| 3 | MB | HD | M | 17 | 1 |
| 3 | MB | HD | M | 18 | 1 |
| 3 | MB | HD | M | 18 | 1 |
| 3 | MB | HD | M | 18 | 1 |
| 3 | MB | HD | M | 18 | 1 |
| 3 | MB | HD | M | 18 | 1 |
| 3 | MB | HD | M | 19 | 1 |
| 3 | MB | HD | M | 19 | 1 |
| 3 | MB | HD | M | 19 | 1 |
| 3 | MB | HD | M | 19 | 1 |
| 3 | MB | HD | M | 20 | 1 |
| 3 | MB | HD | M | 21 | 1 |
| 3 | MB | HD | M | 21 | 1 |
| 3 | MB | HD | M | 23 | 1 |
| 3 | MB | HD | M | 24 | 1 |
| 3 | MB | HD | M | 24 | 1 |
| 3 | MB | HD | M | 35 | 1 |
| 3 | MB | HD | M | 36 | 1 |
| 3 | MB | HD | M | 38 | 1 |
| 3 | MB | HD | M | 43.5 | 1 |
| 3 | MB | HD | M | 43.5 | 1 |
| 3 | MB | HD | M | 90 | 0 |
| 3 | MB | HD | M | 90 | 0 |
| 3 | MB | HD | M | 90 | 0 |
| 3 | MB | HD | M | 90 | 0 |
| 3 | MB | HD | M | 90 | 0 |
| 3 | MB | HD | M | 90 | 0 |
| 3 | MB | HD | M | 90 | 0 |
| 3 | MB | HD | M | 90 | 0 |
| 3 | MB | HD | M | 90 | 0 |
| 3 | MB | HD | M | 90 | 0 |
| 3 | MB | HD | M | 90 | 0 |
| 3 | MB | HD | M | 90 | 0 |
| 3 | MB | HD | M | 90 | 0 |
| 3 | MB | HD | M | 90 | 0 |
| 3 | MB | HD | M | 90 | 0 |
| 3 | MB | HD | M | 90 | 0 |
| 3 | MB | HD | M | 90 | 0 |
| 3 | MB | HD | M | 90 | 0 |
| 3 | MB | HD | M | 90 | 0 |
| 3 | MB | HD | M | 90 | 0 |
| 3 | MB | HD | M | 90 | 0 |
| 3 | MB | HD | M | 90 | 0 |
| 4 | MB | HD | F | 0 | 1 |
| 4 | MB | HD | F | 18 | 1 |
| 4 | MB | HD | F | 19 | 1 |
| 4 | MB | HD | F | 20 | 1 |
| 4 | MB | HD | F | 20 | 1 |
| 4 | MB | HD | F | 20 | 1 |
| 4 | MB | HD | F | 20 | 1 |
| 4 | MB | HD | F | 21 | 1 |
| 4 | MB | HD | F | 21 | 1 |
| 4 | MB | HD | F | 21 | 1 |
| 4 | MB | HD | F | 23 | 1 |
| 4 | MB | HD | F | 24 | 1 |
| 4 | MB | HD | F | 24 | 1 |
| 4 | MB | HD | F | 25 | 1 |
| 4 | MB | HD | F | 25 | 1 |
| 4 | MB | HD | F | 26 | 1 |
| 4 | MB | HD | F | 26 | 1 |
| 4 | MB | HD | F | 27 | 1 |
| 4 | MB | HD | F | 28 | 1 |
| 4 | MB | HD | F | 29 | 1 |
| 4 | MB | HD | F | 33 | 1 |
| 4 | MB | HD | F | 34.5 | 1 |
| 4 | MB | HD | F | 40 | 1 |
| 4 | MB | HD | F | 40 | 1 |
| 4 | MB | HD | F | 72 | 1 |
| 4 | MB | HD | F | 93 | 0 |
| 4 | MB | HD | F | 93 | 0 |
| 4 | MB | HD | F | 93 | 0 |
| 4 | MB | HD | F | 93 | 0 |
| 4 | MB | HD | F | 93 | 0 |
| 4 | MB | HD | F | 93 | 0 |
| 4 | MB | HD | F | 93 | 0 |
| 4 | MB | HD | F | 93 | 0 |
| 4 | MB | HD | F | 93 | 0 |
| 4 | MB | HD | F | 93 | 0 |
| 4 | MB | HD | F | 93 | 0 |
| 4 | MB | HD | F | 93 | 0 |
| 4 | MB | HD | F | 93 | 0 |
| 4 | MB | HD | F | 93 | 0 |
| 4 | MB | HD | F | 93 | 0 |
| 4 | MB | HD | F | 93 | 0 |
| 4 | MB | HD | F | 93 | 0 |
| 4 | MB | HD | F | 93 | 0 |
| 4 | MB | HD | F | 93 | 0 |
| 4 | MB | HD | F | 93 | 0 |
| 4 | MB | HD | F | 93 | 0 |
| 4 | MB | HD | F | 93 | 0 |
| 4 | MB | HD | F | 93 | 0 |
| 4 | MB | HD | F | 93 | 0 |
| 4 | MB | HD | F | 93 | 0 |
| 4 | MB | HD | M | 0 | 1 |
| 4 | MB | HD | M | 2 | 1 |
| 4 | MB | HD | M | 17 | 1 |
| 4 | MB | HD | M | 17 | 1 |
| 4 | MB | HD | M | 17 | 1 |
| 4 | MB | HD | M | 18 | 1 |
| 4 | MB | HD | M | 18 | 1 |
| 4 | MB | HD | M | 19 | 1 |
| 4 | MB | HD | M | 20 | 1 |
| 4 | MB | HD | M | 20 | 1 |
| 4 | MB | HD | M | 21 | 1 |
| 4 | MB | HD | M | 21 | 1 |
| 4 | MB | HD | M | 21 | 1 |
| 4 | MB | HD | M | 21 | 1 |
| 4 | MB | HD | M | 22 | 1 |
| 4 | MB | HD | M | 22 | 1 |
| 4 | MB | HD | M | 23 | 1 |
| 4 | MB | HD | M | 24 | 1 |
| 4 | MB | HD | M | 25 | 1 |
| 4 | MB | HD | M | 29 | 1 |
| 4 | MB | HD | M | 29 | 1 |
| 4 | MB | HD | M | 42.5 | 1 |
| 4 | MB | HD | M | 49.5 | 1 |
| 4 | MB | HD | M | 61.5 | 1 |
| 4 | MB | HD | M | 93 | 0 |
| 4 | MB | HD | M | 93 | 0 |
| 4 | MB | HD | M | 93 | 0 |
| 4 | MB | HD | M | 93 | 0 |
| 4 | MB | HD | M | 93 | 0 |
| 4 | MB | HD | M | 93 | 0 |
| 4 | MB | HD | M | 93 | 0 |
| 4 | MB | HD | M | 93 | 0 |
| 4 | MB | HD | M | 93 | 0 |
| 4 | MB | HD | M | 93 | 0 |
| 4 | MB | HD | M | 93 | 0 |
| 4 | MB | HD | M | 93 | 0 |
| 4 | MB | HD | M | 93 | 0 |
| 4 | MB | HD | M | 93 | 0 |
| 4 | MB | HD | M | 93 | 0 |
| 4 | MB | HD | M | 93 | 0 |
| 4 | MB | HD | M | 93 | 0 |
| 4 | MB | HD | M | 93 | 0 |
| 4 | MB | HD | M | 93 | 0 |
| 4 | MB | HD | M | 93 | 0 |
| 4 | MB | HD | M | 93 | 0 |
| 4 | MB | HD | M | 93 | 0 |
| 4 | MB | HD | M | 93 | 0 |
| 4 | MB | HD | M | 93 | 0 |
| 4 | MB | HD | M | 93 | 0 |
| 4 | MB | HD | M | 93 | 0 |
| 3 | MB | LD | F | 19 | 1 |
| 3 | MB | LD | F | 20 | 1 |
| 3 | MB | LD | F | 20 | 1 |
| 3 | MB | LD | F | 20 | 1 |
| 3 | MB | LD | F | 21 | 1 |
| 3 | MB | LD | F | 21 | 1 |
| 3 | MB | LD | F | 21 | 1 |
| 3 | MB | LD | F | 22 | 1 |
| 3 | MB | LD | F | 22 | 1 |
| 3 | MB | LD | F | 22 | 1 |
| 3 | MB | LD | F | 22 | 1 |
| 3 | MB | LD | F | 23 | 1 |
| 3 | MB | LD | F | 23 | 1 |
| 3 | MB | LD | F | 24 | 1 |
| 3 | MB | LD | F | 24 | 1 |
| 3 | MB | LD | F | 26 | 1 |
| 3 | MB | LD | F | 26 | 1 |
| 3 | MB | LD | F | 27 | 1 |
| 3 | MB | LD | F | 30 | 1 |
| 3 | MB | LD | F | 31 | 1 |
| 3 | MB | LD | F | 31 | 1 |
| 3 | MB | LD | F | 35 | 1 |
| 3 | MB | LD | F | 39 | 1 |
| 3 | MB | LD | F | 90 | 0 |
| 3 | MB | LD | F | 90 | 0 |
| 3 | MB | LD | F | 90 | 0 |
| 3 | MB | LD | F | 90 | 0 |
| 3 | MB | LD | F | 90 | 0 |
| 3 | MB | LD | F | 90 | 0 |
| 3 | MB | LD | F | 90 | 0 |
| 3 | MB | LD | F | 90 | 0 |
| 3 | MB | LD | F | 90 | 0 |
| 3 | MB | LD | F | 90 | 0 |
| 3 | MB | LD | F | 90 | 0 |
| 3 | MB | LD | F | 90 | 0 |
| 3 | MB | LD | F | 90 | 0 |
| 3 | MB | LD | F | 90 | 0 |
| 3 | MB | LD | F | 90 | 0 |
| 3 | MB | LD | F | 90 | 0 |
| 3 | MB | LD | F | 90 | 0 |
| 3 | MB | LD | F | 90 | 0 |
| 3 | MB | LD | F | 90 | 0 |
| 3 | MB | LD | F | 90 | 0 |
| 3 | MB | LD | F | 90 | 0 |
| 3 | MB | LD | F | 90 | 0 |
| 3 | MB | LD | F | 90 | 0 |
| 3 | MB | LD | F | 90 | 0 |
| 3 | MB | LD | F | 90 | 0 |
| 3 | MB | LD | F | 90 | 0 |
| 3 | MB | LD | F | 90 | 0 |
| 3 | MB | LD | M | 20 | 1 |
| 3 | MB | LD | M | 20 | 1 |
| 3 | MB | LD | M | 20 | 1 |
| 3 | MB | LD | M | 20 | 1 |
| 3 | MB | LD | M | 20 | 1 |
| 3 | MB | LD | M | 21 | 1 |
| 3 | MB | LD | M | 21 | 1 |
| 3 | MB | LD | M | 23 | 1 |
| 3 | MB | LD | M | 23 | 1 |
| 3 | MB | LD | M | 24 | 1 |
| 3 | MB | LD | M | 24 | 1 |
| 3 | MB | LD | M | 24 | 1 |
| 3 | MB | LD | M | 24 | 1 |
| 3 | MB | LD | M | 25 | 1 |
| 3 | MB | LD | M | 26 | 1 |
| 3 | MB | LD | M | 29 | 1 |
| 3 | MB | LD | M | 35 | 1 |
| 3 | MB | LD | M | 36 | 1 |
| 3 | MB | LD | M | 38 | 1 |
| 3 | MB | LD | M | 38 | 1 |
| 3 | MB | LD | M | 38 | 1 |
| 3 | MB | LD | M | 45.5 | 1 |
| 3 | MB | LD | M | 66 | 1 |
| 3 | MB | LD | M | 90 | 0 |
| 3 | MB | LD | M | 90 | 0 |
| 3 | MB | LD | M | 90 | 0 |
| 3 | MB | LD | M | 90 | 0 |
| 3 | MB | LD | M | 90 | 0 |
| 3 | MB | LD | M | 90 | 0 |
| 3 | MB | LD | M | 90 | 0 |
| 3 | MB | LD | M | 90 | 0 |
| 3 | MB | LD | M | 90 | 0 |
| 3 | MB | LD | M | 90 | 0 |
| 3 | MB | LD | M | 90 | 0 |
| 3 | MB | LD | M | 90 | 0 |
| 3 | MB | LD | M | 90 | 0 |
| 3 | MB | LD | M | 90 | 0 |
| 3 | MB | LD | M | 90 | 0 |
| 3 | MB | LD | M | 90 | 0 |
| 3 | MB | LD | M | 90 | 0 |
| 3 | MB | LD | M | 90 | 0 |
| 3 | MB | LD | M | 90 | 0 |
| 3 | MB | LD | M | 90 | 0 |
| 3 | MB | LD | M | 90 | 0 |
| 3 | MB | LD | M | 90 | 0 |
| 3 | MB | LD | M | 90 | 0 |
| 3 | MB | LD | M | 90 | 0 |
| 3 | MB | LD | M | 90 | 0 |
| 3 | MB | LD | M | 90 | 0 |
| 3 | MB | LD | M | 90 | 0 |
| 1 | MB | LD | F | 18 | 1 |
| 1 | MB | LD | F | 21 | 1 |
| 1 | MB | LD | F | 22 | 1 |
| 1 | MB | LD | F | 22 | 1 |
| 1 | MB | LD | F | 22 | 1 |
| 1 | MB | LD | F | 23 | 1 |
| 1 | MB | LD | F | 23 | 1 |
| 1 | MB | LD | F | 24 | 1 |
| 1 | MB | LD | F | 25 | 1 |
| 1 | MB | LD | F | 25 | 1 |
| 1 | MB | LD | F | 25 | 1 |
| 1 | MB | LD | F | 25 | 1 |
| 1 | MB | LD | F | 25 | 1 |
| 1 | MB | LD | F | 26 | 1 |
| 1 | MB | LD | F | 26 | 1 |
| 1 | MB | LD | F | 26 | 1 |
| 1 | MB | LD | F | 26 | 1 |
| 1 | MB | LD | F | 27 | 1 |
| 1 | MB | LD | F | 27 | 1 |
| 1 | MB | LD | F | 29 | 1 |
| 1 | MB | LD | F | 29 | 1 |
| 1 | MB | LD | F | 29 | 1 |
| 1 | MB | LD | F | 33 | 1 |
| 1 | MB | LD | F | 33 | 1 |
| 1 | MB | LD | F | 44 | 1 |
| 1 | MB | LD | F | 44 | 1 |
| 1 | MB | LD | F | 49 | 1 |
| 1 | MB | LD | F | 72 | 0 |
| 1 | MB | LD | F | 72 | 0 |
| 1 | MB | LD | F | 72 | 0 |
| 1 | MB | LD | F | 72 | 0 |
| 1 | MB | LD | F | 72 | 0 |
| 1 | MB | LD | F | 72 | 0 |
| 1 | MB | LD | F | 72 | 0 |
| 1 | MB | LD | F | 72 | 0 |
| 1 | MB | LD | F | 72 | 0 |
| 1 | MB | LD | F | 72 | 0 |
| 1 | MB | LD | F | 72 | 0 |
| 1 | MB | LD | F | 72 | 0 |
| 1 | MB | LD | F | 72 | 0 |
| 1 | MB | LD | F | 72 | 0 |
| 1 | MB | LD | F | 72 | 0 |
| 1 | MB | LD | F | 72 | 0 |
| 1 | MB | LD | F | 72 | 0 |
| 1 | MB | LD | F | 72 | 0 |
| 1 | MB | LD | F | 72 | 0 |
| 1 | MB | LD | F | 72 | 0 |
| 1 | MB | LD | F | 72 | 0 |
| 1 | MB | LD | F | 72 | 0 |
| 1 | MB | LD | F | 72 | 0 |
| 1 | MB | LD | M | 18 | 1 |
| 1 | MB | LD | M | 18 | 1 |
| 1 | MB | LD | M | 20 | 1 |
| 1 | MB | LD | M | 22 | 1 |
| 1 | MB | LD | M | 23 | 1 |
| 1 | MB | LD | M | 23 | 1 |
| 1 | MB | LD | M | 23 | 1 |
| 1 | MB | LD | M | 23 | 1 |
| 1 | MB | LD | M | 23 | 1 |
| 1 | MB | LD | M | 24 | 1 |
| 1 | MB | LD | M | 24 | 1 |
| 1 | MB | LD | M | 25 | 1 |
| 1 | MB | LD | M | 25 | 1 |
| 1 | MB | LD | M | 25 | 1 |
| 1 | MB | LD | M | 26 | 1 |
| 1 | MB | LD | M | 26 | 1 |
| 1 | MB | LD | M | 27 | 1 |
| 1 | MB | LD | M | 27 | 1 |
| 1 | MB | LD | M | 27 | 1 |
| 1 | MB | LD | M | 27 | 1 |
| 1 | MB | LD | M | 28 | 1 |
| 1 | MB | LD | M | 44 | 1 |
| 1 | MB | LD | M | 44 | 1 |
| 1 | MB | LD | M | 72 | 0 |
| 1 | MB | LD | M | 72 | 0 |
| 1 | MB | LD | M | 72 | 0 |
| 1 | MB | LD | M | 72 | 0 |
| 1 | MB | LD | M | 72 | 0 |
| 1 | MB | LD | M | 72 | 0 |
| 1 | MB | LD | M | 72 | 0 |
| 1 | MB | LD | M | 72 | 0 |
| 1 | MB | LD | M | 72 | 0 |
| 1 | MB | LD | M | 72 | 0 |
| 1 | MB | LD | M | 72 | 0 |
| 1 | MB | LD | M | 72 | 0 |
| 1 | MB | LD | M | 72 | 0 |
| 1 | MB | LD | M | 72 | 0 |
| 1 | MB | LD | M | 72 | 0 |
| 1 | MB | LD | M | 72 | 0 |
| 1 | MB | LD | M | 72 | 0 |
| 1 | MB | LD | M | 72 | 0 |
| 1 | MB | LD | M | 72 | 0 |
| 1 | MB | LD | M | 72 | 0 |
| 1 | MB | LD | M | 72 | 0 |
| 1 | MB | LD | M | 72 | 0 |
| 1 | MB | LD | M | 72 | 0 |
| 1 | MB | LD | M | 72 | 0 |
| 1 | MB | LD | M | 72 | 0 |
| 1 | MB | LD | M | 72 | 0 |
| 1 | MB | LD | M | 72 | 0 |
| 2 | MB | LD | F | 1 | 1 |
| 2 | MB | LD | F | 15 | 1 |
| 2 | MB | LD | F | 17.5 | 1 |
| 2 | MB | LD | F | 19 | 1 |
| 2 | MB | LD | F | 19 | 1 |
| 2 | MB | LD | F | 20 | 1 |
| 2 | MB | LD | F | 20 | 1 |
| 2 | MB | LD | F | 21 | 1 |
| 2 | MB | LD | F | 21 | 1 |
| 2 | MB | LD | F | 21 | 1 |
| 2 | MB | LD | F | 21 | 1 |
| 2 | MB | LD | F | 21 | 1 |
| 2 | MB | LD | F | 22 | 1 |
| 2 | MB | LD | F | 22 | 1 |
| 2 | MB | LD | F | 25 | 1 |
| 2 | MB | LD | F | 27 | 1 |
| 2 | MB | LD | F | 27 | 1 |
| 2 | MB | LD | F | 27 | 1 |
| 2 | MB | LD | F | 27 | 1 |
| 2 | MB | LD | F | 29 | 1 |
| 2 | MB | LD | F | 30 | 1 |
| 2 | MB | LD | F | 32 | 1 |
| 2 | MB | LD | F | 34 | 1 |
| 2 | MB | LD | F | 35 | 1 |
| 2 | MB | LD | F | 35 | 1 |
| 2 | MB | LD | F | 47 | 1 |
| 2 | MB | LD | F | 72 | 0 |
| 2 | MB | LD | F | 72 | 0 |
| 2 | MB | LD | F | 72 | 0 |
| 2 | MB | LD | F | 72 | 0 |
| 2 | MB | LD | F | 72 | 0 |
| 2 | MB | LD | F | 72 | 0 |
| 2 | MB | LD | F | 72 | 0 |
| 2 | MB | LD | F | 72 | 0 |
| 2 | MB | LD | F | 72 | 0 |
| 2 | MB | LD | F | 72 | 0 |
| 2 | MB | LD | F | 72 | 0 |
| 2 | MB | LD | F | 72 | 0 |
| 2 | MB | LD | F | 72 | 0 |
| 2 | MB | LD | F | 72 | 0 |
| 2 | MB | LD | F | 72 | 0 |
| 2 | MB | LD | F | 72 | 0 |
| 2 | MB | LD | F | 72 | 0 |
| 2 | MB | LD | F | 72 | 0 |
| 2 | MB | LD | F | 72 | 0 |
| 2 | MB | LD | F | 72 | 0 |
| 2 | MB | LD | F | 72 | 0 |
| 2 | MB | LD | F | 72 | 0 |
| 2 | MB | LD | F | 72 | 0 |
| 2 | MB | LD | F | 72 | 0 |
| 2 | MB | LD | M | 17.5 | 1 |
| 2 | MB | LD | M | 17.5 | 1 |
| 2 | MB | LD | M | 20 | 1 |
| 2 | MB | LD | M | 20 | 1 |
| 2 | MB | LD | M | 20 | 1 |
| 2 | MB | LD | M | 20 | 1 |
| 2 | MB | LD | M | 20 | 1 |
| 2 | MB | LD | M | 20 | 1 |
| 2 | MB | LD | M | 20 | 1 |
| 2 | MB | LD | M | 20 | 1 |
| 2 | MB | LD | M | 21 | 1 |
| 2 | MB | LD | M | 21 | 1 |
| 2 | MB | LD | M | 21 | 1 |
| 2 | MB | LD | M | 22 | 1 |
| 2 | MB | LD | M | 22 | 1 |
| 2 | MB | LD | M | 22 | 1 |
| 2 | MB | LD | M | 22 | 1 |
| 2 | MB | LD | M | 23 | 1 |
| 2 | MB | LD | M | 24 | 1 |
| 2 | MB | LD | M | 24 | 1 |
| 2 | MB | LD | M | 25 | 1 |
| 2 | MB | LD | M | 25 | 1 |
| 2 | MB | LD | M | 26 | 1 |
| 2 | MB | LD | M | 26 | 1 |
| 2 | MB | LD | M | 27 | 1 |
| 2 | MB | LD | M | 27 | 1 |
| 2 | MB | LD | M | 27 | 1 |
| 2 | MB | LD | M | 27 | 1 |
| 2 | MB | LD | M | 27 | 1 |
| 2 | MB | LD | M | 29 | 1 |
| 2 | MB | LD | M | 39 | 1 |
| 2 | MB | LD | M | 72 | 0 |
| 2 | MB | LD | M | 72 | 0 |
| 2 | MB | LD | M | 72 | 0 |
| 2 | MB | LD | M | 72 | 0 |
| 2 | MB | LD | M | 72 | 0 |
| 2 | MB | LD | M | 72 | 0 |
| 2 | MB | LD | M | 72 | 0 |
| 2 | MB | LD | M | 72 | 0 |
| 2 | MB | LD | M | 72 | 0 |
| 2 | MB | LD | M | 72 | 0 |
| 2 | MB | LD | M | 72 | 0 |
| 2 | MB | LD | M | 72 | 0 |
| 2 | MB | LD | M | 72 | 0 |
| 2 | MB | LD | M | 72 | 0 |
| 2 | MB | LD | M | 72 | 0 |
| 2 | MB | LD | M | 72 | 0 |
| 2 | MB | LD | M | 72 | 0 |
| 2 | MB | LD | M | 72 | 0 |
| 2 | MB | LD | M | 72 | 0 |
| 4 | MB | LD | F | 20 | 1 |
| 4 | MB | LD | F | 21 | 1 |
| 4 | MB | LD | F | 21 | 1 |
| 4 | MB | LD | F | 22 | 1 |
| 4 | MB | LD | F | 22 | 1 |
| 4 | MB | LD | F | 22 | 1 |
| 4 | MB | LD | F | 24 | 1 |
| 4 | MB | LD | F | 24 | 1 |
| 4 | MB | LD | F | 24 | 1 |
| 4 | MB | LD | F | 24 | 1 |
| 4 | MB | LD | F | 24 | 1 |
| 4 | MB | LD | F | 24 | 1 |
| 4 | MB | LD | F | 24 | 1 |
| 4 | MB | LD | F | 25 | 1 |
| 4 | MB | LD | F | 25 | 1 |
| 4 | MB | LD | F | 25 | 1 |
| 4 | MB | LD | F | 25 | 1 |
| 4 | MB | LD | F | 26 | 1 |
| 4 | MB | LD | F | 26 | 1 |
| 4 | MB | LD | F | 26 | 1 |
| 4 | MB | LD | F | 26 | 1 |
| 4 | MB | LD | F | 26 | 1 |
| 4 | MB | LD | F | 27 | 1 |
| 4 | MB | LD | F | 27 | 1 |
| 4 | MB | LD | F | 28 | 1 |
| 4 | MB | LD | F | 29 | 1 |
| 4 | MB | LD | F | 30 | 1 |
| 4 | MB | LD | F | 30 | 1 |
| 4 | MB | LD | F | 40 | 1 |
| 4 | MB | LD | F | 93 | 0 |
| 4 | MB | LD | F | 93 | 0 |
| 4 | MB | LD | F | 93 | 0 |
| 4 | MB | LD | F | 93 | 0 |
| 4 | MB | LD | F | 93 | 0 |
| 4 | MB | LD | F | 93 | 0 |
| 4 | MB | LD | F | 93 | 0 |
| 4 | MB | LD | F | 93 | 0 |
| 4 | MB | LD | F | 93 | 0 |
| 4 | MB | LD | F | 93 | 0 |
| 4 | MB | LD | F | 93 | 0 |
| 4 | MB | LD | F | 93 | 0 |
| 4 | MB | LD | F | 93 | 0 |
| 4 | MB | LD | F | 93 | 0 |
| 4 | MB | LD | F | 93 | 0 |
| 4 | MB | LD | F | 93 | 0 |
| 4 | MB | LD | F | 93 | 0 |
| 4 | MB | LD | F | 93 | 0 |
| 4 | MB | LD | F | 93 | 0 |
| 4 | MB | LD | F | 93 | 0 |
| 4 | MB | LD | F | 93 | 0 |
| 4 | MB | LD | M | 0 | 1 |
| 4 | MB | LD | M | 16 | 1 |
| 4 | MB | LD | M | 21 | 1 |
| 4 | MB | LD | M | 22 | 1 |
| 4 | MB | LD | M | 22 | 1 |
| 4 | MB | LD | M | 22 | 1 |
| 4 | MB | LD | M | 22 | 1 |
| 4 | MB | LD | M | 22 | 1 |
| 4 | MB | LD | M | 23 | 1 |
| 4 | MB | LD | M | 23 | 1 |
| 4 | MB | LD | M | 25 | 1 |
| 4 | MB | LD | M | 25 | 1 |
| 4 | MB | LD | M | 25 | 1 |
| 4 | MB | LD | M | 25 | 1 |
| 4 | MB | LD | M | 26 | 1 |
| 4 | MB | LD | M | 26 | 1 |
| 4 | MB | LD | M | 26 | 1 |
| 4 | MB | LD | M | 28 | 1 |
| 4 | MB | LD | M | 28 | 1 |
| 4 | MB | LD | M | 28 | 1 |
| 4 | MB | LD | M | 29 | 1 |
| 4 | MB | LD | M | 29 | 1 |
| 4 | MB | LD | M | 31 | 1 |
| 4 | MB | LD | M | 43 | 1 |
| 4 | MB | LD | M | 55.5 | 1 |
| 4 | MB | LD | M | 93 | 0 |
| 4 | MB | LD | M | 93 | 0 |
| 4 | MB | LD | M | 93 | 0 |
| 4 | MB | LD | M | 93 | 0 |
| 4 | MB | LD | M | 93 | 0 |
| 4 | MB | LD | M | 93 | 0 |
| 4 | MB | LD | M | 93 | 0 |
| 4 | MB | LD | M | 93 | 0 |
| 4 | MB | LD | M | 93 | 0 |
| 4 | MB | LD | M | 93 | 0 |
| 4 | MB | LD | M | 93 | 0 |
| 4 | MB | LD | M | 93 | 0 |
| 4 | MB | LD | M | 93 | 0 |
| 4 | MB | LD | M | 93 | 0 |
| 4 | MB | LD | M | 93 | 0 |
| 4 | MB | LD | M | 93 | 0 |
| 4 | MB | LD | M | 93 | 0 |
| 4 | MB | LD | M | 93 | 0 |
| 4 | MB | LD | M | 93 | 0 |
| 4 | MB | LD | M | 93 | 0 |
| 4 | MB | LD | M | 93 | 0 |
| 4 | MB | LD | M | 93 | 0 |
| 4 | MB | LD | M | 93 | 0 |
| 4 | MB | LD | M | 93 | 0 |
| 4 | MB | LD | M | 93 | 0 |
